# Novel Erlotinib–Chalcone Hybrids Diminish Resistance in Head and Neck Cancer by Inducing Multiple Cell Death Mechanisms

**DOI:** 10.3390/ijms24043456

**Published:** 2023-02-09

**Authors:** József Murányi, Cintia Duró, Bianka Gurbi, István Móra, Attila Varga, Krisztina Németh, József Simon, Miklós Csala, Antal Csámpai

**Affiliations:** 1 Department of Molecular Biology, Semmelweis University, Tűzoltó u. 37-47, H-1094 Budapest, Hungary; 2 Department of Organic Chemistry, Eötvös Loránd University (ELTE), Pázmány P. Sétány 1/A, H-1117 Budapest, Hungary; 3 MS Metabolomics Research Group, Centre for Structural Study, Research Centre for Natural Sciences, Eötvös Loránd Research Network, Magyar Tudósok Krt. 2, H-1117 Budapest, Hungary; 4 Research Group of Analytical Chemistry, University of Pannonia, Egyetem utca 10, H-8200 Veszprém, Hungary

**Keywords:** erlotinib, chalcone, hybrid, HNSCC, necrosis, apoptosis, paclitaxel, resistance, cancer

## Abstract

In a search for novel therapeutic options for head and neck squamous cell carcinomas (HNSCCs) generally treated with limited therapeutic success, we synthesized a series of novel erlotinib–chalcone molecular hybrids with 1,2,3-triazole and alkyne linkers and evaluated them for their anticancer activity on Fadu, Detroit 562 and SCC-25 HNSCC cell lines. Time- and dose-dependent cell viability measurements disclosed a significantly increased efficiency of the hybrids compared to the 1:1 combination of erlotinib and a reference chalcone. The clonogenic assay demonstrated that hybrids eradicate HNSCC cells in low micromolar concentrations. Experiments focusing on potential molecular targets indicate that the hybrids trigger the anticancer effect by a complementary mechanism of action that is independent of the canonical targets of their molecular fragments. Confocal microscopic imaging and real-time apoptosis/necrosis detection assay pointed to slightly different cell death mechanisms induced by the most prominent triazole- and alkyne-tethered hybrids (**6a** and **13**, respectively). While **6a** featured the lowest IC_50_ values on each of the three HNSCC cell lines, in Detroit 562 cells, this hybrid induced necrosis more markedly compared to **13**. The therapeutic potential indicated by the observed anticancer efficacy of our selected hybrid molecules validates the concept of development and justifies further investigation to reveal the underlying mechanism of action.

## 1. Introduction

Head and neck squamous cell carcinomas (HNSCCs) represent the sixth most common cancer worldwide, accounting for approximately 660,000 new diagnoses and 325,000 deaths annually. The main risk factors are tobacco and alcohol consumption, but infection with human papillomavirus or Epstein–Barr virus is also responsible for the development of the disease [1,2].

The topological diversity and the high genomic heterogeneity of the individual HNSCC tumors make it difficult to identify predictive biomarkers of therapeutic value. The principal modalities of curative therapy for HNSCCs are surgery, radiation, and systemic therapy [3].

Since human epidermal growth factor receptor (EGFR) has a pivotal role in carcinogenesis and HNSCCs show significantly increased EGFR expression, several EGFR inhibitors were developed and tested for HNSCC treatment in the past few decades [4]. The currently available types of EGFR inhibitors are monoclonal antibodies (e.g., cetuximab) and tyrosine kinase inhibitors (such as gefitinib, erlotinib, afatinib and lapatinib). Despite effective EGFR inhibition, these compounds have relatively low response rates in HNSCC patients in monotherapy, and the development of resistance is a general problem, which compromises the chances of cure. EGFR antisense DNA represents a promising approach by inhibition of EGFR expression in tumor cells; however, a therapeutic breakthrough has yet to be achieved. Therefore, the combination of EGFR inhibitors with other therapeutic agents has emerged as a promising strategy to overcome resistance and improve clinical outcomes [5].

Although recent advances in immunotherapy led to a better survival rate than achievable by conventional chemotherapy in a small fraction of HNSCC patients, the overall clinical response rate is still below that observed in many other tumor types. It follows that the necessary improvement in the survival of HNSCC patients requires novel strategies based on the identification of better molecular targets and therapeutic agents [3].

Fragment-based drug discovery is a promising recent approach to developing potent hybrid compounds for cancer treatment by coupling different pharmacophore residues into new multitarget anticancer agents [6,7,8]. The enhanced efficiency of hybrid anticancer drugs with more than one molecular target in the cells lies in the fact that they trigger multiple signal transduction pathways, finally leading to the multiplied stimulation of cell death. In principle, hybrid anticancer agents might have a real potential to overcome certain disadvantages of single cancer drugs, such as resistance and adverse effects.

**Scheme 1:**
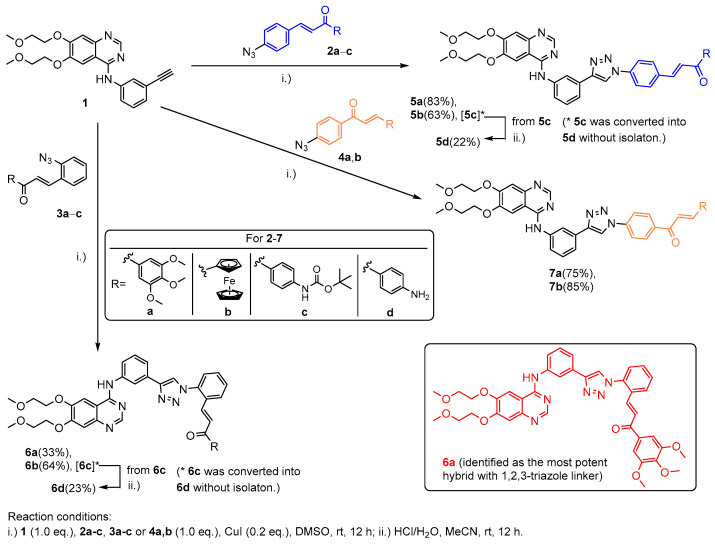
Synthesis of the group of erlotinib–chalcone hybrids with 1,2,3-triazole linker.

In attempting to target HNSCCs with enhanced efficacy, we envisaged exploring the concept of fragment-based drug design for the development of novel hybrid compounds. Accordingly, erlotinib (**1**, Scheme 1) was selected as the fragment displaying EGFR inhibitory activity with a terminal alkyne moiety that makes this molecule feasible for well-established coupling reactions used for chemical hybridizations.

Following the strategy of fragment-based drug design, we selected representative types of chalcones to be incorporated in erlotinib-based hybrid molecules as an additional pharmacophore unit with significant potency in contributing to antiproliferative activity. As it has been extensively reviewed, chalcone derivatives represent a highly potent class of anticancer drug candidates featuring manifold mechanisms of action in cancer cells [9,10,11,12]. For instance, a great number of their representatives were found to induce significant cell cycle arrest at the G2/M phase leading to apoptosis; moreover, they exhibited higher efficacy due to their inhibitory activity in tubulin polymerization, enzyme dynamics [13] and signal transductions induced by nuclear factor κB [14]. Highlighting the importance of cell cycle arrest, 1,2,3-triazole-tethered cinchona-chalcone hybrids demonstrated substantial antiproliferative activity in the highly treatment-resistant PANC-1 pancreatic carcinoma cells, exerting extensive inhibitory effects in the subG1, S and G2/M phases [15].

It is also of note that diverse chalcone derivatives have a real potency even in the therapy of drug resistance as they display promising in vitro and in vivo effects on both drug-susceptible and drug-resistant cancers while also being capable of acting on a variety of targets, such as aromatase, breast cancer resistance protein (BCRP), vascular endothelial growth factor (VEGF) and ATP binding cassette subfamily G member 2 (ABCG2) [16,17].

In the context of our recent program, it is of particular relevance that focused mechanistic studies identified highly potent 1,2,3-triazole-tethered chalcone hybrids inducing apoptosis, G2/S arrest, inhibition of the ATR-mediated activation of Chk1 and disruption of mitochondrial membrane potential in a panel of human cancer cell lines [18,19]. Mitochondrial damage could be achieved in both drug-sensitive and multidrug-resistant (MDR) lung carcinoma cells by such ferrocene-based triazole-containing cinchona-chalcone hybrids [20], which were also found to exhibit significant antiproliferative activity in HepG2 hepatoma and HT-29 colorectal adenocarcinoma human tumor cell cultures [21].

We envisaged introducing 1,2,3-triazole and alkyne moieties as linkers to tether erlotinib and chalcone fragments in the target hybrid molecules. On the one hand, given the presence of the ethynyl group in erlotinib, this choice of linkers is reasonable from the synthetic aspect, and on the other hand, their introduction in the molecular architecture might significantly attenuate the antiproliferative activity of a hybrid therapeutic agent. Accordingly, the 1,2,3-triazole ring, a privileged pharmacophore unit readily available by “click” chemistry, is capable of forming a variety of noncovalent interactions with diverse enzymes, proteins and receptors by hydrogen bonds as well as van der Waals and dipole–dipole forces [22,23,24,25]; thus, this heterocycle has been extensively utilized as a valuable pharmacophoric motif in bioactive compounds with diverse activities, including antimalarial [26,27,28], antibacterial [29,30], antiviral [31,32] and anticancer [15,18,19,20,21,28,32,33,34] effects. The presented selection of examples from the recent literature underlines that the 1,2,3-triazole ring is worth being incorporated as a linker in the target chalcone-containing hybrids.

Besides the synthetic feasibility of well-established protocols of Sonogashira coupling, the beneficial feature of the introduction of an acetylenic linker into anticancer drug candidates is also justified by characteristic literature examples reporting on alkyne derivatives identified as potent antitumor agents of natural and synthetic origin [35,36,37,38,39,40,41].

Finally, it is of note that our current strategy in fragment-based drug design is further supported by a previous research that demonstrated that both 1,2,3-triazole-linked and alkyne-tethered hybrid molecules with identical terminal pharmacophores display antiproliferative activity in a panel of human cancer cell lines [42].

## 2. Results

### 2.1. Chemistry 

Starting from erlotinib (**1**) and azidochalcones **2a**–**c**, **3a**–**c** and **4a**,**b** as coupling partners, the target hybrids with 1,4-disubstituted 1,2,3-triazole linkers with alternative chalcone constitutions (**5a**,**b**,**d**, **6a**,**b**,**d** and **7a**,**b**, respectively: Scheme 1) were accessed in acceptable-to-good yields (33–85%) by straightforward synthetic routes based on the well-established copper-catalyzed alkyne-azide cycloaddition [43]. The reactions were conducted at room temperature for a prolonged reaction time (12 h) using copper(I) iodide as a catalyst in dimethylsulfoxide (DMSO) that served both as solvent and ligand. The hybrids with a 4-aminophenyl group at the terminal position of the chalcone fragment (**5d** and **6d**) were obtained in low overall yields (22% and 23%, respectively) by standard cycloaddition/acid-catalyzed *N*-deprotection sequence employed in a one-pot protocol without isolation of the *tert*-butoxycarbonyl-protected intermediate (Scheme 1(**5c**) and (**6c**)). The azidochalcone components were prepared by Claisen–Schmidt condensation of the appropriate arylaldehyde and methyl ketone, as described in previous works (**2**–**3a**: [15], **2**–**3b**: [21] and **4a**: [28]) and in the Supplementary Materials (SM) (**2**–**3c** and **4b**: Section S1).

**Scheme 2:**
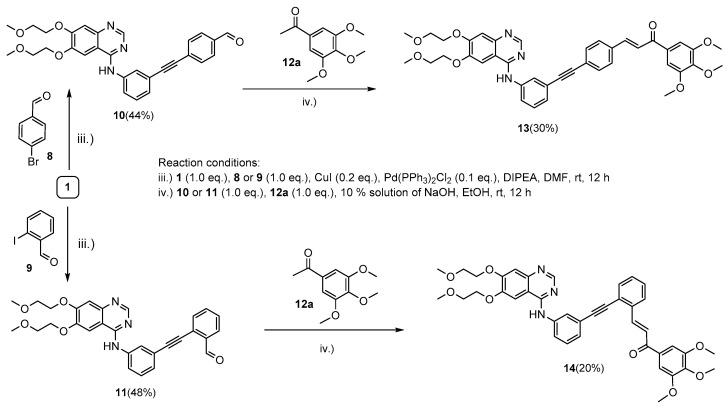
Synthesis of the group of erlotinib–chalcone hybrids with alkyne linker.

**Figure 1:**
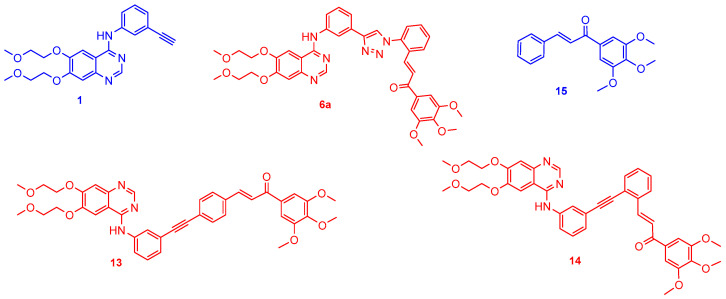
The novel hybrids emerged as the most potent model compounds in the viability assays (**6a**, **13** and **14**), which were selected for further biological studies (red) using erlotinib (**1**) and chalcone (**15**) as reference fragments (blue) exploited in structure design.

Since the initial cell viability inhibition screening (discussed in detail in Section 2.2) identified **6a** as the most active member of the hybrids with the 1,2,3-triazol linker (Scheme 1), we selected 3,4,5-trimethoxyhenyl derivatives **13** and **14** as targeted structures expected to have promising anticancer properties in the series of the possible alkyne-tethered erlotinib–chalcone hybrids. Accordingly, following a simple synthetic route, alkyne-containing aldehyde intermediates **10** and **11** were first obtained in mediocre yields (44% and 48%) by using a well-established protocol of Sonogashira coupling of **1** with 4-bromobenzaldehyde (**8**) and 2-iodobenzaldehyde (**9**), respectively (Scheme 2). In the second step, hybrids **13** and **14** were accessed by base-catalyzed Claisen–Schmidt condensation of 3,4,5-trimethoxyacetophenone **12a** with aldehyde components **10** and **11**, respectively (Scheme 2). The relatively low isolated yields of **13** and **14** might be due to the competing conjugate addition of **12a** to the activated carbon-carbon triple bond in the aldehyde components and/or uncontrolled polymerization processes. Finally, **12a** was also subjected to Claisen–Schmidt condensation with benzaldehyde to afford chalcone **15** as a reference compound [44] (Figure 1).

### 2.2. In Vitro Cell Viability Inhibition Screening of Novel Erlotinib–Chalcone Hybrid Molecules on HNSCC Cell Lines

**Figure 2:**
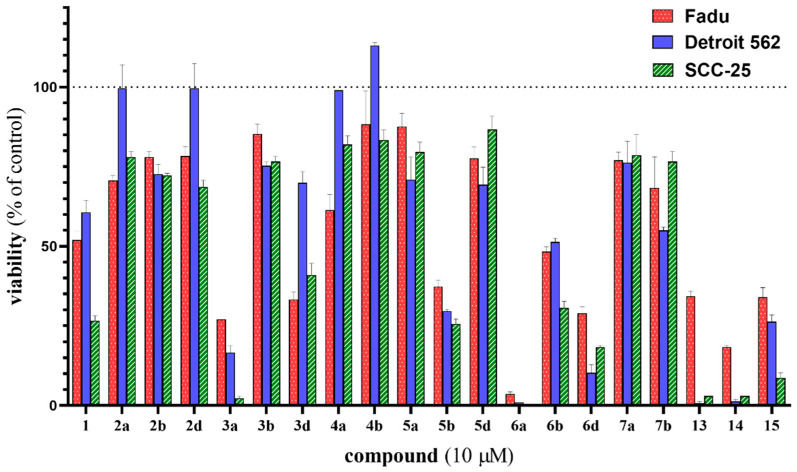
In vitro cell viability inhibition screening of the synthesized hybrid compounds on Fadu, Detroit 562 and SCC-25 NHSCC cell lines using CellTiter-Glo cell viability assay. Cells were treated for 72 h at 10 µM. The most effective **6a**, **13** and **14** hybrid molecules were selected for further investigation. Erlotinib (**1**) and chalcone (**15**) as reference fragments were also assessed. Data are presented as mean ± SD; *n* = 3.

The synthesized compounds were screened for their anticancer activities against three HNSCC cell lines (Fadu, Detroit 562 and SCC-25) using the CellTiter-Glo luminescent cell viability assay (Figure 2). The screening was performed at a 10 μM concentration and 72 h treatment time, and untreated cells (cultured in 0.2% DMSO-containing medium) were used as reference. The efficacy of the compounds that constitute the two fundamental moieties of our molecular hybrids, i.e., **1** and **15,** were also assessed. Although **3a**, **5b**, **6d** and **15** were also found to be more potent antiproliferative agents than **1**, among the tested hybrid molecules, **6a, 13** and **14** showed the highest efficacy (Figure 1 and Figure 2). These compounds proved to be significantly more potent than either **1** or **15** and were selected for further investigation.

### 2.3. Time- and Dose-Dependent Effects of Selected Hybrid Molecules on the Viability of HNSCC Cells

**Figure 3:**
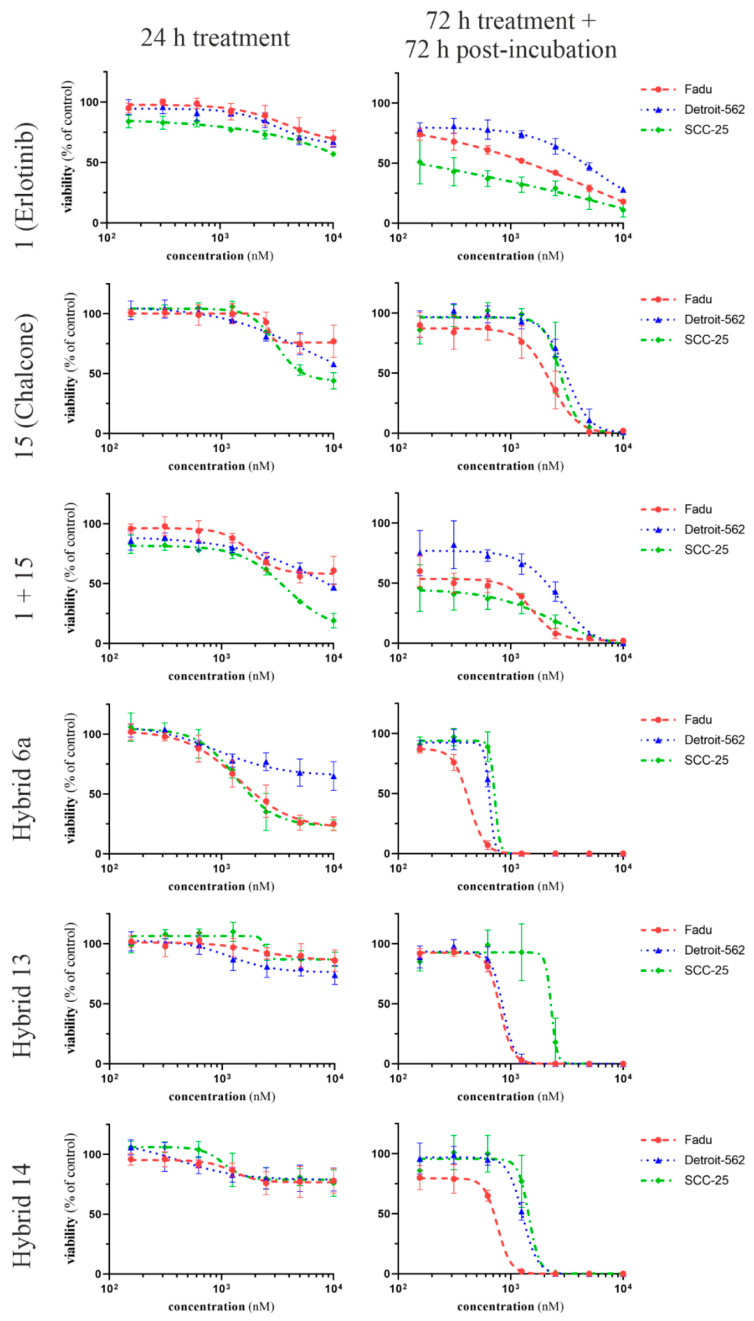
Dose- and time-dependent effects of the selected hybrid molecules on the viability of Fadu, Detroit 562 and SCC-25 cells. Hybrids (**6a**, **13** and **14**) and the reference compounds (**1**: erlotinib, **15**: chalcone and their combination in 1:1 ratio) were applied for 24 h or 72 h followed by 72 h postincubation period. Cell viability was determined by CellTiter-Glo assay. Curves were fitted by GraphPad Prism 8 software (nonlinear regression, variable slope; *n* = 4).

The viability inhibition potency of the selected hybrid molecules (**6a**, **13**, **14**) was further investigated on HNSCC cells using the CellTiter-Glo cell viability assay (Figure 3). Molecular fragments of the hybrids (**1** and **15**) as well as their combination (1:1 mixture) were tested as reference models.

Erlotinib significantly inhibited the viability of all tested cancer cells in the nanomolar range; however, it was far from being able to completely eradicate the cancer cells even at the highest concentration applied (10 µM). On the other hand, the effect of **15** (reference chalcone) was only detectable in the micromolar range. It must be pointed out as a novel finding that the most prominent hybrids (**6a**, **13** and **14**), showed superior potency not only to **1** and **15** when applied as single agents but also to the 1:1 combination of these reference compounds.

Among the hybrids, **6a** showed the highest efficiency both in the short term (24 h treatment) and long term (72 h treatment + 72 h postincubation). The results of the short-term treatment suggest that hybrids **13** and **14** have no direct cytotoxic effect, while **6a** showed significant viability inhibition on Fadu and SCC25 cells; however, it proved to be less efficient against Detroit 562 cells.

**Figure 4:**
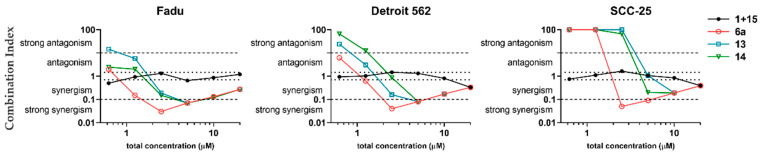
Concentration-dependent visualization of combination index of hybrids (**6a**, **13** and **14**) and their fragments: erlotinib (**1**) and chalcone (**15**) in 1:1 mixture. Combination index was calculated by CompuSyn using the mean values of cell viability data measured by CellTiter-Glo after 72 h treatment and 72 h postincubation. Total concentration means the overall concentration of **1** and **15** either in combination or in hybridized form.

The long-term treatment revealed the outstanding potency of the hybrids superior to that produced by their molecular components (**1** and **15**) either as single agents or in combination. According to the cell viability measurement, all three HNSCC cell lines were eradicated by the selected hybrids in the micromolar range. The IC_50_ values (Table 1) as well as the combination indexes (Figure 4) demonstrate that Fadu cells are the most sensitive and SCC-25 are the most resistant to the hybrids in our experiments. Dose-dependent combination indexes also reveal that the combination of **1** + **15** has only an additive effect, while each of the three hybrids exhibits strong synergism on all of the three HNSCC cell lines in the micromolar range. On the other hand, combination indexes of the hybrids indicate antagonism in the nanomolar range, which can be reasoned by the fact that the hybrids proved to be less effective in the nanomolar range compared to **1**.

### 2.4. Colony Formation Assay

A colony formation assay was performed in order to verify the elimination of HNSCC cells even after a single-dose treatment with hybrid molecules **6a**, **13** and **14**. Therefore, cells were treated only for 24 h at a 2.5 µM concentration followed by a 7-day-long postincubation period in complete medium. Untreated cells were used as control, and **1** and **15** served as references.

**Figure 5:**
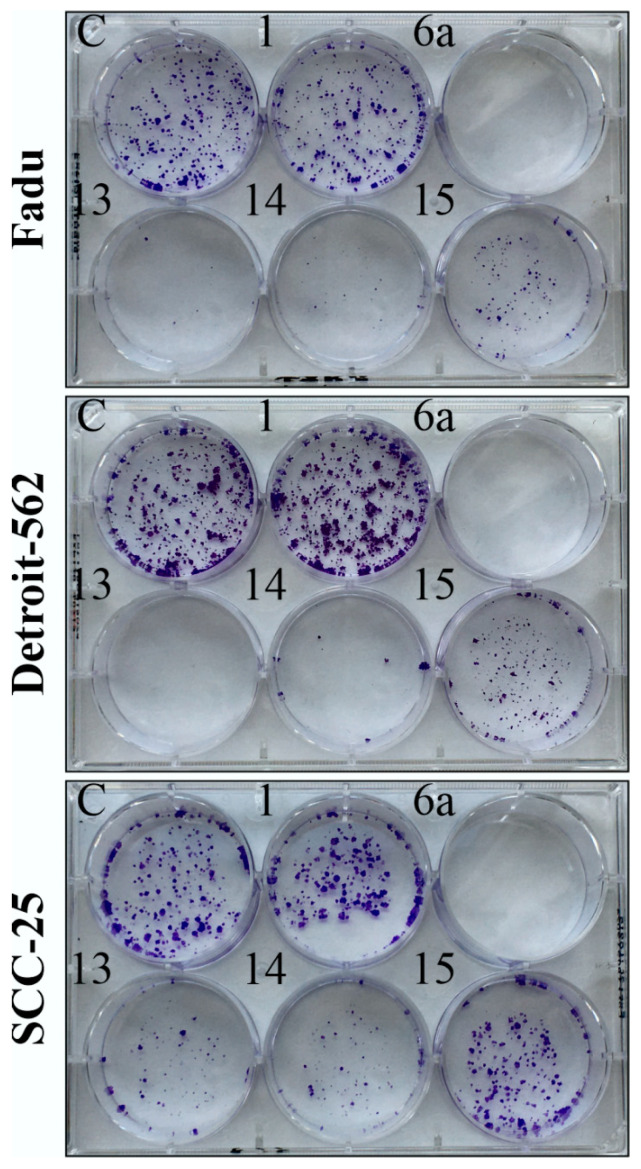
Colony formation assay of HNSCC cells using 2.5 uM concentration, 24 h treatment time and 1-week-long postincubation in treatment-free cell culture medium. **C**: control (medium containing 0.2% DMSO); **1**: erlotinib; **6a**, **13** and **14**: hybrids; **15**: chalcone.

In this experimental setup, erlotinib has no significant anticancer effect as it is demonstrated in Figure 5. Chalcone **15** proved to be more effective against Detroit 562 and Fadu cells than SCC-25 cells; however, it was far from being able to eliminate these cancer cells. Triazole-tethered hybrid **6a** was identified as the sole investigated compound able to completely eradicate the whole cell population of all three HNSSC cell lines tested, while **13** caused total lethality on Detroit 562 cells and proved to be highly efficient on Fadu cells as well. Surviving cancer cells were visible on each investigated cell line after the treatment with hybrid **14**. Since this compound proved to be the less effective one among the three hybrids in the colony formation assay, it has been excluded from further experiments.

### 2.5. Apoptosis and Necrosis Quantitation Assay

**Figure 6:**
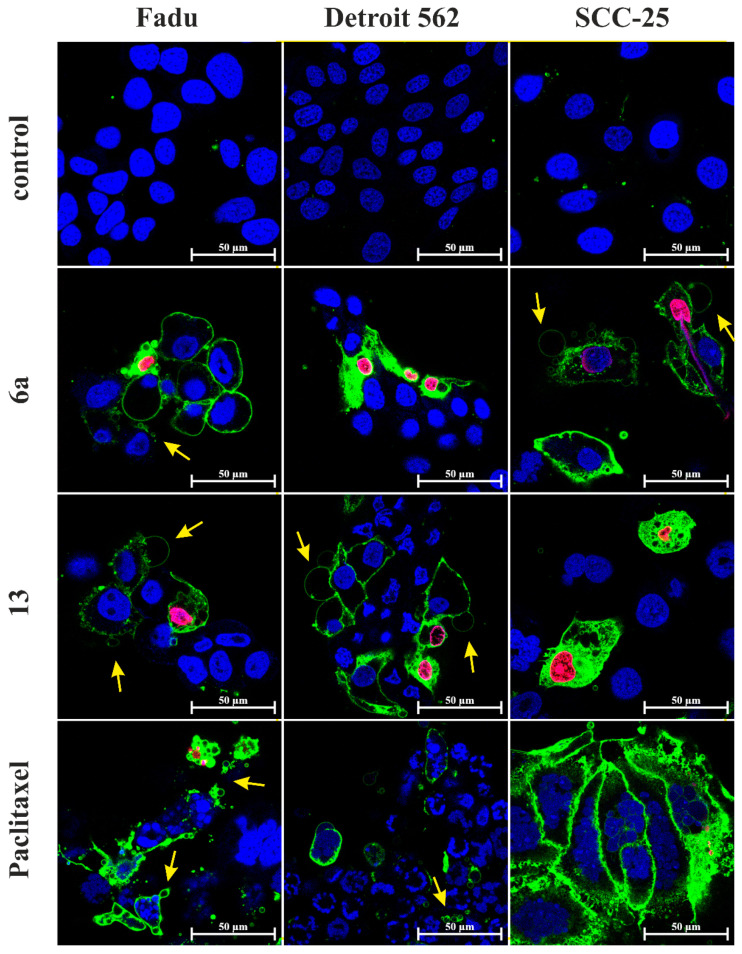
Apoptosis-necrosis detection assay on Fadu, Detroit 562 and SCC-25 HNSCC cells by confocal laser scanning microscopy. The cells were treated for 28 h at 2.5 µM. Paclitaxel was used as a positive control and untreated cells as a negative control. Nuclei were visualized by membrane-permeable dye DRAQ5 (blue). CF488A-labeled Annexin V stains apoptotic cells with green fluorescence. Membrane-impermeable Ethidium Homodimer III stains the nuclei of necrotic cells and late apoptotic cells exclusively (pink color). Plasma membrane blebs are marked with yellow arrows.

The mechanism of action of the most potent hybrids, **6a** and **13,** was investigated by an apoptosis-necrosis detection assay on the HNSCC cells. Paclitaxel, the emblematic apoptosis-inducing agent used in current HNSCC therapy, was employed as a positive control for apoptosis, and untreated cells served as a negative control. Nuclei were labeled with DRAQ5 (blue). Early apoptosis was defined here as annexin V-positive (green color) and EthD-III (pink color)-negative cells, while late apoptosis/necrosis was defined as both annexin V- and EthD-III-positive cells. As shown in Figure 6, hybrid **6a** resulted in the formation of several early apoptotic Fadu and SCC-25 cells but only late apoptotic/necrotic Detrotic-562 cells. In the case of treatment with **13**, early apoptosis was detectable in several Fadu and Detroit 562 cells but not in SCC-25 cells. Plasma membrane blebbing, as another potential morphological marker of apoptosis [45,46], was also revealed in some Annexin V-positive cells.

### 2.6. Mitochondrial Membrane Potential Detection

**Figure 7:**
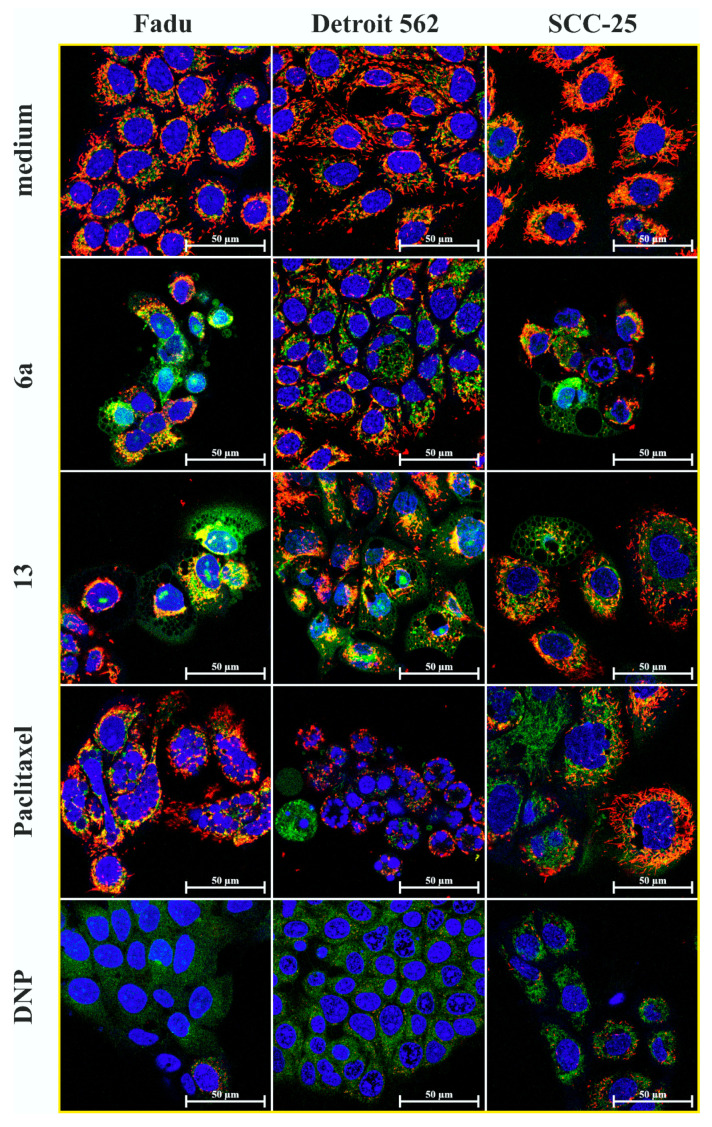
Mitochondrial Membrane Potential Using the Cationic JC-1 Dye. Nontreated cells were used as negative control, with their well-functioning mitochondria represented with red color. Mitochondrial uncoupling agent 2,4-Dinitrophenol (DNP) was used a positive control. Diffusive green color indicates the diminished mitochondrial membrane potential in DNP-treated cells. Hybrids **6a** and **13** resulted in significant mitochondrial dysfunction (at 2.5 µM after 28 h treatment) in HNSCC cells. Nuclei were visualized by membrane-permeable dye DRAQ5 (blue).

**Figure 8:**
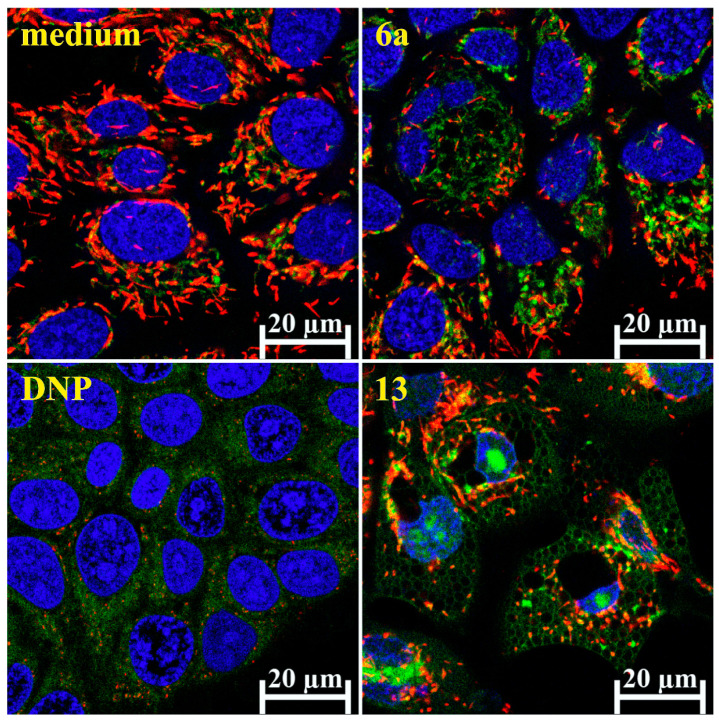
Morphological changes in Detroit 562 cells. Besides mitochondrial dysfunction (green color), JC-1 Dye (labels the well-functioning mitochondria with red color) revealed vacuolated cytoplasm and nuclear shrinkage in Detroit 562 cells, after 28 h of treatment with hybrid **13** at 2.5 µM. DNP was used as positive control for mitochondrial membrane depolarization. Nuclei were visualized by membrane-permeable dye DRAQ5 (blue).

Mitochondrial membrane potential as an indicator of mitochondrial function was investigated by the membrane-permeant JC-1 dye [47]. Mitochondrial uncoupling agent 2,4-Dinitrophenol (DNP) was used as a positive control [48]. Compared to the nontreated cells, where the well-functioning mitochondria appear with red color, both hybrids **6a** and **13** resulted in a significant reduction in membrane potential as indicated by the diffuse green color in Figure 7. Complementing the results of the Apoptosis and Necrosis Quantitation Assay, characteristic morphologic alterations were observed on the confocal images of JC-1-labeled cells as well. Vacuolated cytoplasm (bubble-like formation) was detectable in all three HNSCC cell lines after treatment with **13**. In contrast, vacuolated cytoplasm was not detectable in Detroit 562 cells treated by **6a** and only detectable in Fadu and SCC-25 cells, as shown in Figure 8. Upon treatment with **13,** nuclear shrinkage, as another morphological marker of programmed cell death [49], was also more pronounced than that caused by **6a** in Detroit 562 cells.

### 2.7. Real-Time Apoptosis Detection

**Figure 9:**
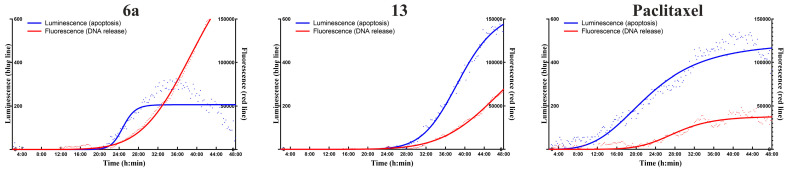
Real-time apoptosis detection in Detroit 562 cells. The cells were treated with the compounds at 2.5 µM and monitored for 48 h. DNS release as a marker of necrosis provided fluorescent signal (red curve), while annexin V binding as a marker of apoptosis resulted in luminescent signal (blue curve). Intensive increment of luminescent signal, which was followed a few hours later by the appearance of the fluorescent signal, refers to apoptosis. Paclitaxel was used as a positive control of apoptosis. While hybrid **6a** resulted in necrotic-like cell death, the effect of hybrid **13** proved to be more apoptotic-like.

The effects of hybrids **6a** and **13** were further characterized by using RealTime-Glo™ Annexin V Apoptosis and Necrosis Assay on Detroit 562 cells. In this experiment, DNS released as a marker of necrosis induced a fluorescent signal (red curve), while annexin V binding as a marker of apoptosis resulted in a luminescent signal (blue curve). Detroit 562 cells were monitored for 48 h in a CO_2_-incubated plate reader in the presence of the compounds. The intensive increment in the luminescent signal and subsequent appearance of the necrotic signal a few hours later refer to apoptosis, while the parallel increase in the two signals is the marker of necrosis. In correlation with the images obtained by the Apoptosis and Necrosis Quantitation Assay, the effect of **6a** proved to be rather necrotic in Detroit 562 cells, while the effect of **13** in character was in between apoptosis and necrosis when compared to paclitaxel, which served as a positive control of apoptosis (Figure 9).

## 3. Discussion

The main purpose of developing erlotinib–chalcone hybrids was to identify the first members of a novel class of anticancer small molecules as efficient drug candidates capable of overcoming erlotinib resistance in cancer therapy. The synthesized compounds were evaluated for their anticancer potential on three HNSCC cell lines, Fadu, Detroit 562 and SCC-25. The initial screening revealed an interesting structure-activity relationship regarding the dramatic difference observed in the effects of the triazole-tethered isomer pair **5a** and **6a**. While **5a** proved to be one of the least efficient hybrids, **6a** was identified as the most potent one. The only structural difference is the site (*ortho* vs. *para* position) of conjugation to the triazole linker on the phenol ring in the chalcone moiety. The strict “*ortho* vs. *para*” rule related to relative activity was not discernible in their analogs **13** and **14** containing the acetylenic linker, since both hybrids displayed similar activity against the investigated HNSCC cells. These structural differences highlight the pivotal role of the linker and the site of conjugation in building up the cancer-specific efficacy of these hybrids. According to the results of the screening, the three most prominent hybrids **6a**, **13** and **14** were selected for further investigation.

Time- and concentration-dependent cell viability measurements (based on quantitation of the ATP level) revealed that hybrids are significantly more effective than their molecular fragments (**1** and **15**), used either as single agents or in a 1:1 combination.

The short-term treatment (24 h) was expected to exclude the unfavorable cytotoxic effect of the hybrids. As it is shown in Figure 3, none of the tested compounds were able to reduce the viability of the cells to zero even at 10 µM, the highest concentration used. However, hybrid **6a** resulted in robust viability inhibition exclusively on Fadu and SCC-25 cells after 24 h, while Detroit 562 cells remained less affected. One possible explanation for the enhanced activity on Fadu and SCC-25 is the significant oxidative stress induced in these cells by **6a** containing the triazole linker. The oxidative effect was significantly lower in the case of the treatments with acetylene-tethered hybrids **13** and **14** as indicated by the results obtained by the Glutathione assay (SM: Figure S3 in Section S4.4), which was used for the investigation of oxidative stress.

The long-term treatment (72 h) followed by 72 h of postincubation was aimed to assess the survival of the cancer cells, which is the main cause of the early development of drug resistance. The long-term viability inhibition assay revealed a highly promising overwhelming efficacy of the studied hybrids, which is superior to that produced by their fragments. The hybrids were exclusively able to diminish the viability of HNSCC cells employed in the lower micromolar range. The IC_50_ values (Table 1) demonstrate that Fadu cells are the most sensitive against the hybrids, while SCC-25 cells are the most tolerant. Among the hybrids, **6a** proved to be the most potent one on all three cell lines. However, it is important to mention that the IC_50_ value characterizing hybrid **13** was found to be very close to that measured for **6a** on Detroit 562 cells.

It is well known that persistent cancer cells are prominently responsible for the early development of drug resistance, treatment failure and recurrent disease [50,51]. Since the capability of overcoming resistance in HNSCC cells was regarded as the most important effectiveness criterion for the hybrids, we performed a colony formation assay to assess their potency in the eradication of cancer cells even after an exposure of 24 h at 2.5 µM, serving as a model experiment to mimic a single-dose treatment in vitro. In this experimental setup, **1** and **15** were not able to significantly reduce the number of cancer cell colonies. Among the selected hybrids, only **6a** was able to totally eradicate all three cancer cells, while **13** abolished Detroit 562 cells totally and Fadu cells almost completely. Since hybrid **14** proved to be less effective than **6a** and **13,** it has been excluded from further experiments.

In search of a plausible reason for the effectual surplus of the two most prominent hybrids **6a** and **13,** we undertook further investigation by focusing on the potential targets selected on the basis of the following considerations related to documented mechanisms of action of the incorporated molecular fragments. Erlotinib, as the common structural element of hybrids, is a well-known inhibitor of EGFR [52]. In agreement with clinical experiences, our results also underline that the inhibition of EGFR might be able to reduce the proliferation rate of tumor cells to a limited extent, but this effect is not sufficient to eliminate them completely. Western blot analysis revealed that the superior anticancer effect of hybrids **6a** and **13** is independent of EGFR, since these hybrids were not able to inhibit EGFR even at 5 uM, much above their IC_50_ values. Data from the Western blot analysis can be found in the SM (Figure S1 in Section S4.2).

In the case of chalcones, several potential molecular targets have been previously identified as it was detailed in the Introduction. One of their most relevant mechanisms of action is the inhibition of tubulin polymerization [13,53]. Accordingly, we also carried out a tubulin polymerization assay; however, this experiment revealed that, contrary to reference chalcone **15,** its hybrid derivatives **6a** and **13** have no significant impact on this cellular process in vitro. The results of the tubulin polymerization assay can be found in the SM (Figure S2 in Section S4.3). Cell cycle analysis was also performed on all three HNSCC cell lines; however, neither **15** nor the hybrids induced any significant changes after 18 h treatment. Only erlotinib (**1**) produced a remarkable increment in the G0/G1 phase and decrement in the S phase. The results of the cell cycle analysis can also be found in the SM (Figure S4 in Section S4.5). Based on the molecular target exploration, we concluded that the effectual surplus of our hybrids is independent of the canonical targets of their molecular precursors.

Thus, the following additional experiments briefly discussed in the following paragraphs were focused on attempting to collect further information on such potential cell death mechanisms that might be responsible for the superior anticancer efficacy of the hybrids.

Upon treatment with **6a**, confocal microscopic images—generated by the Apoptosis and Necrosis Quantitation Assay—indicated apoptosis in Fadu and SCC-25 cells and marked necrotic cell death in Detroit 562 cells. In the case of treatment with **13,** a sign of apoptosis was detectable in Fadu and Detoit 562 cells, but apoptosis was less typical in SCC-25 cells (Figure 5).

Lysosomes also have an important role in cell death pathways. Triggered by lysosomal membrane permeabilization (LMP) [54], lysosomal cell death usually remains functional even in apoptosis-resistant cancer cells. The potential effect of the hybrids on the lysosomes was investigated by a Galectin-3 puncta assay, which is a reliable tool to detect LMP [55]. Hybrids were not able to induce LMP in HNSCC cells according to the Galectin-3 puncta assay as demonstrated by confocal images outlined in the SM (Figure S5 in Section S4.6).

Mitochondria have a pivotal role in intrinsic apoptosis and are also implicated in other forms of regulated cell death, including necroptosis, ferroptosis and pyroptosis [56]. Since mitochondrial outer membrane permeabilization (MOMP) is a crucial event for most apoptotic pathways, mitochondrial membrane potential is a frequently investigated marker in correlation with MOMP [57]. A JC-1-dye-based mitochondrial potential assay confirmed that the membrane potential of mitochondria was significantly decreased in hybrid-treated cells. Besides mitochondrial dysfunction, confocal microscopic images also disclosed striking morphological changes in these cells. Besides the enlarged membrane blebs detected in several Annexin V-positive cells, JC-1 dye revealed vacuolated cytoplasm in the treated cells as well. More interestingly, the investigated hybrids showed different specificity towards each cell type. In correlation with the previous experiments, the real-time apoptosis-necrosis assay confirmed that hybrid **13** induces more apoptosis-like cell death than **6a** in Detroit 562 cells.

## 4. Materials and Methods

### 4.1. Cell Culturing

Fadu (human pharyngeal carcinoma), Detroit 562 (human pharyngeal carcinoma) and SCC-25 (human tongue carcinoma) cell lines were obtained from American Type Culture Collection (ATCC, Rockville, MD, USA). Cell lines were cultured according to the instructions provided by ATCC. The authentication of the cell lines was validated by STR DNA analysis (Eurofins Scientific, Luxemburg). All cell lines were routinely screened for the absence of mycoplasma infection (DAPI staining).

### 4.2. CellTiter-Glo Cell Viability Assay

Fadu, Detroit 562 and SCC-25 head and neck cancer cells were seeded at 1000 cell/well onto a flat bottom white 96-well plate (BRANDplates^®^, cat. no.: 781965). After 48 h, cells were treated for the desired time and concentration. In the initial screening assay, compounds were applied at 10 µM for 72 h. In dose-dependent experiments, 2-fold serial dilution was used (from 10 µM to 156 nM). In the first case of dose-dependent assays, a 24 h long treatment was used. In the second case, 72 h of treatment was applied, which was followed by a 72 h long postincubation in treatment-free medium. The viability of the cells was measured by the CellTiter-Glo^®^ luminescent cell viability assay (Promega, Madison, WI, USA) according to the manufacturer’s instructions. The luminescence signal was recorded using a microplate reader (BioTek Synergy 2 Multi-Mode Reader, BioTek, Winooski, VT, USA). Dose–response curves (using a nonlinear regression model) were generated and IC_50_ values were calculated by GraphPad Prism 8 software. The combination index was calculated by CompuSyn software using the mean values of cell viability data measured by CellTiter-Glo after 72 h of treatment and 72 h of postincubation.

### 4.3. Colony Formation Assay

The long-term survival of cancer cells after treatment was investigated by a clonogenic assay. Cells were seeded in a transparent 6-well cell culture plate (VWR) (density: 750 cells/well). After seeding, cells were incubated for 72 h before the treatment. Cells were treated with the compounds at 2.5 µM for 24 h. After the treatment, the medium was removed, and the cells were washed with phosphate-buffered saline (PBS). Cells were further incubated in their corresponding medium for 7 days. Thereafter, the medium was removed and cells were washed with PBS and fixed with 4% paraformaldehyde for 10 min. After fixation, cells were washed with PBS, and 0.5% *w*/*v* crystal violet (CV) solution was added. After 1 h, the CV solution was removed, and cells were washed thoroughly with water. Images were created by Corel Photo-Paint 2019.

### 4.4. Apoptosis and Necrosis Quantitation Assay

Fadu, Detroit 562 and SCC-25 head cells were seeded in eight-well Ibidi^®^ μ-Slide microscopic slides (2 × 10^3^ cells/well) and allowed to adhere for 48 h. Cells were then treated at a 2.5 µM concentration in cell culture medium supplemented with 10% FBS and incubated in a humidified, 5% CO_2_ atmosphere incubator for 28 h at 37 °C. Apoptosis and Necrosis Quantitation Kit Plus (Biotium, cat. no.: 30065) was used according to the manufacturer’s instructions. For nuclear staining of live cells, DRAQ5 was used (5 µM, 30 min). Images of cells were acquired with a confocal laser microscope (Zeiss Confocal LSM 710, Carl Zeiss AG, Oberkochen, Germany). (Objective: Plan-Apochromat 63×/1.40 Oil DIC M27. Pinhole: 0.99 AU. Laser wavelength: 488 nm and 633 nm. Detection wavelength: 504–536 nm; 602–631 nm; and 692–758 nm).

### 4.5. Mitochondrial Membrane Potential Detection

Fadu, Detroit 562 and SCC-25 head cells were seeded in eight-well Ibidi^®^ μ-Slide microscopic slides (2 × 10^3^ cells/well) and allowed to adhere for 48 h. Cells were then treated (6a, 13 and paclitaxel) at a 2.5 µM concentration in cell culture medium supplemented with 10% FBS and incubated in a humidified, 5% CO_2_ atmosphere incubator for 28 h at 37 °C. 2,4-Dinitrophenol was added to nontreated cells only 1 h before the JC-1 dye. After the treatment, JC-1 mitochondrial dye (5 µg/mL) and DRAQ-5 nuclear dye (5 µM) were added to the cells. The cells were incubated in a 5% CO_2_ atmosphere incubator for 30 min at 37 °C. Images of cells were acquired with a confocal laser microscope (Zeiss Confocal LSM 710, Carl Zeiss AG, Oberkochen, Germany). (Objective: Plan-Apochromat 63×/1.40 Oil DIC M27. Pinhole: 1.01 AU. Laser wavelength: 488 nm and 633 nm. Detection wavelength: 520–540 nm; 583–602 nm; and 683–758 nm).

### 4.6. Real-Time Apoptosis Detection

Detroit 562 cells were seeded at 1000 cell/well onto a black, half-area, and clear-bottom 96-well plate. After 48 h of adherence, cells were treated for the compounds at 2.5 µM. Reagents of the RealTime-Glo™ Annexin V Apoptosis and Necrosis Assay were added according to the manufacturer’s instructions (https://worldwide.promega.com (accessed on 1 October 2021)). Luminescent and fluorescent signals were detected every 5 min during the 48 h long treatment by a Clariostar Multi-Mode Plate Reader (BMG LABTECH). The plate reader maintained the 5% CO_2_ atmosphere and 37 °C during the measurement. Data were evaluated by MS Excel and GraphPad Prism 8 software.

## 5. Conclusions

The development of hybrids was aimed to enhance the anticancer efficacy of erlotinib (**1**) and some representative chalcones displayed as single agents by their incorporation into hybrids with potential multitarget character containing acetylenic and triazole linkers between these pharmacophoric molecular fragments. Thus, the novel hybrids were accessed by copper(I)-catalyzed azide-alkyne [2 + 3] cycloadditions and Sonogashira coupling reactions followed by standard Claisen–Smith condensation. Since tumor heterogeneity and, consequently, drug resistance in HNSCC represent a great unmet medical need for more efficient drug therapies, the novel hybrids were tested in three HNSCC cell lines Fadu, Detroit 562 and SCC-25. The investigation of the hybrids was focused on such features of their anticancer potential, which may be manifested in their pronounced ability to overcome resistance in these cancers.

A screening assay, followed by time- and dose-dependent cell viability measurements, demonstrated that the most prominent hybrids (**6a**, **13** and **14**) have an efficacy superior to their molecular fragments erlotinib (**1**) and reference chalcone (**15**) and revealed specific structure–activity relationships. These hybrids showed very strong synergism in the low micromolar range in all three HNSCC cell lines. Hybrid **6a** resulted in the total eradication of all the investigated cancer cells at 2.5 µM, while **13** also proved to be markedly efficient against Fadu and Detroit 562 cells. Experiments focusing on the mechanism of action indicated that the enhanced efficacy of the most potent hybrids is independent of the canonical molecular targets of their precursors, pointing to the need for further explorations directed to disclose the cause of their prominent efficacy. On the other hand, phosphatidylserine exposure, a decrease in mitochondrial membrane potential cytoplasmic vacuolization and nuclear shrinkage are typical markers of programmed cell death, which were observed in hybrid-treated HNSCC cells. Further experiments supported that the effect of **13** might be related to necroptosis in Detroit 562 cells. Although the exact mechanism of action of these hybrids remains unclear, their prominent anticancer efficacy demonstrated in the experiments justifies a highly promising therapeutic potential, which warrants further investigation. Finally, the expected new biological results with particular regard to the identification of cellular target(s) and mechanism of action might be taken into account in a rational design and synthesis of further hybrids with enhanced anticancer activity explorable in the therapy of HNSCC-related diseases.

## Figures and Tables

**Table 1:** IC_50_ values of hybrids (**6a**, **13** and **14)** and reference fragments **(1** and **15)** as single agents and in 1:1 combination, after 72 h treatment and 72 h postincubation. IC_50_ values were determined by GraphPad Prism 8 software (nonlinear regression, variable slope, best fit values are presented, *n* = 4).

IC_50_ Values (nM)						
	1	15	1 + 15	6a	13	14
Fadu	1199	1880	362	389	770	658
Detroit 562	4035	3074	1488	673	810	1264
SCC-25	180	2777	173	725	1935	1444

## Data Availability

The data generated and analyzed during our research are not available in any public database or repository but will be shared by the corresponding authors upon reasonable request.

## Supplementary Materials

The following are available online at https://www.mdpi.com/article/10.3390/ijms24043456/s1.
