# Supplementary material for: Novel Erlotinib–Chalcone Hybrids Diminish Resistance in Head and Neck Cancer by Inducing Multiple Cell Death Mechanisms
Source: Int J Mol Sci. 2023 Feb 9;24(4):3456. doi: 10.3390/ijms24043456 (PMC9964293; doi:10.3390/ijms24043456)
Supplement: Supplementary file 1 [file ijms-24-03456-s001.zip › ijms-2176280-supplementary.pdf]

Novel Erlotinib–Chalcone Hybrids Diminish Resistance in Head and Neck Cancer by Inducing Multiple Cell Death Mechanisms

József Murányi [1], Cintia Duró [2], Bianka Gurbi [1], István Móra [1], Attila Varga [1], Krisztina Németh [3], József Simon [4], Miklós Csala [1,*] and Antal Csámpai [2,*]

[1] Department of Molecular Biology, Semmelweis University, Tűzoltó u. 37-47, H-1094 Budapest, Hungary
[2] Department of Organic Chemistry, Eötvös Loránd University (ELTE), Pázmány P. Sétány 1/A, H-1117 Budapest, Hungary
[3] MS Metabolomics Research Group, Centre for Structural Study, Research Centre for Natural Sciences, Eötvös Loránd Research Network, Magyar Tudósok Krt. 2.
[4] Research Group of Analytical Chemistry, University of Pannonia, Egyetem utca 10, H-8200 Veszprém, Hungary
* Correspondence: csala.miklos@med.semmelweis-univ.hu (M.C.); antal.csampai@ttk.elte.hu (A.C.)

Supplementary Materials

Table of content

1 Chemical synthesis 3

1.1 General information 3

1.2 General procedures and characterization data 3

1.2.1 Synthesis of novel chalcones used as reagents 3

1.2.2 General Synthesis of Erlotinib Hybrids 7

2 Copies of NMR spectra 15

2.1 NMR spectra of novel chalcone analogues 15

2.2 NMR spectra of novel Erlotinib hybrids with 1,4-disubstituted triazole linkers 21

2.3 NMR spectra of novel Erlotinib hybrids with 1,4-disubstituted alkyne linkers 29

3 Copies of the HRMS spectra 33

3.1 HRMS spectra of novel chalcone analogues 33

3.2 HRMS spectra of novel Erlotinib hybrids with 1,4-disubstituted triazole linkers 36

3.3 HRMS spectra of novel Erlotinib hybrids with alkyne linkers 40

4 Biological evaluation 42

4.1 Celltiter-Glo Cell Viability Assay Data 42

4.2 EGFR inhibition efficacy of compounds on Fadu, Detroit-562 and SCC-25 cells 44

4.3 Inhibition of tubulin polymerization 45

4.4 Glutathione assay 46

4.5 Cell cycle analysis 46

4.6 Galectin-3 puncta assay 48

1 Chemical synthesis

1.1 General information

The measured HRMS, ^{1}H- and ^{13}C-NMR data of the novel chalcone derivatives and hybrid compounds are consistent with their structure, but the following remarks are necessary to make. The presence of the 1,4-disubstituted triazole ring in **5a**,**b**,**d** and **7a**,**b** featuring *para*-disubstitution in their central chalcone region is confirmed by the highly characteristic chemical shift range 9.37–9.47 ppm in which the H8′ signal is discernible in spectra registered of these hybrids, On the other hand, due to the anisotropic shielding effect of the proximal C=C bond, this diagnostic signal is upfield-shifted to 9.00–9.02 ppm in the ^{1}H-NMR spectra of hybrids **6a**, **6b** and **6d** featuring *ortho*-disubstitution in the central chalcone region. Providing further support for the structures of **5a**,**b**,**d**, **6a, 6b, 6d** and **7a**,**b,** in their ^{13}C-NMR spectra the C7′- and C8′-resonances can be found in the typical chemical shift ranges 147.3–148.4 ppm and 120.0–124.5 ppm, respectively, characteristic for the triazole linker unit. On the other hand, the structures of **13** and **14** gain support from C7′- and C8′-signals of the alkyne linker discernible in the typical chemical shift ranges 92.0–96.1 ppm and 85.6–89.6 ppm, respectively. (The numbering of atoms can be found on the structures presented along with the detailed characterization of the compounds.)

All manipulations with chemicals, synthesized compounds and other substances were performed under laboratory fume hoods, wearing rubber gloves. All fine chemicals were obtained from commercially available sources ((Merck (Budapest, Hungary), Fluorochem (Hadfield, UK.), Molar Chemicals (Halásztelek, Hungary), VWR (Debrecen, Hungary)) and used without further purification. Dimethylformamide was distilled from calcium hydride. Merck Kieselgel (230–400 mesh, 60 Å) was used for flash column chromatography. The ^{1}H- and ^{13}C-NMR spectra of all compounds were recorded in $CDCl_3$ or DMSO-d_6 solution in 5 mm tubes at RT, on a Bruker DRX-500 spectrometer at 500.13 (^{1}H) and 125.76 (^{13}C) MHz, and Avance NEO 400 spectrometer at 400.16 (^{1}H) and 100.62 (^{13}C) MHz, with the deuterium signal of the solvent as the lock and TMS as the internal standard. The HSQC, HMBC and COSY spectra, which support the exact assignments the of ^{1}H- and ^{13}C- NMR signals, were obtained by using the standard Bruker pulse programs. For each compound characterized in this session the numbering of atoms used for assignment of ^{1}H- and ^{13}C-NMR signals do not correspond to IUPAC rules reflected from the given systematic names. High resolution measurements were performed on a Sciex TripleTOF 5600+ high resolution tandem mass spectrometer equipped with DuoSpray ion source. Electrospray ionization was applied in positive ion detection mode. Samples were dissolved in acetonitrile and flow injected into acetonitrile:water 50:50 flow. The flow rate was 0.2 mL/min. The resolution of the mass spectrometer was 35000.

1.2 General procedures and characterization data

1.2.1 Synthesis of novel chalcones used as reagents

Tert-butyl (E)-(4-(3-(4-azidophenyl)acryloyl)phenyl)carbamate (**2c**)

4-Azidobenzaldehyde (0.74 g, 5.0 mmol, 1.0 eq.) and *tert*-butyl (4-acetylphenyl)carbamate (1.18 g, 5.0 mmol, 1.0 eq.) were dissolved in EtOH (15 mL), after about 5 minutes of stirring at room temperature 10% solution of NaOH (2 mL) was added. The resulting reaction mixture was stirred for 12 h at room temperature in darkness, then diluted with water (50 mL), and extracted with DCM (3 x 50 mL). The combined organic phase was washed with saturated NaCl solution, dried on Na_2SO_4 and evaporated to dryness. The analytical sample was recrystallized from hexane. Purity is 75% determined by ^{1}H-NMR spectra, impurity is *tert*-butyl (*E*)-(4-(3-(4-nitrophenyl)acryloyl)phenyl)carbamate, which proved to be inseparable from.

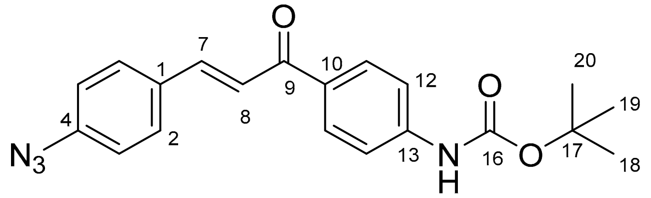

Scheme 1. Structure of **2c** with the numbering of atoms used for the assignment of NMR data.

Yellow solid; Yield: 1.39 g (76%); ^{1}H-NMR ($CDCl_3$): 7.96 (d, *J* = 8.7 Hz, 2H, H11, H15), 7.73 (d, *J* = 15.5 Hz, 1H, H8), 7.60 (d, *J* = 8.3 Hz, 2H, H2, H6), 7.53-7.42 (m, 3H, H7, H12, H14), 7.03 (d, *J* = 8.3 Hz, 2H, H3, H5), 6.73 (s, 1H, N<u>H</u>), 1.51 (s, 9H, H18, H19, H20); ^{13}C-NMR ($CDCl_3$): 188.6 (C9), 152.2 (C16), 142.8 (C13), 142.0 (C4), 141.0 (C7), 132.7 (C10), 130.1 (C2, C6), 129.9 (C11, C15), 125.6 (C1), 121.3 (C8), 119.5 (C3, C5), 117.6 (C12, C14), 81.4 (C17), 28.3 (C18, C19, C20); HRMS exact mass calcd. for $C_{20}H_{21}N_4O_3$ $[M+H]^+$, requires m/z: 365.1614; found m/z: 365.1612.

Tert-butyl (E)-(4-(3-(4-nitrophenyl)acryloyl)phenyl)carbamate

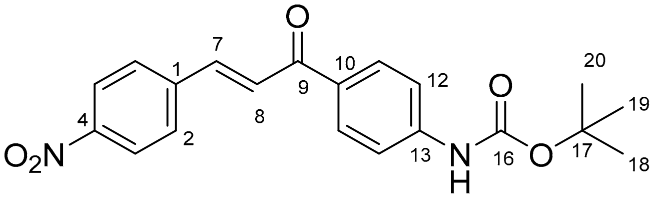

Scheme 2. Structure of *tert*-butyl (*E*)-(4-(3-(4-nitrophenyl)acryloyl)phenyl)carbamate with the numbering of atoms used for the assignment of NMR data.

^{1}H-NMR ($CDCl_3$): 8.24 (d, *J* = 8.3 Hz, 2H, H3, H5), 7.99 (d, *J* = 8.7 Hz, 2H, H11, H15), 7.77 (d, *J* = 15.5 Hz, 1H, H8), 7.75 (d, *J* = 8.3 Hz, 2H, H2, H6), 7.61 (m, 1H, H7), 7.53-7.42 (m, 2H, H12, H14), 6.77 (s, 1H, N<u>H</u>), 1.60 (s, 9H, H18, H19, H20); ^{13}C-NMR ($CDCl_3$): 187.9 (C9), 152.1 (C16), 148.4 (C4), 143.1 (C13), 142.9 (C7), 141.2 (C1), 133.0 (C10), 130.2 (C11, C15), 128.9 (C2, C6), 124.2 (C3, C5), 121.3 (C8), 117.6 (C12, C14), 81.5 (C17), 28.3 (C18, C19, C20); HRMS exact mass calcd. for $C_{20}H_{21}N_2O_5$ $[M+H]^+$, requires m/z: 369.1450; found m/z: 369.1451.

(E)-1-(4-aminophenyl)-3-(4-azidophenyl)prop-2-en-1-one (**2d**)

Azide **2c** (0.12 g, 0.32 mmol) was dissolved in MeCN (10 mL) and 2 M HCl (5 mL) was added to the solution. The resulting mixture was stirred for 12 h at room temperature, then diluted with water (20 mL) and carefully neutralized by the addition of solid Na_2CO_3 in small portions. The precipitated solid was separated by filtration and recrystallized from MeOH-water. Purity is 75% determined by ^{1}H-NMR spectra, impurity is (*E*)-1-(4-aminophenyl)-3-(4-nitrophenyl)prop-2-en-1-one, which proved to be inseparable from.

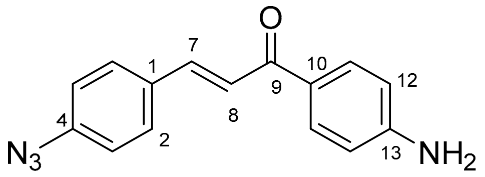

Scheme 3. Structure of **2d** with the numbering of atoms used for the assignment of NMR data.

Yellow solid; Yield: 37.6 mg (44%); ^{1}H-NMR (DMSO-*d*$_6$): 7.94-7.81 (m, 4H, H2, H3, H5, H6), 7.79 (d, *J* = 15.6 Hz, 1H, H8), 7.55 (d, *J* = 15.4 Hz, 1H, H7), 7.13 (d, *J* = 8.0 Hz, 2H, H11, H15), 6.57 (d, *J* = 8.0 Hz, 2H, H12, H14), 6.11 (s, 2H, N<u>H2</u>); ^{13}C-NMR (DMSO-d$_6$): 186.3 (C9), 154.3 (C13), 141.4 (C7), 139.1 (C4), 131.6 (C11, C15), 130.7 (C2, C6), 127.2 (C1), 125.8 (C10), 124.4 (C8), 120.0 (C3, C5), 113.3 (C12, C14); HRMS exact mass calcd. for $C_{15}H_{13}N_4O$ $[M+H]^+$, requires m/z: 265.1089; found m/z: 265.1082.

(E)-1-(4-aminophenyl)-3-(4-nitrophenyl)prop-2-en-1-one

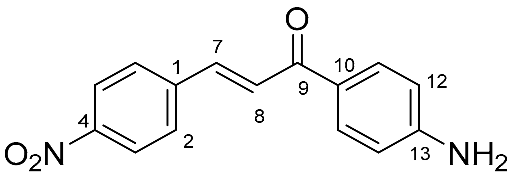

Scheme 4. Structure of (*E*)-1-(4-aminophenyl)-3-(4-nitrophenyl)prop-2-en-1-one with the numbering of atoms used for the assignment of NMR data.

^{1}H-NMR (DMSO-*d*$_6$): 8.22 (d, *J* = 8.3 Hz, 2H, H2, H6), 8.07 (d, *J* = 8.4 Hz, 2H, H3, H5), 8.02 (d, *J* = 15.5 Hz, 1H, H8), 7.90 (d, 2H, H11, H15, overlapped by H2, H3, H5, H6 from **3d**), 7.64 (d, *J* = 15.5 Hz, 1H, H7), 6.57 (2H, H12, H14, overlapped by H12, H14 from **3d**), 6.21 (s, 1H, N$\underline{H_2}$); ^{13}C-NMR (DMSO-*d*$_6$): 187.1 (C9), 154.7 (C13), 148.2 (C4), 141.3 (C7), 131.9 (C11, C15), 129.9 (C2, C6), 141.3 (C1), 125.4 (C10), 127.0 (C8), 122.4 (C3, C5), 115.5 (C12, C14); HRMS exact mass calcd. for $C_{15}H_{13}N_2O_3$ [M+H]$^+$, requires m/z: 269.0926, found m/z: 269.0921.

Tert-butyl (E)-(4-(3-(2-azidophenyl)acryloyl)phenyl)carbamate (**3c**)

2-Azidobenzaldehyde (0.74 g, 5.0 mmol, 1.0 eq.) and *tert*-butyl (4-acetylphenyl)carbamate (1.18 g, 5.0 mmol, 1.0 eq.) were dissolved in EtOH (15 mL), after about 5 minutes of stirring at room temperature powdered NaOH (0.2 g, 5.0 mmol, 1.0 eq.) was added. The resulting reaction mixture was stirred for 12 h at room temperature in darkness, then diluted with water (50 mL), and extracted with DCM (3 x 50 mL). The combined organic phase was washed with saturated NaCl solution, dried on Na_2SO_4 and evaporated to dryness. The residue was purified by flash chromatography on silica gel, using DCM. The obtained oil was crystallized by MeOH-water.

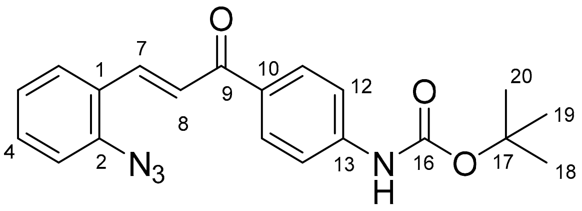

Scheme 5. Structure of **3c** with the numbering of atoms used for the assignment of NMR data.

Yellow solid; Yield: 0.58 g (32%); ^{1}H-NMR ($CDCl_3$): 8.01-7.88 (m, 3H, H7, H11, H15), 7.67 (d, *J* = 7.8 Hz, 1H, H6), 7.52 (d, *J* = 15.7 Hz, 1H, H8), 7.48 (d, *J* = 8.5 Hz, 2H, H12, H14), 7.40 (t, *J* = 7.8 Hz, 1H, H4), 7.19 (d, *J* = 8.1 Hz, 1H, H3), 7.15 (t, *J* = 7.6 Hz, 1H, H5), 6.86 (s, br, 1H, N$\underline{H}$), 1.50 (s, 9H, H18, H19, H20); ^{13}C-NMR ($CDCl_3$): 189.0 (C9), 152.2 (C16), 142.9 (C13), 139.6 (C2), 138.6 (C7), 132.5 (C10), 131.4 (C4), 130.2 (C11, C15), 128.5 (C6), 126.6 (C1), 124.9 (C5), 123.6 (C8), 118.9 (C3), 117.5 (C12, C14), 81.3 (C17), 28.3 (C18, C19, C20); HRMS exact mass calcd. for $C_{20}H_{21}N_4O_3$ [M+H]$^+$, requires m/z: 365.1614; found m/z: 365.1611.

(E)-1-(4-aminophenyl)-3-(2-azidophenyl)prop-2-en-1-one (**3d**)

Azid**e 3c** (0.10 g, 0.27 mmol) was dissolved in MeCN (10 mL) and 2 M HCl (5 mL) was added to the solution. The resulting mixture was stirred for 12 h at room temperature, then diluted with water (20 mL) and carefully neutralized by the addition of solid Na_2CO_3 in small portions. The precipitated solid was separated by filtration and recrystallized from MeOH-water.

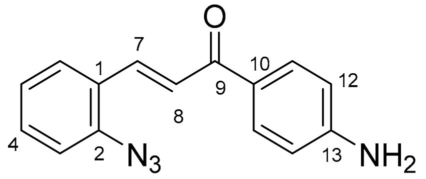

Scheme 6. Structure of **3d** with the numbering of atoms used for the assignment of NMR data.

Yellow solid; Yield: 34.8 mg (49%); ^{1}H-NMR (DMSO-d_6): 8.02 (d, J = 7.8 Hz, 1H, H6), 7.87 (d, J = 8.6 Hz, 2H, H11, H15), 7.81 (d, J = 15.7 Hz, 1H, H8), 7.76 (d, J = 15.7 Hz, 1H, H7), 7.46 (d, J = 7.8 Hz, 1H, H4), 7.33 (d, J = 7.8 Hz, 1H, H3), 7.21 (t, J = 7.8 Hz, 1H, H5), 6.57 (d, J = 8.6 Hz, 2H, H12, H14); ^{13}C-NMR (DMSO-d_6): 186.2 (C9), 154.3 (C13), 139.2 (C2), 135.4 (C7), 131.9 (C4), 131.6 (C11, C15), 128.5 (C6), 126.7 (C1), 125.7 (C10), 125.6 (C5), 124.2 (C8), 119.8 (C3), 113.3 (C12, C14); HRMS exact mass calcd. for $C_{15}H_{13}N_4O$ [M+H]$^+$, requires m/z: 265.1089, found m/z: 265.1079.

(E)-1-(4-azidophenyl)-3-(ferrocenyl)prop-2-en-1-one (**4b**)

Ferrocenecarboxaldehyde (1.07 g, 5.0 mmol, 1.0 eq.) and 4-azidoacetophenone (0.98 g, 5.0 mmol, 1.0 eq.) were dissolved in EtOH (15 mL), after about 5 minutes of stirring at room temperature powdered NaOH (0.2 g, 5.0 mmol, 1.0 eq.) was added. The resulting reaction mixture was stirred for 12 h at room temperature in darkness, then diluted with water (50 mL), and extracted with DCM (3 x 50 mL). The combined organic phase was washed with saturated NaCl solution, dried on Na_2SO_4 and evaporated to dryness through the solid product crystallized.

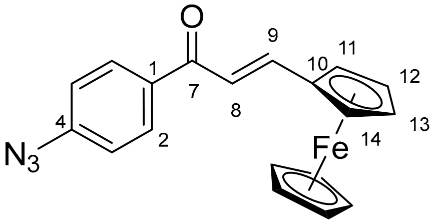

Scheme 7. Structure of **4b** with the numbering of atoms used for the assignment of NMR data.

Dark red solid; Yield: 1.26 g (70%); ^{1}H-NMR ($CDCl_3$): 7.98 (d, J = 8.1 Hz, 2H, H2, H6), 7.74 (d, J = 15.3 Hz, 1H, H9), 7.11-7.02 (m, 3H, H3, H5, H8), 4.57 (s, 2H, H11, H14), 4.46 (s, 2H, H12, H13), 4.15 (s, 5H, η^5-$C_5\underline{H_5}$); ^{13}C-NMR ($CDCl_3$): 188.0 (C7), 147.0 (C9), 144.2 (C4), 135.2 (C1), 130.3 (C2, C6), 119.0 (C8), 118.4 (C3, C5), 79.1 (C10), 71.5 (C12, C13), 69.8 (η^5-$\underline{C_5}H_5$), 69.1 (C11, C14); HRMS exact mass calcd. for $C_{19}H_{16}N_3OFe$ [M+H]$^+$, requires m/z: 358.0643, found m/z: 358.0635.

(E)-3-phenyl-1-(3,4,5-trimethoxyphenyl)prop-2-en-1-one (**15**)

Benzaldehyde (0.53 g, 5.0 mmol, 1.0 eq.) and 1-(3,4,5-trimethoxyphenyl)ethan-1-one (**12a**) (1.05 g, 5.0 mmol, 1.0 eq.) were dissolved in EtOH (15 mL), after about 5 minutes of stirring at room temperature 10% solution of NaOH (2 mL) was added. The resulting reaction mixture was stirred for 12 h at room temperature, then poured on water (50 mL). The obtained precipitated solid was separated by filtration and washed with water.

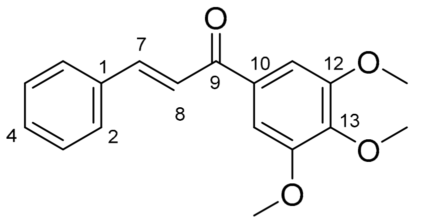

Scheme 8. Structure of **15** with the numbering of atoms used for the assignment of NMR data.

Light yellow solid; Yield: 0.96 g (64%); ^{1}H-NMR (DMSO-d_6): 8.00 (d, *J* = 15.6 Hz, 1H, H8), 7.93-7.88 (m, 2H, H3, H5), 7.76 (d, *J* = 15.7 Hz, 1H, H7), 7.52-7.41 (m, 3H, H2, H4, H6), 3.91 (s, 2H, C12OC$\underline{H}_3$, C14OC$\underline{H}_3$), 3.78 (s, 1H, C13OC$\underline{H}_3$); ^{13}C-NMR (DMSO-d_6): 188.4 (C9), 153.4 (C12, C14), 144.3 (C7), 142.5 (C13), 135.2 (C1), 133.4 (C10), 131.1 (C4), 129.5 (C3, C6), 129.3 (C2, C6), 122.4 (C8), 106.7 (C11, C15), 60.7 (C13O$\underline{C}H_3$), 56.7 (C12O$\underline{C}H_3$, C14O$\underline{C}H_3$); HRMS exact mass calcd. for $C_{18}H_{19}O_4$ [MH]+, requires m/z: 299.1283, found m/z: 299.1275.

1.2.2 General Synthesis of Erlotinib Hybrids

1.2.2.1 Synthesis of novel Erlotinib hybrids with 1,4-disubstitued triazole linker

Erlotinib (**1**) (0.39 g, 1.0 mmol, 1.0 eq.), the appropriate azidochalcone (**2a-c**, **4a,b** and **3a-c**) (1.0 mmol, 1.0 eq.) and CuI (38.0 mg, 0.2 mmol, 0.2 eq.) were dissolved in DMSO (4 mL). The resulting reaction mixture was stirred at room temperature for 12 h, diluted with water (20 mL), and extracted with DCM (3 x 25 mL). The combined organic phase was washed with saturated NaCl solution, dried on Na_2SO_4, and evaporated to dryness. The residue was purified by flash column chromatography on silica gel, using DCM:MeOH mixtures (the ratio of DCM and MeOH was varied from 60:1 to 5:1, and in some cases addition of 5% TEA was necessary). The analytical samples were crystallized by water, MeOH-water or Et_2O.

(E)-1-(4-(4-(3-((6,7-bis(2-methoxyethoxy)quinazolin-4-yl)amino)phenyl)-1H-1,2,3-triazol-1-yl)phenyl)-3-(3,4,5-trimethoxyphenyl)prop-2-en-1-one (**5a**)

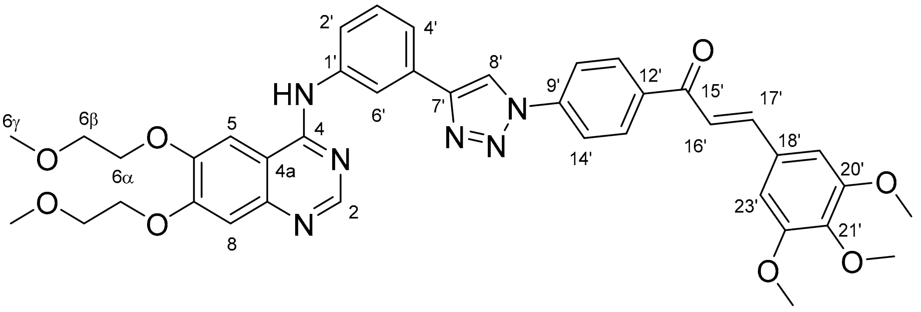

Scheme 9. Structure of **5a** with the numbering of atoms used for the assignment of NMR data.

Orange solid; Yield: 0.61 g (83%); ^{1}H-NMR (DMSO-d_6): 10.01 (s, br, 1H, N$\underline{H}$), 9.47 (s, 1H, H8′), 8.57 (s, 1H, H2), 8.38 (d, *J* = 8.4 Hz, 2H, H11′, H13′), 8.33 (t, *J* = 1.9 Hz, 1H, H6′), 8.16 (d, *J* = 8.4 Hz, 2H, H10′, H14′), 7.96 (s, 1H, H5), 7.92 (d, *J* = 15.6 Hz, 1H, H16′), 7.85 (dd, *J* = 7.6 Hz, 1.4 Hz, 1H, H2′), 7.76-7.68 (m, 2H, H4′, H17′), 7.53 (t, *J* = 7.8 Hz, 1H, H3′), 7.23 (s, 2H, H19′, H23′), 7.21 (s, 1H, H8), 4.32-4.24 (m, 4H, H6α H7α), 3.84 (s, 6H, C20′OC$\underline{H}_3$, C22′OC$\underline{H}_3$), 3.76 (t, *J* = 4.8 Hz, 2H, H6β), 3.74-3.70 (m, *J* = 4.8 Hz, 2H, H7β), 3.64 (s, 3H, C21′OC$\underline{H}_3$), 3.34 (s, 3H, H6γ), 3.32 (s, overlapped by HDO signal from solvent, 3H, H7γ); ^{13}C-NMR (DMSO-d_6): 188.4 (C15′), 157.5 (C4), 154.9 (C7), 153.6 (C20′, C22′), 152.1 (C2), 149.1 (C6), 147.9 (C7′), 145.6 (C1a, C17′), 140.5 (C21′), 140.0 (C9′), 139.8 (C1′), 137.8 (C12′), 131.0 (C11′, C13′), 130.9 (C5′), 130.6 (C18′), 129.8 (C3′), 122.1 (C4′), 123.7 (C2′), 121.5 (C16′), 120.4 (C8′), 120.3 (C6′), 120.2 (C10′, C14′), 108.8 (C4a), 107.3 (C19′, C23′), 106.2 (C8), 104.1 (C5), 70.5 (C6β, C7β), 69.0 (C6α), 68.8 (C7α), 60.6 (C21′O$\underline{C}H_3$), 58.9 (C6γ, C7γ), 56.7 (C20′O$\underline{C}H_3$, C22′O$\underline{C}H_3$); HRMS exact mass calcd. for $C_{40}H_{41}N_6O_8$ [M+H]$^+$, requires m/z: 733.2986, found m/z: 733.2969.

(E)-3-(4-(4-(3-((6,7-bis(2-methoxyethoxy)quinazolin-4-yl)amino)phenyl)-1H-1,2,3-triazol-1-yl)phenyl)-1-(ferrocenyl)prop-2-en-1-one (**5b**)

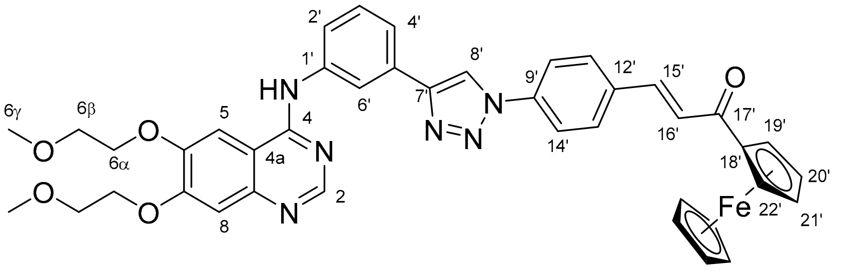

Scheme 10. Structure of **5b** with the numbering of atoms used for the assignment of NMR data.

Red solid; Yield: 0.47 g (63%); ^{1}H-NMR (DMSO-d_6): 9.61 (s, 1H, N<u>H</u>), 9.39 (s, 1H, H8′), 8.47 (s, br, 1H, H2), 8.34 (s, br, 1H, H6′), 8.11 (d, *J* = 8.5 Hz, 2H, H11′, H13′), 8.04 (d, *J* = 8.2 Hz, 2H, H10′, H14′), 7.97-7.80 (m, 2H, H5, H2′), 7.67 (d, *J* = 15.4 Hz, 1H, H17′), 7.64 (d, *J* = 8.3 Hz, 1H, H4′), 7.55-7.46 (m, 2H, H3′, H16′), 7.21 (s, 1H, H8), 5.06 (s, 2H, H19′, H22′), 4.66 (s, 2H, H20′, H21′), 4.31-4.22 (m, 4H, H6α, H7α), 4.21 (s, 5H, η^5-C_5<u>H_5</u>), 3.76 (s, 2H, H6β), 3.71 (s, 2H, H7β), 3.34 (s, 3H, H6γ), 3.30 (s, overlapped by HDO signal from solvent, 3H, H7γ); ^{13}C-NMR (DMSO-d_6): 192.4 (C17′), 156.8 (C4), 154.1 (C7), 153.4 (C2), 148.6 (C6), 147.9 (C7′), 147.6 (C1a), 140.6 (C1′), 138.8 (C15′), 137.8 (C9′), 135.7 (C12′), 130.9 (C5′), 130.6 (C11′, C13′), 129.6 (C3′), 125.2 (C16′), 122.8 (C2′), 121.1 (C4′), 120.6 (C10′, C14′), 120.1 (C8′), 119.5 (C6′), 108.7 (C8), 103.7 (C5), 81.1 (C18′), 73.4 (C20′, C21′), 70.6 (C6β), 70.5 (C7β), 70.3 (η^5-<u>C_5</u>H_5), 70.2 (C19′, C22′), 68.8 (C6α), 68.5 (C7α), 58.9 (C6γ), 58.8 (C7γ); HRMS exact mass calcd. for $C_{41}H_{39}N_6O_5Fe$ [M+H]$^+$, requires m/z: 751.2331, found m/z: 751.2318.

(E)-3-(4-(4-(3-((6,7-bis(2-methoxyethoxy)quinazolin-4-yl)amino)phenyl)-1H-1,2,3-triazol-1-yl)phenyl)-1-phenylprop-2-en-1-one (**5d**)

Following the general procedure **5c** was obtained, which Boc protecting group was cleaved by 2 M HCl to give free amine without purification of Boc protected derivative. To the crude product (**5c**) of previous reaction 2 M HCl (8.5 mL), and DMSO (2 mL) were added. The resulting mixture was stirred at room temperature for 12 h, then diluted with water (20 mL), carefully neutralized by the addition of solid $NaHCO_3$ in small portions and extracted with DCM (3 x 50 mL). The combined organic phase was washed with saturated NaCl solution, dried on Na_2SO_4, and evaporated to dryness. The residue was purified by flash column chromatography on silica, using gradient elution with DCM:MeOH 10:1 to 5:1. The analytical samples were crystallized by MeOH.

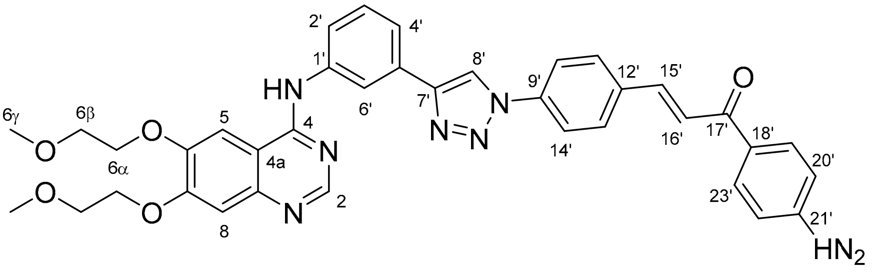

Scheme 11. Structure of **5d** with the numbering of atoms used for the assignment of NMR data.

Yellow solid; Yield: 0.15 g (22%): ^{1}H-NMR (DMSO-d_6): 9.59 (s, 1H, N<u>H</u>), 9.37 (s, 1H, H8′), 8.46 (s, 1H, H2), 8.34 (t, *J* = 1.9 Hz, 1H, H6′), 8.08 (d, *J* = 8.7 Hz, 2H, H11′, H13′), 8.02 (d, *J* = 8.7 Hz, 2H, H10′, H14′), 7.95 (d, *J* = 15.7 Hz, 1H, H16′), 7.92-7.88 (m, 4H, H5, H2′, H19′, H23′), 7.68-7.61 (m, 2H, H4′, H15′), 7.49 (t, *J* = 7.9 Hz, 1H, H3′), 7.20 (s, 1H, H8), 6.59 (d, *J* = 8.7 Hz, 2H, H20′, H22′), 6.15 (s, 1H, N<u>H_2</u>), 4.28 (dd, *J* = 5.9 Hz, 3.4 Hz, 2H, H6α), 4.25 (dd, *J* = 5.9 Hz, 3.5 Hz, 2H, H7α), 3.76 (dd, *J* = 5.9 Hz, 3.5 Hz, 2H, H6β), 3.71 (dd, *J* = 5.9 Hz, 3.5 Hz, 2H, H7β), 3.34 (s, 3H, H6γ), 3.32

(s, 3H, H7γ); ^{13}C-NMR (DMSO-d_6): 186.1 (C17′), 156.9 (C4), 154.5 (C21′), 154.1 (C7), 153.4 (C2), 148.6 (C6), 147.9 (C7′), 147.5 (C1a), 140.6 (C1′), 140.3 (C15′), 137.7 (C9′), 136.0 (C12′), 131.7 (C19′, C23′), 130.9 (C5′), 130.5 (C11′, C13′), 129.6 (C3′), 125.7 (C18′), 124.0 (C16′), 122.8 (C2′), 121.0 (C4′), 120.5 (C10′, C14′), 120.0 (C8′), 119.5 (C6′), 113.2 (C20′, C22′), 109.4 (C4a), 108.7 (C8), 103.7 (C5), 70.6 (C6β), 70.5 (C7β), 68.8 (C6α), 68.5 (C7α), 58.9 (C6γ), 58.8 (C7γ); HRMS exact mass calcd. for $C_{37}H_{36}N_7O_5$ [M+H]$^+$, requires m/z: 658.2778, found m/z: 658.2754.

(E)-3-(2-(4-(3-((6,7-bis(2-methoxyethoxy)quinazolin-4-yl)amino)phenyl)-1H-1,2,3-triazol-1-yl)phenyl)-1-(3,4,5-trimethoxyphenyl)prop-2-en-1-one (**6a**)

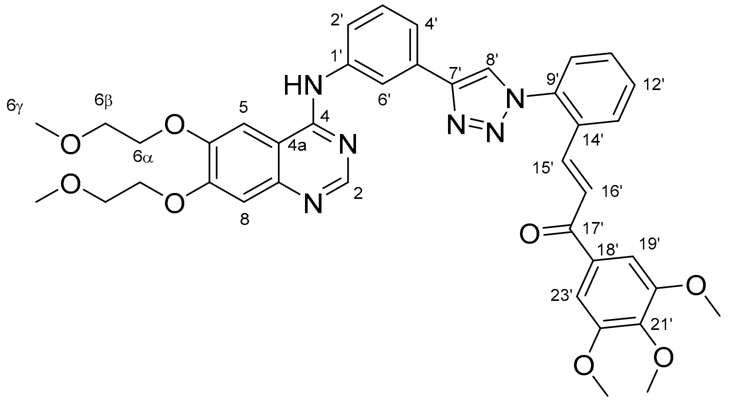

Scheme 12. Structure of **6a** with the numbering of atoms used for the assignment of NMR data.

Yellow solid; Yield: 0.24 g (33%); ^{1}H-NMR (DMSO-d_6): 9.57 (s, 1H, N<u>H</u>), 9.02 (s, 1H, H8′), 8.48 (s, br, 1H, H2), 8.38 (s, br, 1H, H6′), 8.35 (d, *J* = 6.7 Hz, 1H, C13′), 7.96-7.89 (m, 2H, H5, H16′), 7.88 (d, *J* = 8.4 Hz, 1H, H2′), 7.75-7.65 (m, 3H, H10′, H11′, H12′), 7.62 (d, *J* = 7.7 Hz, 1H, H4′), 7.48 (t, *J* = 7.9 Hz, 1H, H3′), 7.36 (d, *J* = 15.4 Hz, 1H, H15′), 7.33 (s, br, 2H, H19′, H23′), 7.29 (s, 1H, H8), 4.28 (t, *J* = 4.7 Hz, 2H, H6α), 4.25 (t, *J* = 4.6 Hz, 2H, H7α), 3.82 (s, 6H, C20′OC<u>H</u>$_3$, C22′OC<u>H</u>$_3$), 3.75 (t, *J* = 4.7 Hz, 2H, H6β), 3.73-3.68 (m, 5H, H7β overlapped by C21′OC<u>H</u>$_3$), 3.33 (s, 3H, H6γ), 3.31 (s, 3H, H7γ); ^{13}C-NMR (DMSO-d_6): 188.2 (C17′), 156.8 (C4), 154.0 (C7), 153.4 (C2, C20′, C22′), 148.6 (C6), 147.5 (C1a), 147.4 (C7′), 142.7 (C21′), 140.6 (C1′), 137.8 (C15′), 136.8 (C9′), 133.0 (C18′), 131.8 (C11′), 130.9 (C5′, C14′), 130.8 (C12′), 129.6 (C3′), 128.9 (C13′), 127.3 (C10′), 125.4 (C16′), 124.5 (C8′), 122.7 (C2′), 121.1 (C4′), 119.6 (C6′), 109.4 (C4a), 108.7 (C8), 106.8 (C19′, C23′), 103.9 (C5), 70.6 (C6β), 70.5 (C7β), 68.9 (C6α), 68.5 (C7α), 60.7 (C21′O<u>C</u>H$_3$) 58.9 (C6γ), 58.8 (C7γ), 56.7 (C20′O<u>C</u>H$_3$, C22′O<u>C</u>H$_3$); HRMS exact mass calcd. for $C_{40}H_{41}N_6O_8$ [M+H]$^+$, requires m/z: 733.2986, found m/z: 733.2958.

(E)-3-(2-(4-(3-((6,7-bis(2-methoxyethoxy)quinazolin-4-yl)amino)phenyl)-1H-1,2,3-triazol-1-yl)phenyl)-1-(ferrocenyl)prop-2-en-1-one (**6b**)

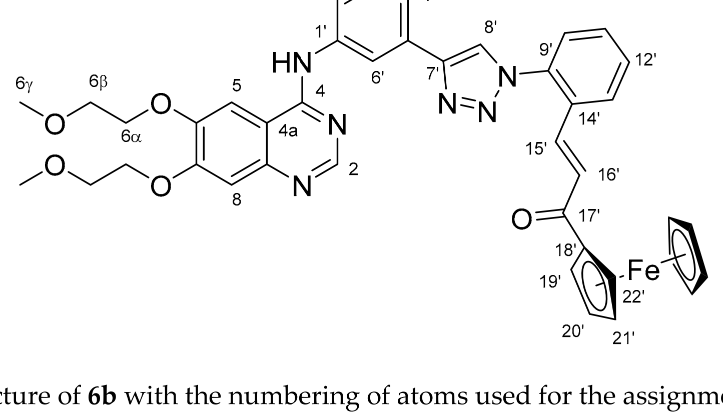

Scheme 13. Structure of **6b** with the numbering of atoms used for the assignment of NMR data.

Purple solid; Yield: 0.48 g (64%); ^{1}H-NMR (DMSO): 9.66 (br s, 1H, N<u>H</u>), 9.09 (s, 1H, H8′), 8.54 (br s, 1H, H2), 8.54 (br s, 1H, H2), 8.45 (s, 1H, H6′), 8.34 (d, *J* = 7.5 Hz, 1H, H13′), 7.93 (d, *J* = 8.4 Hz, 1H, H2′), 7.79-7.66 (m, 5H, H5, H4′, H10′, H11′, H12′), 7.53 (t, *J* = 7.9 Hz, 1H, H3′), 7.34-7.23 (m, 3H, H15′, H16′ overlapped by H8), 4.96 (t, *J* = 1.9 Hz, 2H, H19′, H22′), 4.66 (t, *J* = 1.9 Hz, 2H, H20′, H21′), 4.32 (t, *J* = 4.2 Hz, 2H, H6α), 4.29 (br s, 2H, H7α), 4.20 (s, 5H, η^5-$C_5\underline{H}_5$), 3.80 (t, *J* = 4.4 Hz, 2H, H6β), 3.75 (br s, 2H, H7β), 3.38 (s, 3H, H6γ), 3.36 (s, 3H, H7γ); ^{13}C-NMR (DMSO): 192.2 (C17′), 156.9 (C4), 154.1 (C7), 153.5 (C2), 148.9 (C6), 147.4 (C7′), 147.3 (C1a), 140.6 (C1′), 136.2 (C9′), 133.8 (C15′), 131.3 (C11′), 131.3 (C14′), 131.0 (C12′), 130.9 (C5′), 129.7 (C3′), 128.9 (C13′), 127.5 (C10′), 127.2 (C16′), 124.5 (C8′), 122.7 (C2′), 121.1 (C4′), 119.6 (C6′), 109.5 (C4a), 108.7 (C8), 103.7 (C5), 102.3, 80.7 (C18′), 73.5 (C20′, C21′), 70.6 (C6β), 70.5 (C7β), 70.4 (η^5-$\underline{C}_5H_5$), 70.2 (C19′, C22′), 68.9 (C6α), 68.5 (C7α), 58.9 (C6γ), 58.8 (C7γ); HRMS exact mass calcd. for $C_{41}H_{39}N_6O_5Fe$ $[M+H]^+$, requires m/z: 751.2331, found m/z: 751.2308.

(E)-1-(4-aminophenyl)-3-(2-(4-(3-((6,7-bis(2-methoxyethoxy)quinazolin-4-yl)amino)phenyl)-1H-1,2,3-triazol-1-yl)phenyl)prop-2-en-1-one (**6d**)

Following the general procedure **6c** was obtained, which was underwent immediately an acidic hydrolysis with HCl to give free amine without purification of boc protected derivative. To the crude product (**6c**) of previous reaction concentrated HCl (5 mL), and methanol (15 mL) were added. The resulting mixture was stirred at room temperature for 12 h, then diluted with water (30 mL), carefully neutralized by the addition of solid $NaHCO_3$ in small portions and extracted with DCM (3 x 50 mL). The combined organic phase was washed with saturated NaCl solution, dried on Na_2SO_4, and evaporated to dryness. The residue was purified by flash column chromatography on silica, using gradient elution with DCM:MeOH 15:1 to 10:1. The analytical samples were crystallized by Et_2O.

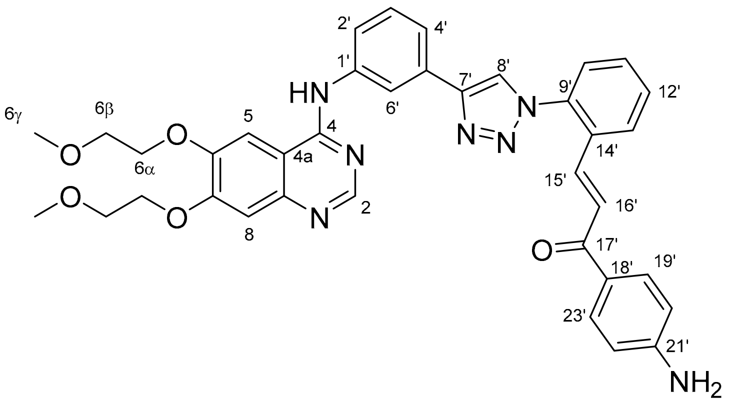

Scheme 14. Structure of **6d** with the numbering of atoms used for the assignment of NMR data.

Yellow solid; Yield: 97.3 mg (23%); ^{1}H-NMR (DMSO-d_6): 9.57 (s, 1H, N<u>H</u>), 9.00 (s, 1H, H8′), 8.47 (s, br, 1H, H2), 8.37 (t, *J* = 2.0 Hz, 1H, H6′), 8.30 (d, *J* = 7.6 Hz, 1H, H13′), 7.94-7.88 (m, 2H, H5, H2′), 7.87-7.80 (m, 3H, H16′, H19′, H23′), 7.71-7.60 (m, 4H, H4′, H10′, H11′, H12′), 7.48 (t, *J* = 7.9 Hz, 1H, H3′), 7.23 (d, *J* = 15.4 Hz, 1H, H15′), 7.20 (s, 1H, H8), 6.55 (d, *J* = 8.5 Hz, 2H, H20′, H22′), 6.15 (s, 1H, $N\underline{H}_2$), 4.28 (t, *J* = 4.7 Hz, 2H, H6α), 4.25 (t, *J* = 4.5 Hz, 2H, H7α), 3.75 (t, *J* = 4.8 Hz, 2H, H6β), 3.71 (t, *J* = 4.8 Hz, 2H, H7β), 3.33 (s, 3H, H6γ), 3.31 (s, 3H, H7γ); ^{13}C-NMR (DMSO-d_6): 185.7 (C17′), 156.8 (C4), 154.6 (C7), 154.1 (C21′), 153.4 (C2), 148.6 (C6), 147.3 (C1a, C7′), 140.7 (C1′), 136.6 (C9′), 135.2 (C15′), 131.7 (C19′, C23′), 131.5 (C14′), 131.2 (C11′), 130.9 (C12′), 130.8 (C5′), 129.6 (C3′), 128.5 (C13′), 127.3 (C10′), 126.0 (C16′), 125.4 (C18′), 124.5 (C8′), 122.7 (C2′), 121.1 (C4′), 119.5 (C6′), 113.2 (C20′, C22′), 109.5 (C4a), 108.6 (C8), 103.8 (C5), 70.6 (C6β), 70.5 (C7β), 68.9 (C6α), 68.5 (C7α), 58.9 (C6γ), 58.8 (C7γ); HRMS exact mass calcd. for $C_{37}H_{36}N_7O_5$ $[M+H]^+$, requires m/z: 658.2778, found m/z: 658.2750.

(E)-3-(4-(4-(3-((6,7-bis(2-methoxyethoxy)quinazolin-4-yl)amino)phenyl)-1H-1,2,3-triazol-1-yl)phenyl)-1-(3,4,5-trimethoxyphenyl)prop-2-en-1-one (**7a**)

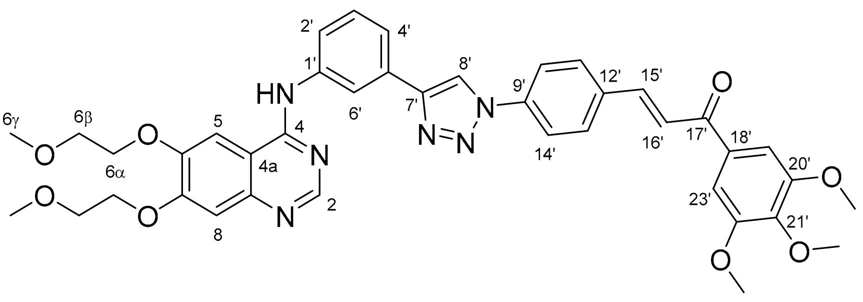

Scheme 15. Structure of **7a** with the numbering of atoms used for the assignment of NMR data.

Orange solid; Yield: 0.55 g (75%); ^{1}H-NMR (DMSO-*d*$_6$): 9.58 (s, 1H, NH), 9.37 (s, 1H, H8′), 8.46 (s, 1H, H2), 8.34 (t, *J* = 1.9 Hz, 1H, H6′), 8.14 (d, *J* = 8.4 Hz, 2H, H11′, H13′), 8.05 (d, *J* = 8.4 Hz, 2H, H10′, H14′), 8.01 (d, *J* = 15.5 Hz, 1H, H16′), 7.92-7.88 (m, 2H, H5, H2′), 7.78 (d, *J* = 15.5 Hz, 1H, H15′), 7.63 (dt, *J* = 7.8 Hz, 1.4 Hz, 1H, H4′), 7.48 (t, *J* = 7.9 Hz, 1H, H3′), 7.42 (s, 2H, H19′, H23′), 7.19 (s, 1H, H8), 4.28 (t, *J* = 4.8 Hz, 2H, H6α), 4.25 (t, *J* = 4.5 Hz, 2H, H7α), 3.88 (s, 6H, C20′OCH$_3$, C23′OCH$_3$), 3.76 (dd, *J* = 5.9 Hz, 3.3 Hz, 2H, H6β), 3.74 (s, 3H, C21′OCH$_3$), 3.71 (dd, *J* = 6.0 Hz, 2.7 Hz, 2H, H7β), 3.34 (s, 3H, H6γ), 3.31 (s, overlapped by HDO signal from solvent, 3H, H7γ); ^{13}C-NMR (DMSO-*d*$_6$): 188.3 (C17′), 156.9 (C4), 154.1 (C7), 153.4 (C2, C20′, C22′), 148.6 (C6), 148.0 (C1a, C7′), 142.8 (C21′), 142.6 (C15′), 140.6 (C1′), 138.1 (C9′), 135.5 (C12′), 133.3 (C18′), 131.0 (C11′, C13′), 130.8 (C5′), 129.6 (C3′), 123.4 (C16′), 122.8 (C2′), 121.1 (C4′), 120.5 (C10′, C14′), 120.1 (C8′), 119.5 (C6′), 109.5 (C4a), 108.7 (C8), 106.8 (C19′, C23′), 103.7 (C5), 70.6 (C6β), 70.5 (C7β), 68.9 (C6α), 68.5 (C7α), 60.7 (C21′OCH$_3$), 58.9 (C6γ), 58.8 (C7γ), 56.8 (C20′OCH$_3$, C22′OCH$_3$); HRMS exact mass calcd. for $C_{40}H_{41}N_6O_8$ [M+H]$^+$, requires m/z: 733.2986, found m/z: 733.2953.

(E)-1-(4-(4-(3-((6,7-bis(2-methoxyethoxy)quinazolin-4-yl)amino)phenyl)-1H-1,2,3-triazol-1-yl)phenyl)-3-(ferrocenyl)prop-2-en-1-one (**7b**)

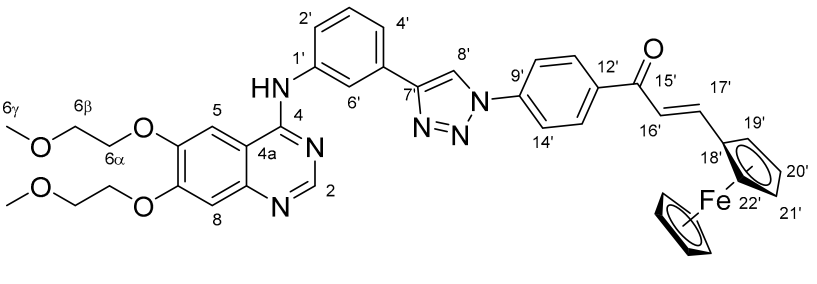

Scheme 16. Structure of **7b** with the numbering of atoms used for the assignment of NMR data.

Purple solid; Yield: 0.64 g (85%); ^{1}H-NMR (DMSO-*d*$_6$): 9.61 (s, 1H, NH), 9.45 (s, 1H, H8′), 8.48 (s, br, 1H, H2), 8.35 (t, *J* = 1.9 Hz, 1H, H6′), 8.29 (d, *J* = 8.5 Hz, 2H, H11′, H13′), 8.14 (d, *J* = 8.5 Hz, 2H, H10′, H14′), 7.94-7.88 (m, 2H, H5, H2′), 7.70 (d, *J* = 15.2 Hz, 1H, H17′), 7.65 (d, *J* = 7.7 Hz, 1H, H4′), 7.53-7.45 (m, 2H, H3′, H16′), 7.22 (s, br, 1H, H8), 4.86 (t, *J* = 1.9 Hz, 2H, H19′, H22′), 4.54 (t, *J* = 1.8 Hz, 2H, H20′, H21′), 4.28 (t, *J* = 4.7, 2H, H6α), 4.25 (t, *J* = 4.5 Hz, 2H, H7α), 4.17 (s, 5H, η^5-C$_5$H$_5$), 3.76 (dd, *J* = 5.7 Hz, 6.8 Hz, 2H, H6β), 3.71 (dd, *J* = 5.9 Hz, 3.4 Hz, 2H, H7β), 3.34 (s, 3H, H6γ), 3.31 (s, overlapped by HDO signal from solvent, 3H, H7γ); ^{13}C-NMR (DMSO-*d*$_6$): 187.4 (C15′), 156.9 (C4), 154.0 (C2, C7), 148.6 (C6, C1a), 148.1 (C7′), 147.7 (C17′), 140.6 (C1′), 139.7 (C9′), 138.1 (C12′), 130.7 (C5′, C11′, C13′), 129.7 (C3′), 122.9 (C2′), 121.1 (C4′), 120.2 (C8′, C10′, C14′), 119.5 (C6′), 119.0 (C16′), 109.1 (C8), 103.7 (C5), 79.5 (C18′), 72.0 (C20′, C21′), 70.6 (C6β), 70.5 (C7β), 70.1 (η^5-C$_5$H$_5$), 69.9 (C19′, C22′), 68.8 (C6α), 68.5 (C7α), 58.9 (C6γ), 58.8 (C7γ); HRMS exact mass calcd. for $C_{41}H_{39}N_6O_5Fe$ [M+H]$^+$, requires m/z: 751.2331, found m/z: 751.2303.

1.2.2.2 Synthesis of novel Erlotinib hybrids with alkyne linker

Erlotinib (**1**) (0.39 g, 1.0 mmol, 1.0 eq.), 2-iodobenzaldehyde (**9**), 4-bromobenzaldehyde (**8**) (1.0 mmol, 1.0 eq.), CuI (40.0 mg, 0.2 mmol, 0.2 eq.) and $Pd(PPh_3)_2Cl_2$ (70.0 mg, 0.1 mmol, 0.1 eq.) were suspended in a mixture of DIPEA (10 mL) and DMF (3 mL) The resulting clean solution was stirred at room temperature for 12 h, diluted with water (25 mL) and extracted with DCM (3 x 50 mL). The combined organic phase was washed with saturated NaCl solution, dried on Na_2SO_4, and evaporated to dryness. The residue was purified by flash chromatography on silica gel, using DCM:MeOH mixtures (the ratio of DCM and MeOH was varied from 20:1 to 10:1). The analytical samples were crystallized by MeOH-water or Et_2O.

4-((3-((6,7-bis(2-methoxyethoxy)quinazolin-4-yl)amino)phenyl)ethynyl)benzaldehyde (**10**)

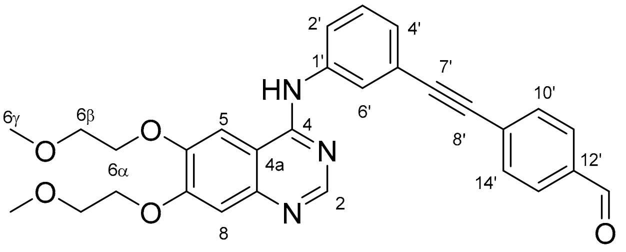

Scheme 17. Structure of **10** with the numbering of atoms used for the assignment of NMR data.

Yellow solid; Yield: 0.22 g (44%); ^{1}H-NMR (DMSO-d_6): 10.00 (s, 1H, C<u>H</u>O), 9.47 (s, 1H, N<u>H</u>), 8.49 (s, 1H, H2), 8.06 (t, J = 1.9 Hz, 1H, H6′), 7.96-7.89 (m, 3H, H2′, H11′, H13′), 7.84 (s, br, 1H, H5), 7.75 (d, J = 8.2 Hz, 2H, H10′, H14′), 7.43 (t, J = 7.9 Hz, 1H, H3′), 7.29 (dt, J = 7.6 Hz, 1.2 Hz, 1H, H4′), 7.20 (s, br, 1H, H8), 4.29-4.22 (m, 4H, H6α, H7α), 3.75 (t, J = 4.7 Hz, 2H, H6β), 3.71 (t, J = 4.4 Hz, 2H, H7β), 3.33 (s, 3H, H6γ), 3.31 (s, 3H, H7γ); ^{13}C-NMR (DMSO-d_6): 192.9 (<u>C</u>HO), 156.5 (C4), 154.2 (C7), 153.2 (C2), 148.7 (C6), 147.7 (C1a), 140.4 (C1′), 136.0 (C12′), 132.5 (C10′, C14′), 130.2 (C11′, C13′), 129.6 (C3′), 128.6 (C9′), 126.7 (C4′), 125.0 (C6′), 123.4 (C2′), 122.1 (C5′), 108.9 (C8), 103.7 (C5), 93.4 (C7′), 88.9 (C8′), 70.6 (C6β), 70.5 (C7β), 68.9 (C6α), 68.5 (C7α), 58.9 (C6γ), 58.8 (C7γ); HRMS exact mass calcd. for $C_{29}H_{28}N_3O_5$ $[M+H]^+$, requires m/z: 498.2029, found m/z: 498.2014.

2-((3-((6,7-bis(2-methoxyethoxy)quinazolin-4-yl)amino)phenyl)ethynyl)benzaldehyde (**11**)

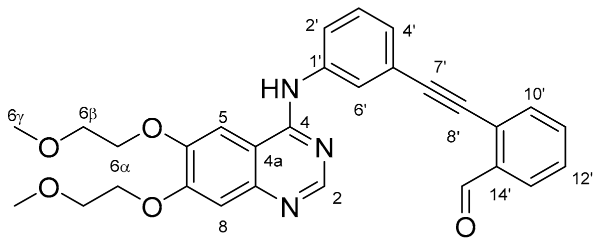

Scheme 18. Structure of **11** with the numbering of atoms used for the assignment of NMR data.

Yellow solid; Yield: 0.24 g (48%); ^{1}H-NMR (DMSO-d_6): 10.48 (s, 1H, C<u>H</u>O), 9.50 (s, 1H, N<u>H</u>), 8.48 (s, br, 1H, H2), 8.04 (s, br, 1H, H6′), 7.95 (d, J = 8.2 Hz, 1H, H2′), 7.91-7.83 (m, 2H, H5, H13′), 7.75-7.67 (m, 2H, H10′, H11′), 7.57 (t, J = 6.4 Hz, 1H, H12′), 7.44 (t, J = 7.9 Hz, 1H, H3′), 7.35 (d, J = 7.7 Hz, 1H, H4′), 7.20 (s, br, 1H, H8), 4.29-4.22 (m, 4H, H6α, H7α), 3.75 (t, J = 4.8 Hz, 2H, H6β), 3.71 (t, J = 4.5 Hz, 2H, H7β), 3.33 (s, 3H, H6γ), 3.31 (s, overlapped by HDO signal from solvent, 3H, H7γ); ^{13}C-NMR (DMSO-d_6): 191.8 (<u>C</u>HO), 156.6 (C4), 154.2 (C7), 153.2 (C2), 148.7 (C6), 147.8 (C1a), 140.4 (C1′), 136.0 (C14′), 134.8 (C11′), 133.9 (C10′), 129.8 (C12′), 129.5 (C3′), 128.4 (C13′), 126.9 (C4′), 125.5 (C9′), 125.1 (C6′), 123.6 (C2′), 122.2 (C5′), 109.5 (C4a), 108.8 (C8), 103.7 (C5), 96.2 (C7′), 85.6 (C8′), 70.6 (C6β), 70.5 (C7β), 68.9 (C6α), 68.6 (C7α), 58.9 (C6γ), 58.8 (C7γ); HRMS exact mass calcd. for $C_{29}H_{28}N_3O_5$ $[M+H]^+$, requires m/z: 498.2029, found m/z: 498.2008.

(E)-3-(4-((3-((6,7-bis(2-methoxyethoxy)quinazolin-4-yl)amino)phenyl)ethynyl)phenyl)-1-(3,4,5-trimethoxyphenyl)prop-2-en-1-one (**13**)

4-((3-((6,7-Bis(2-methoxyethoxy)quinazolin-4-yl)amino)phenyl)ethynyl)benzaldehyde (**10**) (0.22 g, 0.4 mmol, 1.0 eq.) and 3,4,5-trimethoxyacetophenone (**12a**) (90.0 mg, 0.4 mmol, 1.0 eq.) were dissolved in EtOH (2 mL), after about 5 minutes of stirring at room temperature 10% solution of NaOH (2 mL) was added. The resulting reaction mixture was stirred for 12 h at room temperature, then poured on water (25 mL). The obtained precipitated solid was separated by filtration, washed with water and recrystallized from MeOH.

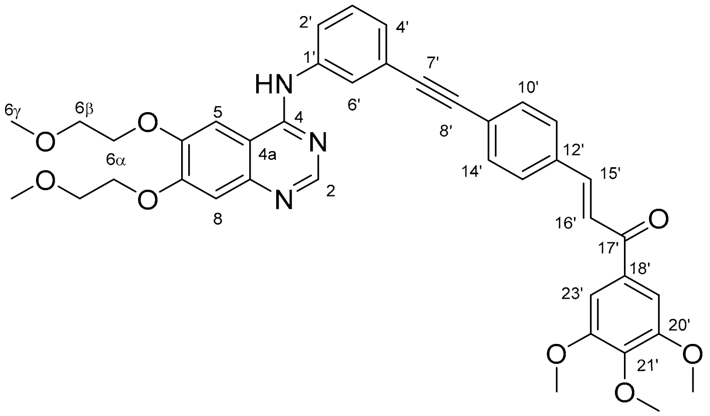

Scheme 19. Structure of **13** with the numbering of atoms used for the assignment of NMR data.

Yellow solid; Yield: 84 mg (30%); ^{1}H-NMR (DMSO-*d*$_6$): 9.47 (s, 1H, NH), 8.48 (s, 1H, H2), 8.05 (s, br, 1H, H6′), 8.00-7.88 (m, 4H, H2′, H11′, H13′, H16′), 7.85 (s, 2H, H5), 7.73 (d, *J* = 15.5 Hz, 1H, H15′), 7.62 (d, *J* = 7.9 Hz, 2H, H10′, H14′), 7.42 (s, br, 1H, H3′), 7.41 (s, 2H, H19′, H23′), 7.28 (d, *J* = 7.7 Hz, 1H, H4′), 7.20 (s, br, 1H, H8), 4.30-4.22 (m, 4H, H6α, H7α), 3.87 (s, 6H, C20′OCH_3, C22′OCH_3), 3.78-3.63 (m, 7H, H6β, H7β, C21′OCH_3), 3.34 (s, 3H, H6γ), 3.31 (s, 3H, H7γ); ^{13}C-NMR (DMSO-*d*$_6$): 188.2 (C17′), 156.6 (C4), 154.2 (C7), 153.4 (C20′, C22′), 153.3 (C2), 148.7 (C6), 147.5 (C1a), 143.2 (C15′), 142.6 (C21′), 140.4 (C1′), 135.5 (C12′), 133.4 (C18′), 132.3 (C10′, C14′), 129.8 (C11′, C13′), 129.5 (C3′), 126.6 (C4′), 124.9 (C6′), 124.6 (C9′), 123.3 (C16′), 123.1 (C2′), 122.5 (C5′), 109.4 (C4a), 108.7 (C8), 106.8 (C19′, C23′), 103.7 (C5), 92.0 (C7′), 89.6 (C8′), 70.6 (C6β), 70.5 (C7β), 68.9 (C6α), 68.5 (C7α), 60.7 (C21′OCH$_3$), 58.9 (C6γ), 58.8 (C7γ), 56.7 (C20′OCH$_3$, C22′OCH$_3$); HRMS exact mass calcd. for $C_{40}H_{40}N_3O_8$ [M+H]$^+$, requires m/z: 690.2815, found m/z: 690.2797.

(E)-3-(2-((3-((6,7-bis(2-methoxyethoxy)quinazolin-4-yl)amino)phenyl)ethynyl)phenyl)-1-(3,4,5-trimethoxyphenyl)prop-2-en-1-one (**14**)

2-((3-((6,7-Bis(2-methoxyethoxy)quinazolin-4-yl)amino)phenyl)ethynyl)benzaldehyde (**11**) (0.22 g, 0.4 mmol, 1.0 eq.) and 3,4,5-trimethoxyacetophenone (**12a**) (90.0 mg, 0.4 mmol, 1.0 eq.) were dissolved in EtOH (2 mL), after about 5 minutes of stirring at room temperature powdered NaOH (15.0 mg, 0.4 mmol, 1.0 eq.) was added. The resulting mixture was stirred for 12 h at room temperature, then diluted with water (25 mL) and extracted with DCM (3 x 25 mL). The combined organic phase was washed with saturated NaCl solution, dried on Na_2SO_4, and evaporated to dryness. The residue was purified by flash chromatography on silica gel, using DCM:MeOH=20:1. After all, the analytical sample was crystallized by Et_2O.

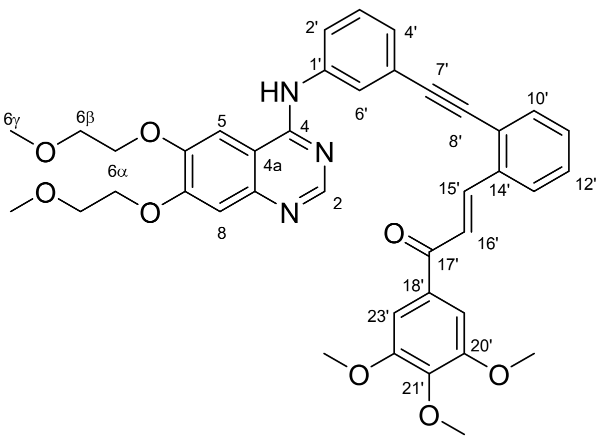

Scheme 20. Structure of **14** with the numbering of atoms used for the assignment of NMR data.

Yellow solid; Yield: 57.3 mg (20%); ^{1}H-NMR (DMSO-*d*$_6$): 9.50 (s, 1H, N<u>H</u>), 8.49 (s, 1H, H2), 8.28 (d, *J* = 15.6 Hz, 1H, H15′), 8.22 (dd, *J* = 6.9 Hz, 2.1 Hz, 1H, H13′), 8.10 (t, *J* = 1.9 Hz, 1H, H6′), 8.04 (d, *J* = 15.6 Hz, 1H, H16′), 7.91 (dd, *J* = 8.2 Hz, 1.9 Hz, 1H, H2′), 7.85 (s, br, 1H, H5), 7.65 (dd, *J* = 6.4 Hz, 2.7 Hz, 1H, H10′), 7.51-7.47 (m, 2H, H11′, H12′), 7.45 (t, *J* = 7.9 Hz, 1H, H3′), 7.40 (s, 2H, H19′, H23′), 7.28 (d, *J* = 7.6 Hz, 1H, H4′), 7.19 (s, br, 1H, H8), 4.29-4.22 (m, 4H, H6*α*, H7*α*), 3.83 (s, 6H, C20′OC<u>H</u>$_3$, C22′OC<u>H</u>$_3$), 3.77-3.61 (m, 7H, H6β, H7β overlapped by C21′OC<u>H</u>$_3$), 3.33 (s, 3H, H6γ), 3.31 (s, 3H, H7γ); ^{13}C-NMR (DMSO-*d*$_6$): 188.4 (C17′), 156.6 (C4), 154.2 (C7), 153.4 (C2), 153.3 (C20′, C22′), 148.6 (C6), 147.5 (C1a), 142.6 (C21′), 141.3 (C15′), 140.5 (C1′), 136.1 (C14′), 133.2 (C18′), 133.1 (C10′), 131.0 (C11′), 129.7 (C12′), 129.6 (C3′), 127.7 (C13′), 126.5 (C4′), 124.7 (C6′), 124.1 (C16′), 124.0 (C9′), 123.3 (C2′), 122.4 (C5′), 109.4 (C4a), 108.7 (C8), 106.8 (C19′, C23′), 103.7 (C5), 95.8 (C7′), 87.3 (C8′), 70.6 (C6β), 70.5 (C7β), 68.9 (C6*α*), 68.5 (C7*α*), 60.7 (C21′O<u>C</u>H$_3$), 58.9 (C6γ), 58.8 (C7γ), 56.6 (C20′O<u>C</u>H$_3$, C22′O<u>C</u>H$_3$); HRMS exact mass calcd. for $C_{40}H_{40}N_3O_8$ [M+H]$^+$, requires m/z: 690.2815, found m/z: 690.2798.

2 Copies of NMR spectra

2.1 NMR spectra of novel chalcone analogues

Tert-butyl (E)-(4-(3-(4-azidophenyl)acryloyl)phenyl)carbamate (**2c**)

^{1}H-NMR (500 MHz, $CDCl_3$)

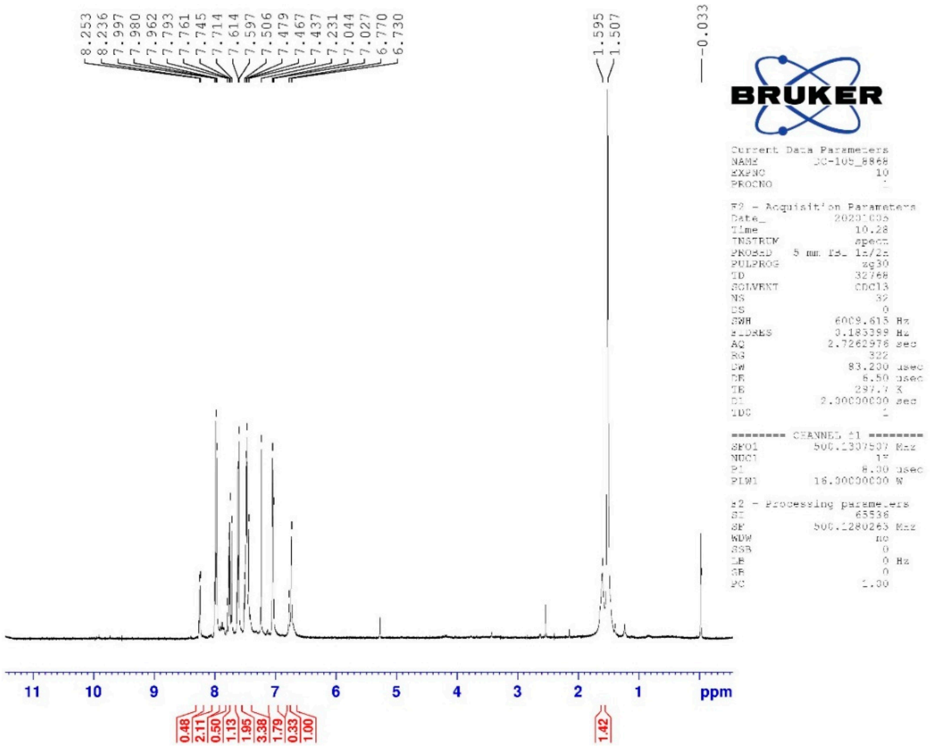

^{13}C-NMR (125 MHz, $CDCl_3$)

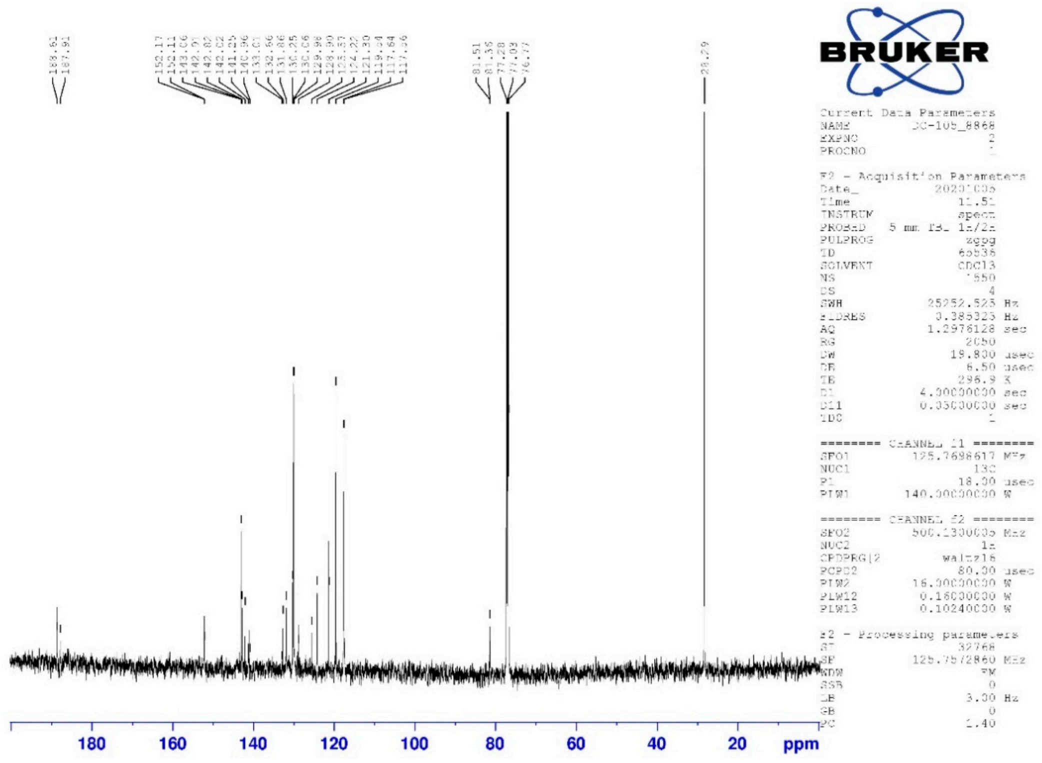

(E)-1-(4-aminophenyl)-3-(4-azidophenyl)prop-2-en-1-one (**2d**)

^{1}H-NMR (500 MHz, DMSO-d_6)

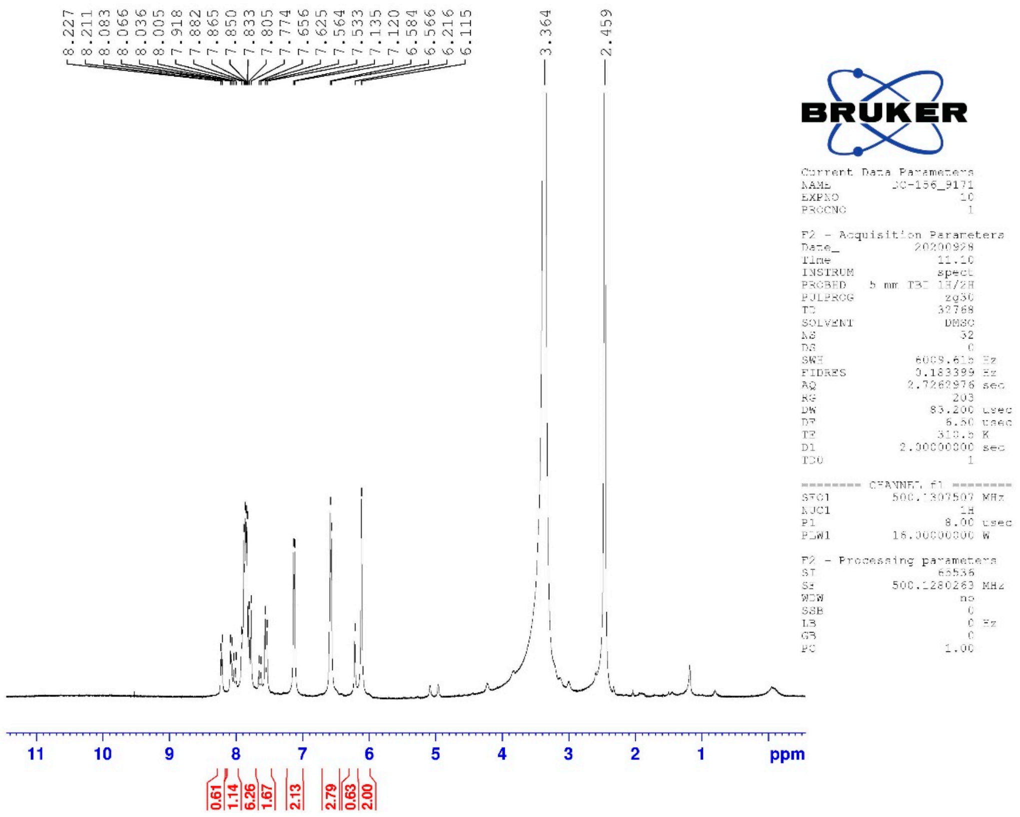

^{13}C-NMR (125 MHz, DMSO-d_6)

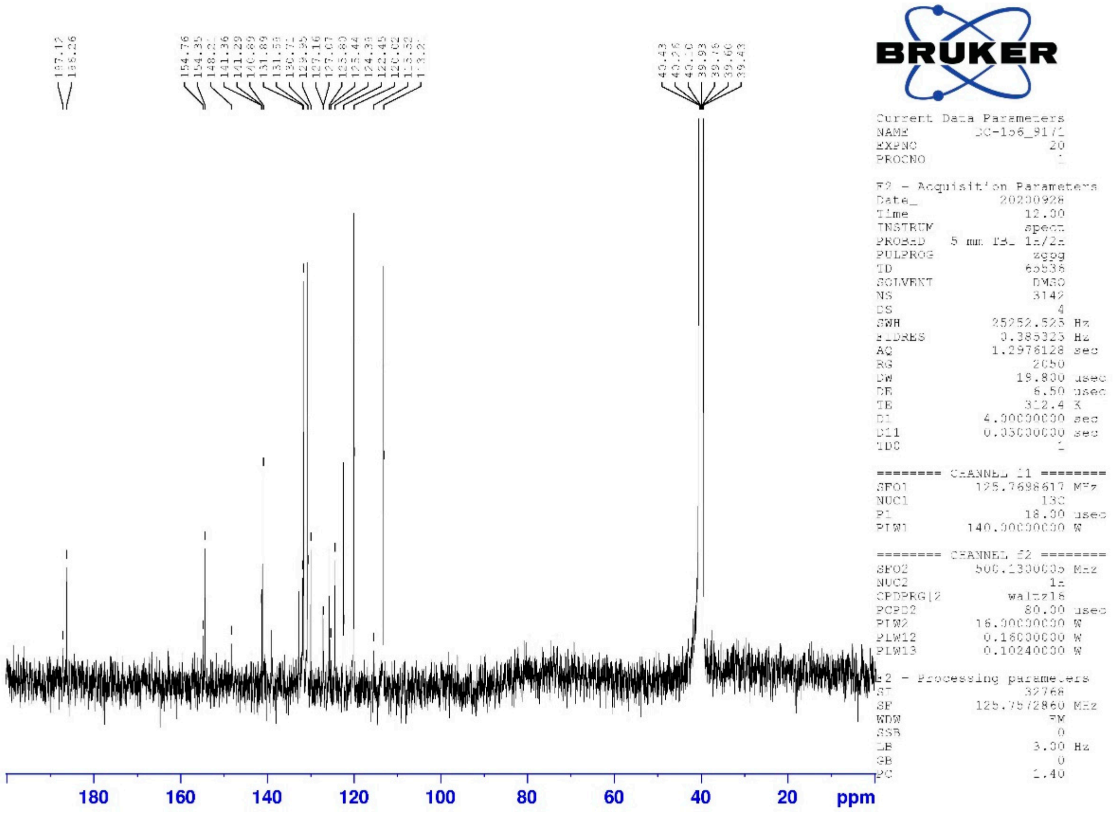

Tert-butyl (E)-(4-(3-(2-azidophenyl)acryloyl)phenyl)carbamate (**3c**)

^{1}H-NMR (500 MHz, $CDCl_3$)

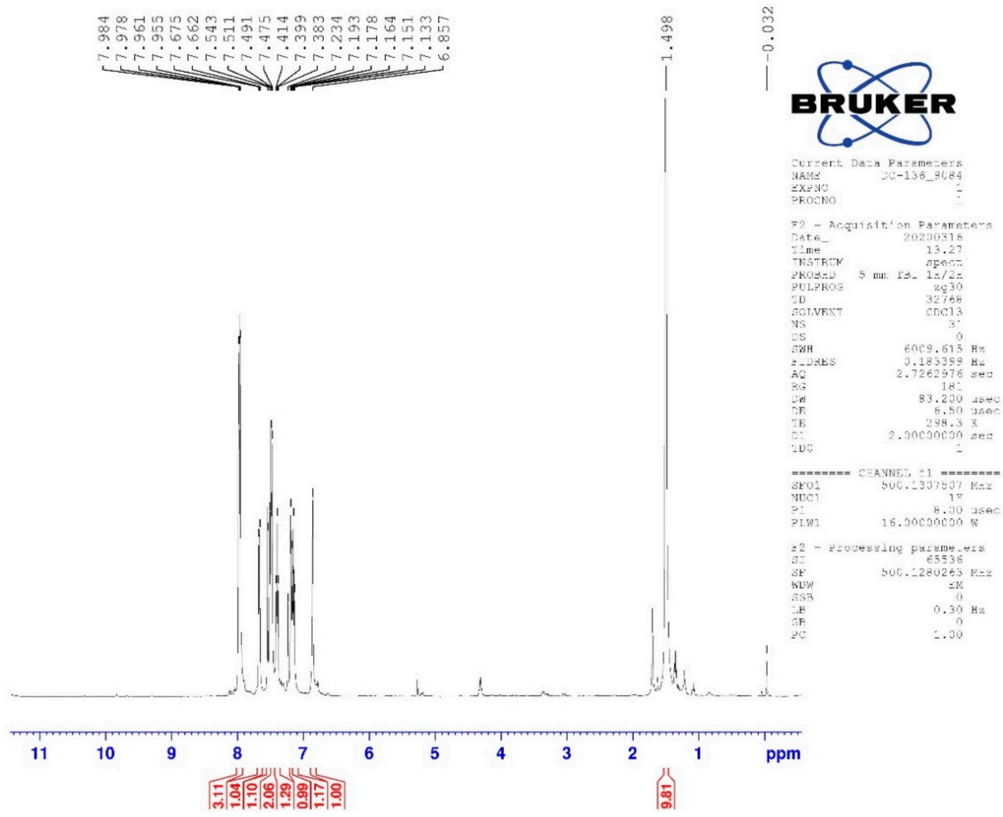

^{13}C-NMR (125 MHz, $CDCl_3$)

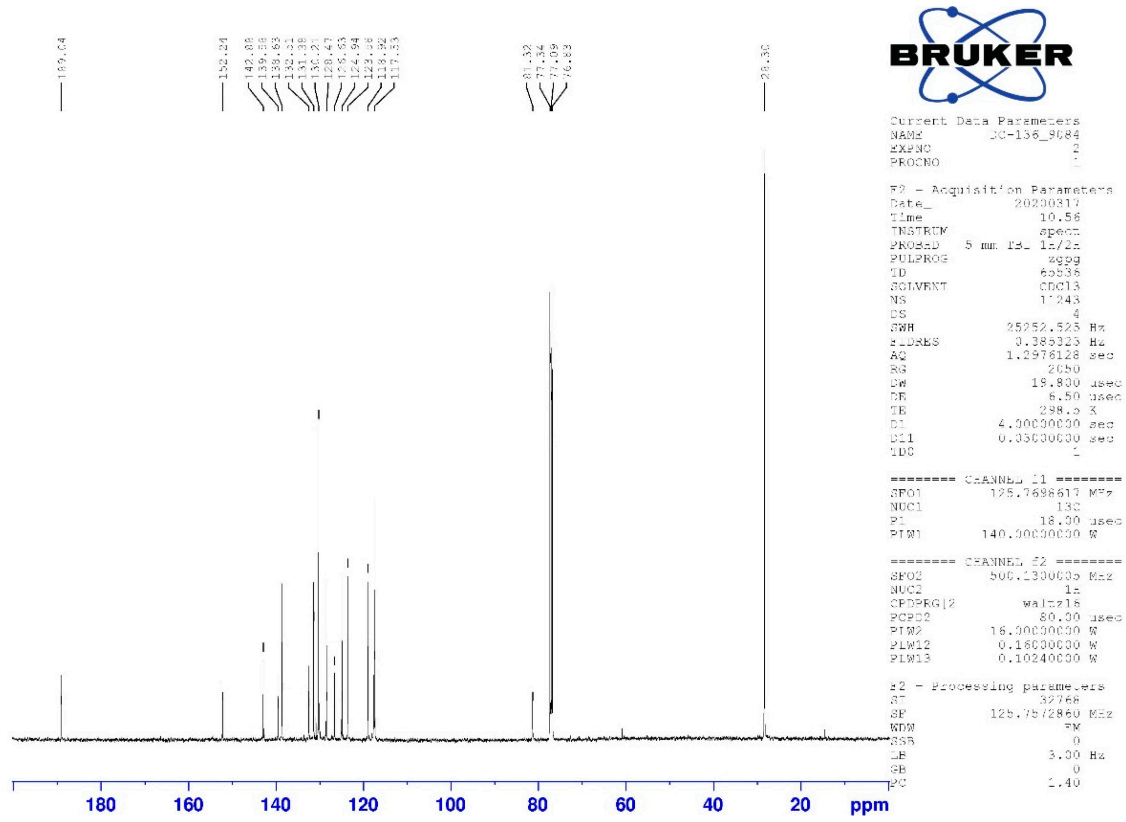

(E)-1-(4-aminophenyl)-3-(2-azidophenyl)prop-2-en-1-one (**3d**)

^{1}H-NMR (500 MHz, DMSO-d_6)

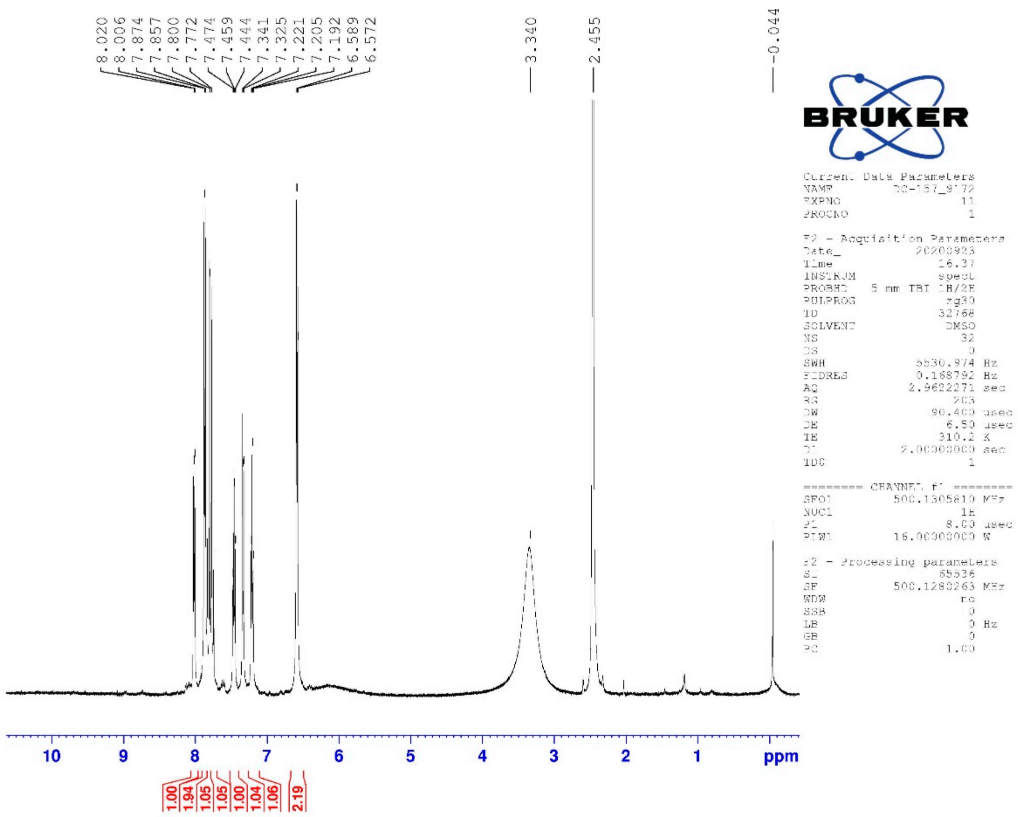

^{13}C-NMR (125 MHz, DMSO-d_6)

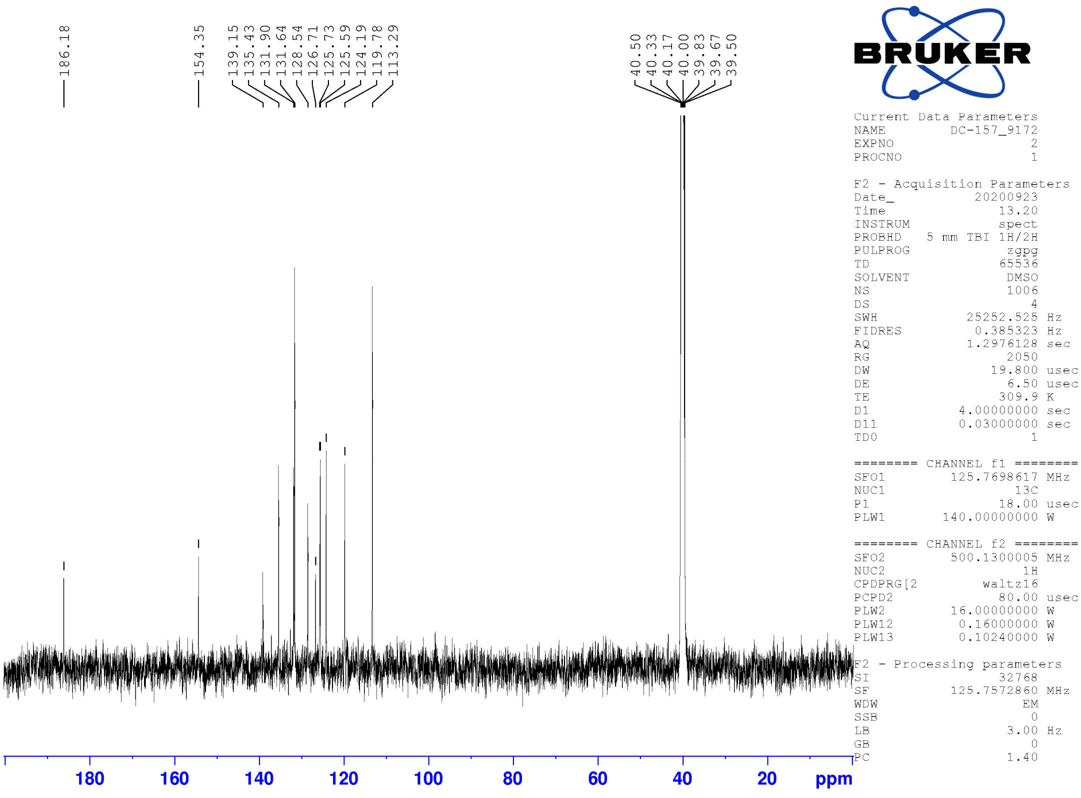

(E)-1-(4-azidophenyl)-3-(ferrocenyl)prop-2-en-1-one (**4b**)

^{1}H-NMR (500 MHz, $CDCl_3$)

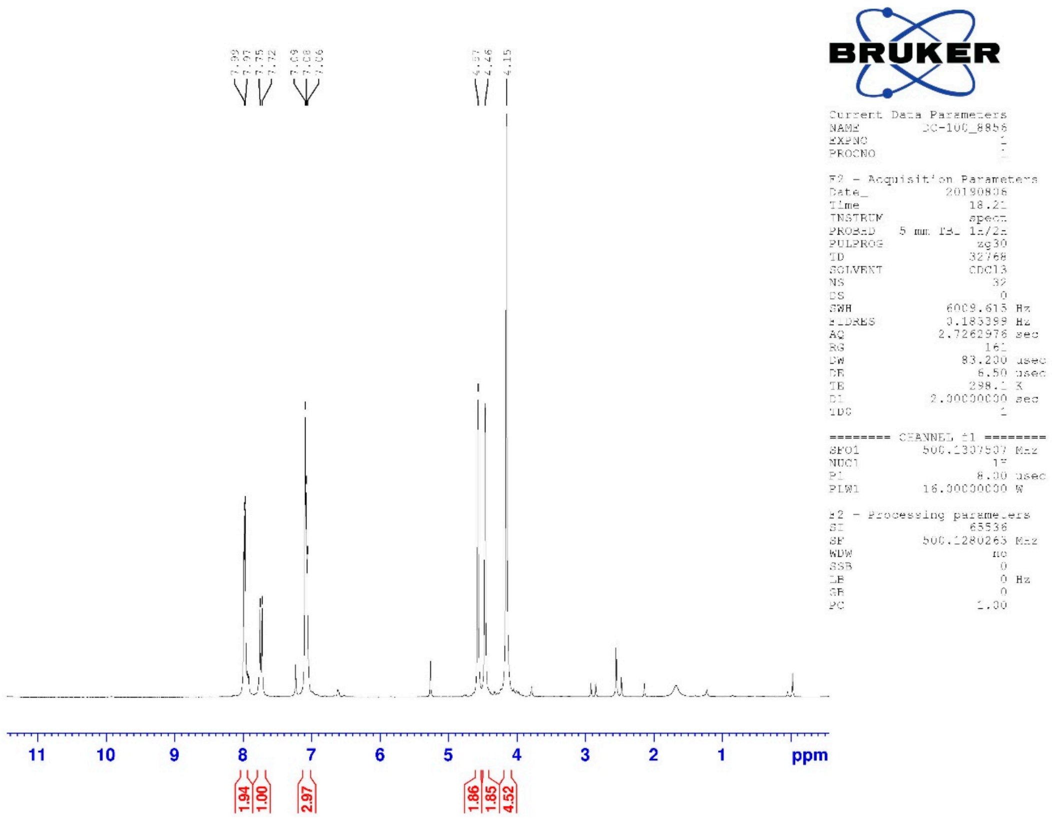

^{13}C-NMR (125 MHz, $CDCl_3$)

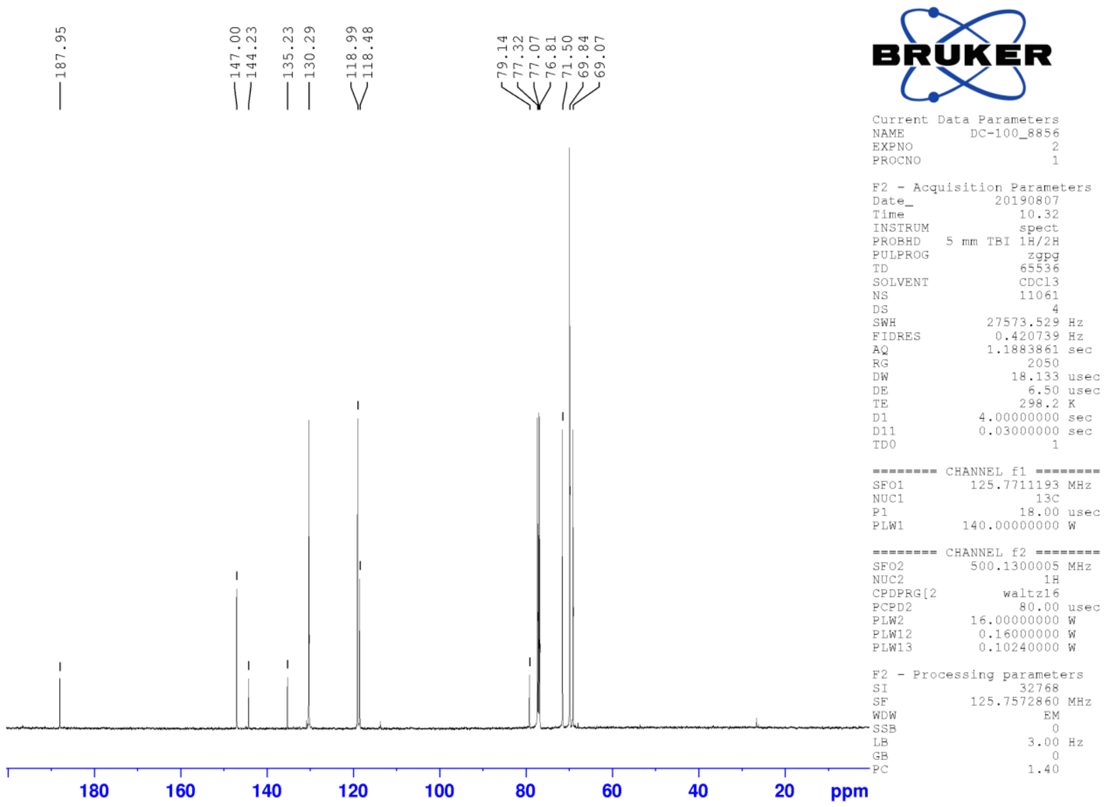

(E)-3-phenyl-1-(3,4,5-trimethoxyphenyl)prop-2-en-1-one (**15**)

^{1}H-NMR (400 MHz, DMSO-d_6)

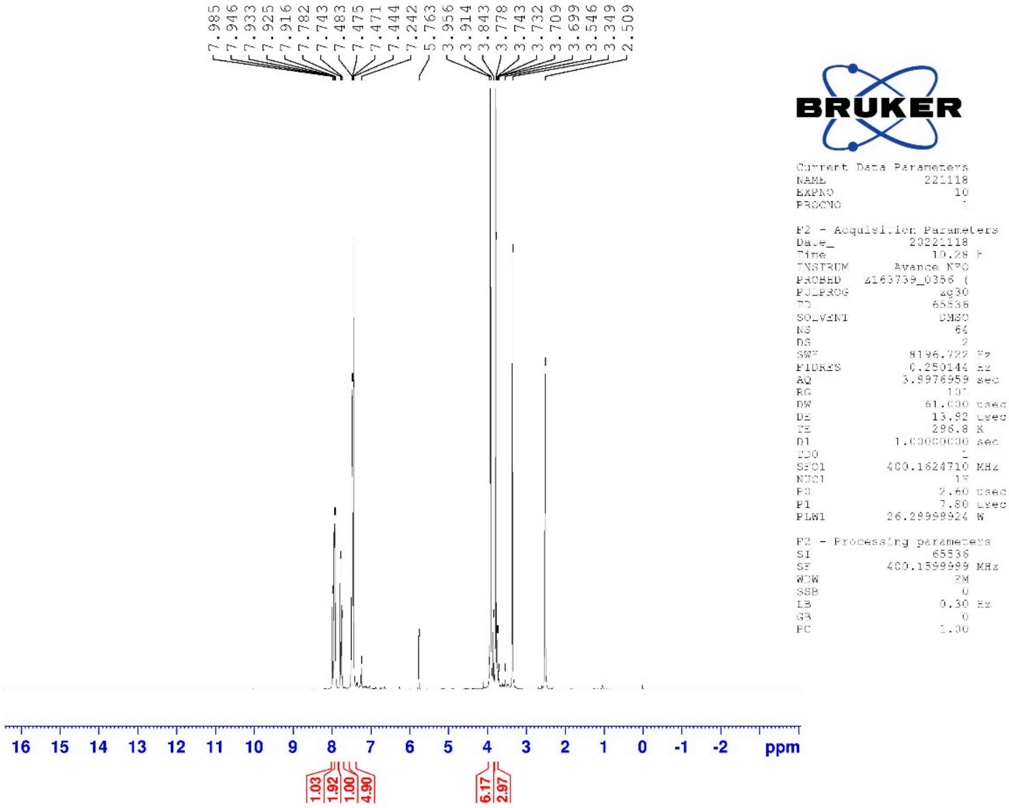

^{13}C-NMR (100 MHz, DMSO-d_6)

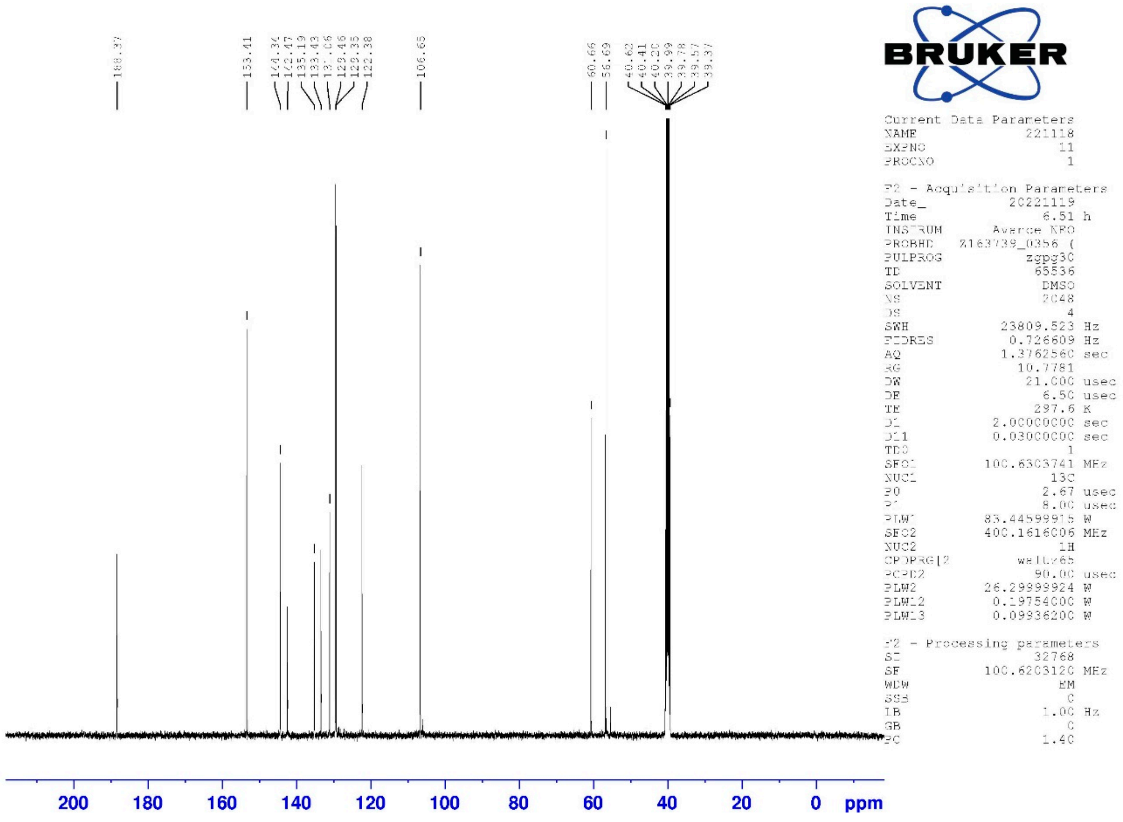

2.2 *NMR spectra of novel Erlotinib hybrids with 1,4-disubstituted triazole linkers*

(E)-1-(4-(4-(3-((6,7-bis(2-methoxyethoxy)quinazolin-4-yl)amino)phenyl)-1H-1,2,3-triazol-1-yl)phenyl)-3-(3,4,5-trimethoxyphenyl)prop-2-en-1-one (**5a**)

^{1}H-NMR (500 MHz, DMSO-*d*$_6$)

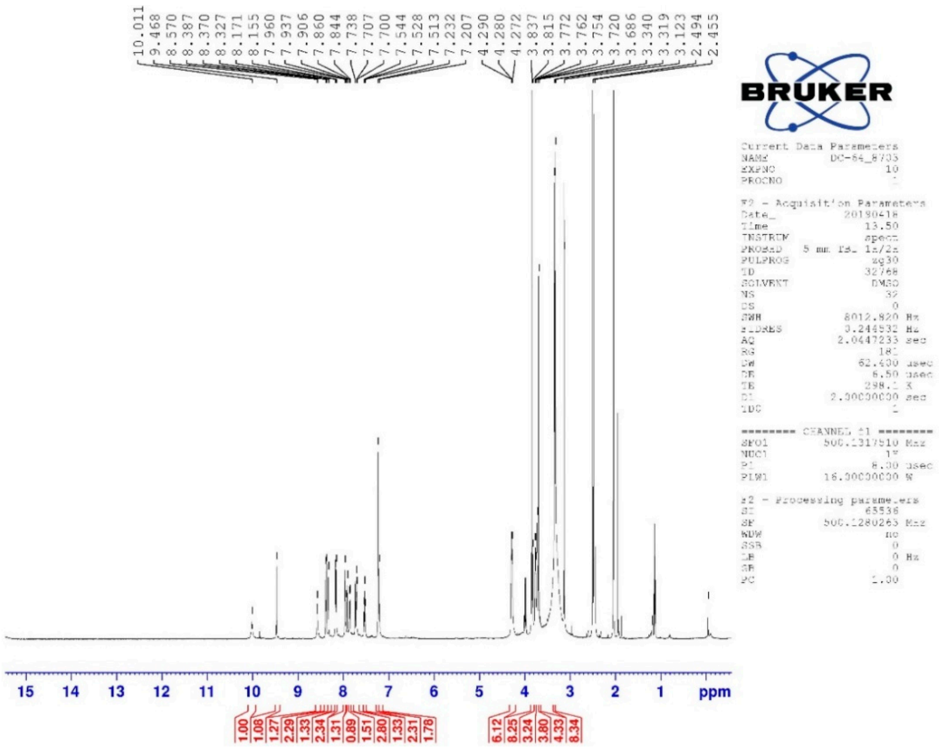

^{13}C-NMR (125 MHz, DMSO-*d*$_6$)

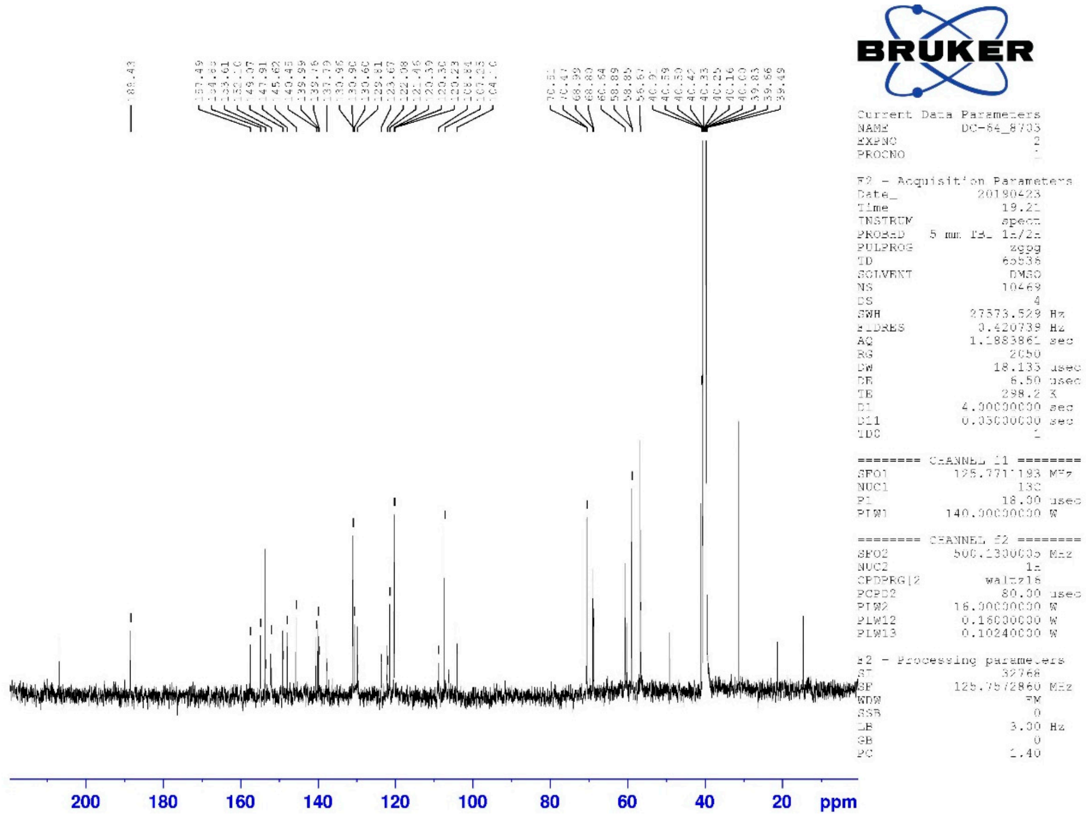

(E)-3-(4-(4-(3-((6,7-bis(2-methoxyethoxy)quinazolin-4-yl)amino)phenyl)-1H-1,2,3-triazol-1-yl)phenyl)-1-(ferrocenyl)prop-2-en-1-one (**5b**)

^{1}H-NMR (500 MHz, DMSO-*d*$_6$)

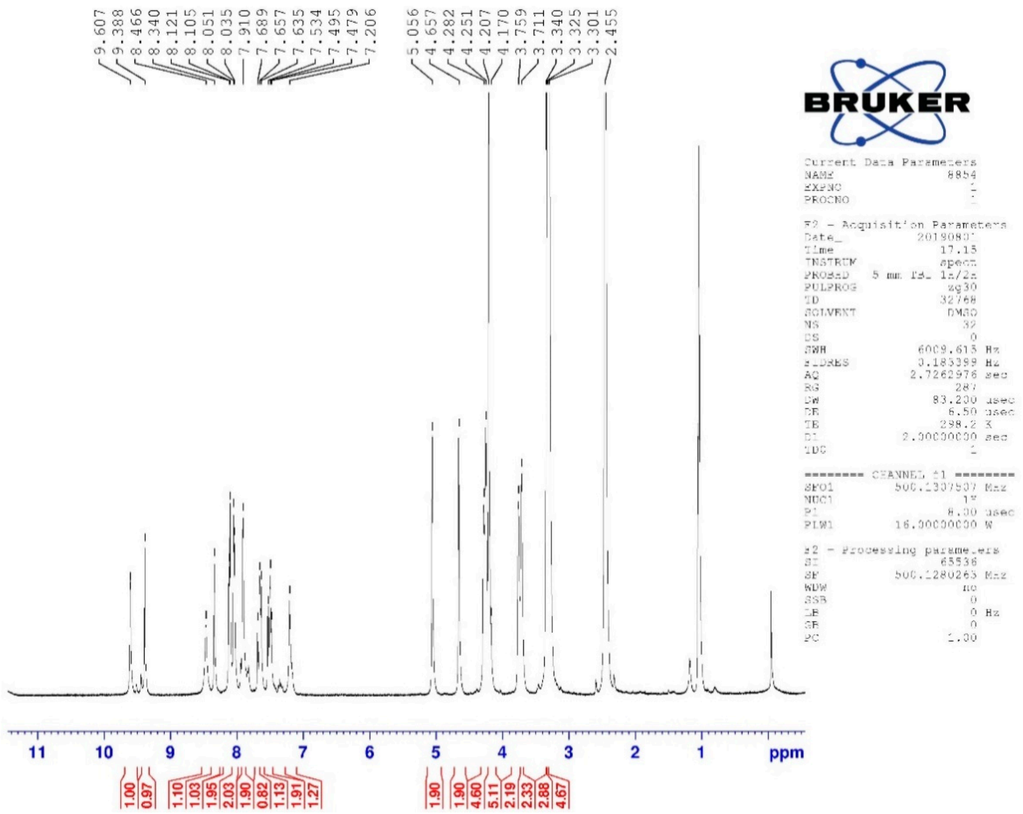

^{13}C-NMR (125 MHz, DMSO-*d*$_6$)

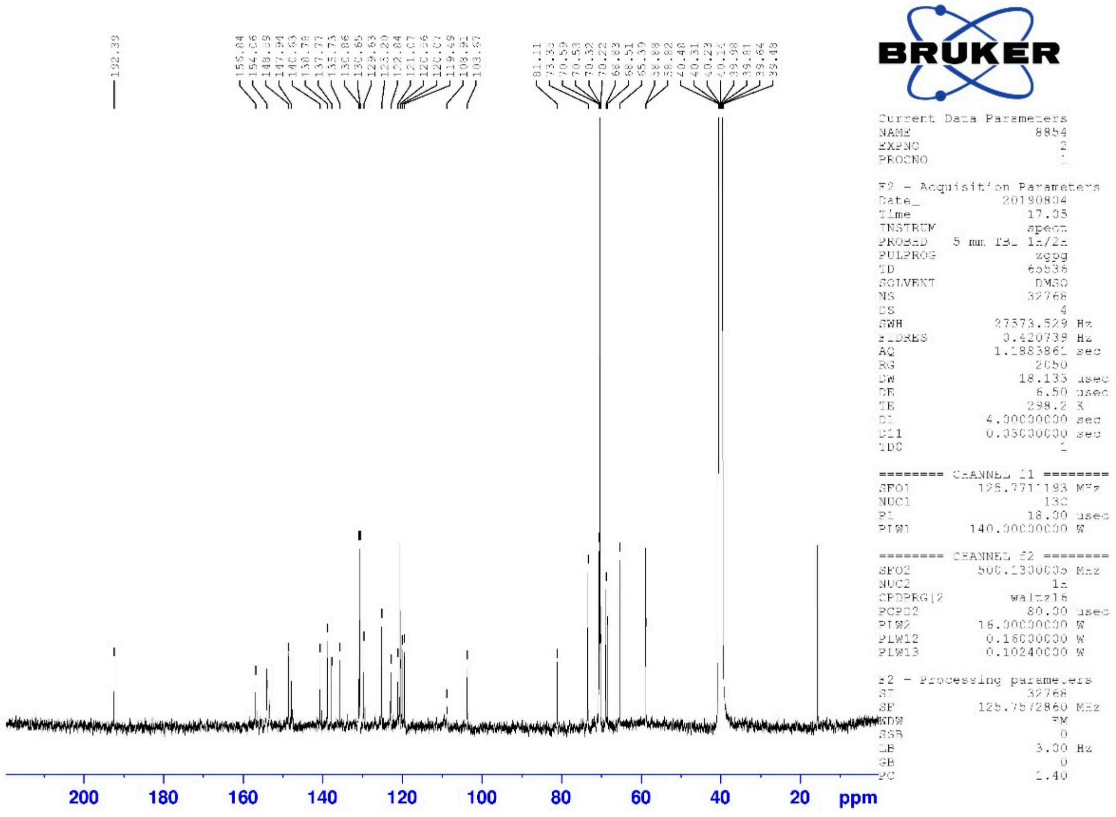

(E)-3-(4-(4-(3-((6,7-bis(2-methoxyethoxy)quinazolin-4-yl)amino)phenyl)-1H-1,2,3-triazol-1-yl)phenyl)-1-phenylprop-2-en-1-one (**5d**)

^{1}H-NMR (500 MHz, DMSO-d_6)

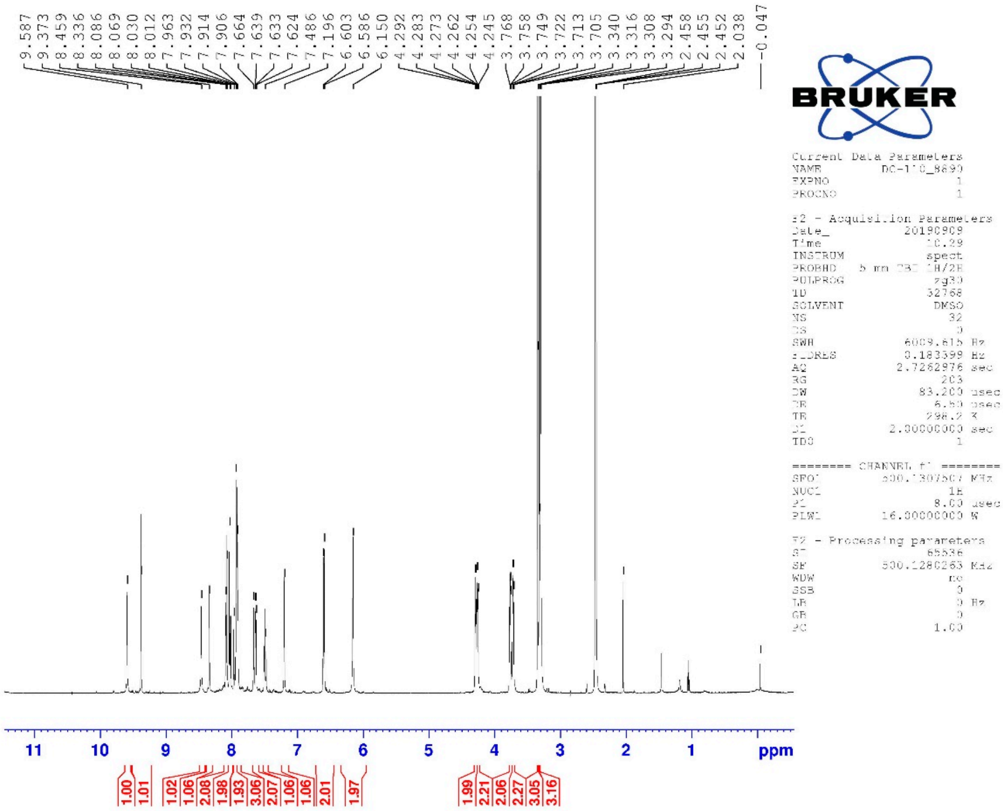

^{13}C-NMR (125 MHz, DMSO-d_6)

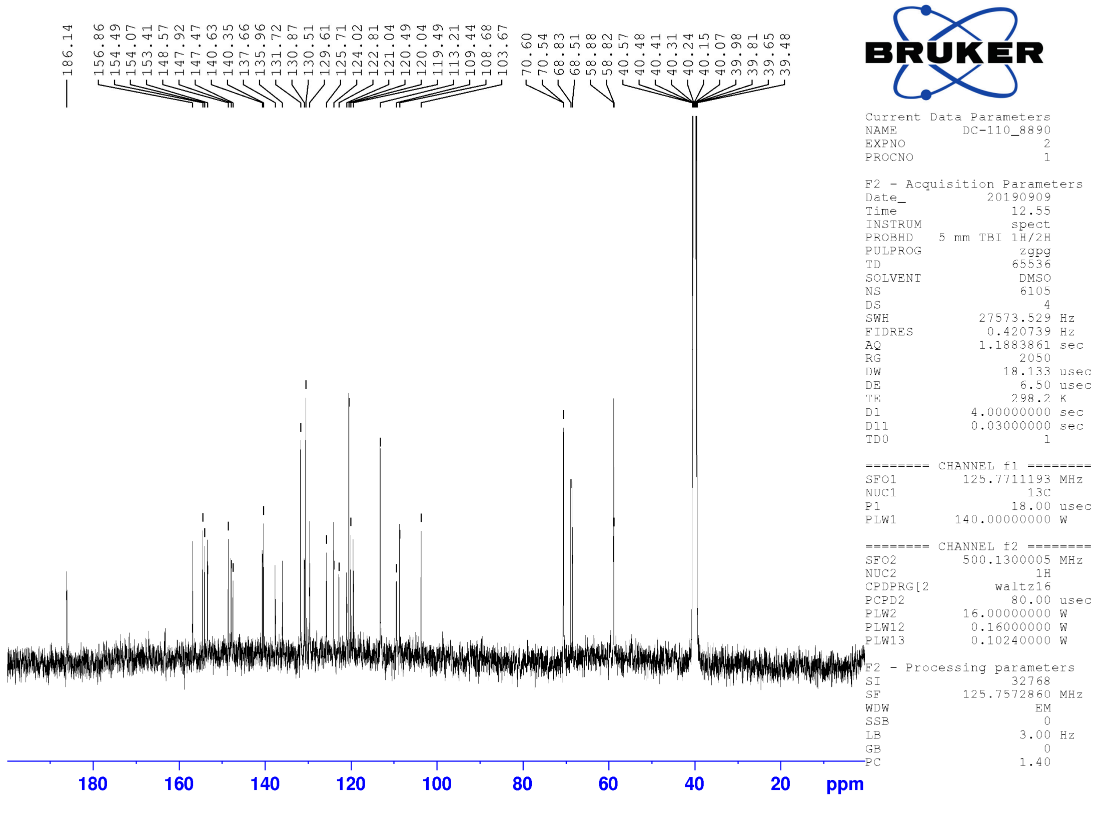

(E)-3-(2-(4-(3-((6,7-bis(2-methoxyethoxy)quinazolin-4-yl)amino)phenyl)-1H-1,2,3-triazol-1-yl)phenyl)-1-(3,4,5-trimethoxyphenyl)prop-2-en-1-one (**6a**)

^{1}H-NMR (500 MHz, DMSO-d_6)

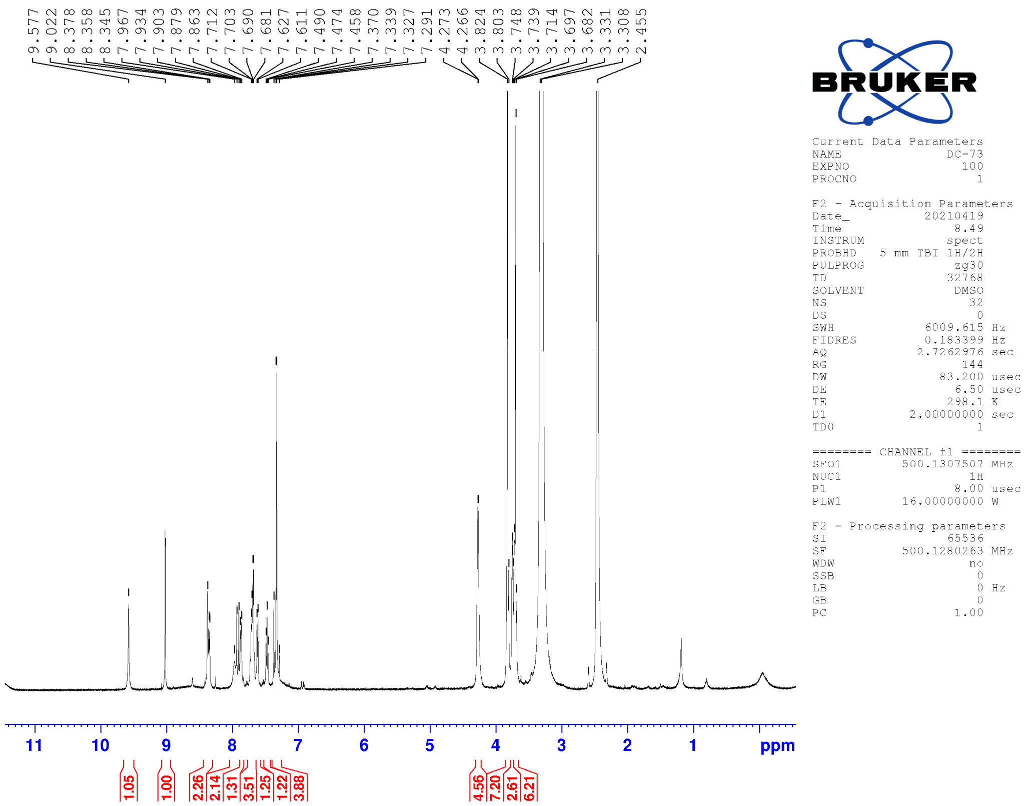

^{13}C-NMR (500 MHz, DMSO-d_6)

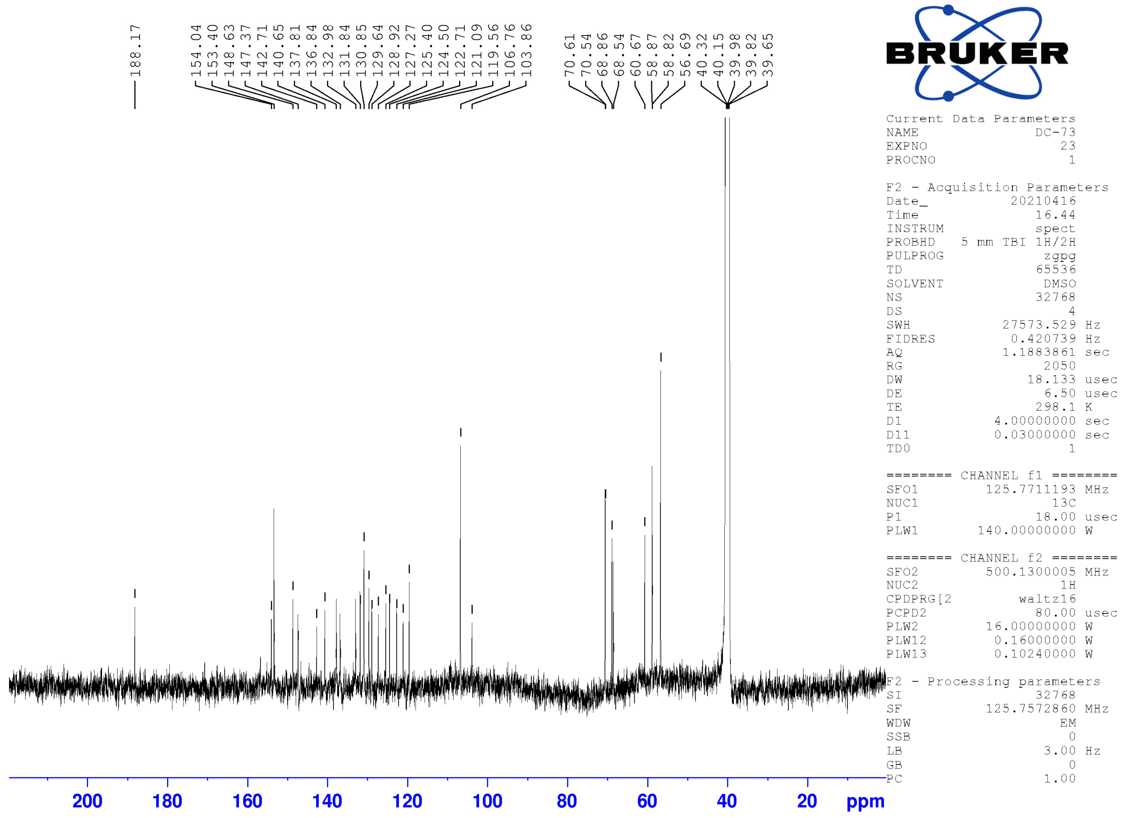

(E)-3-(2-(4-(3-((6,7-bis(2-methoxyethoxy)quinazolin-4-yl)amino)phenyl)-1H-1,2,3-triazol-1-yl)phenyl)-1-(ferrocenyl)prop-2-en-1-one (**6b**)

^{1}H-NMR (500 MHz, DMSO-d_6)

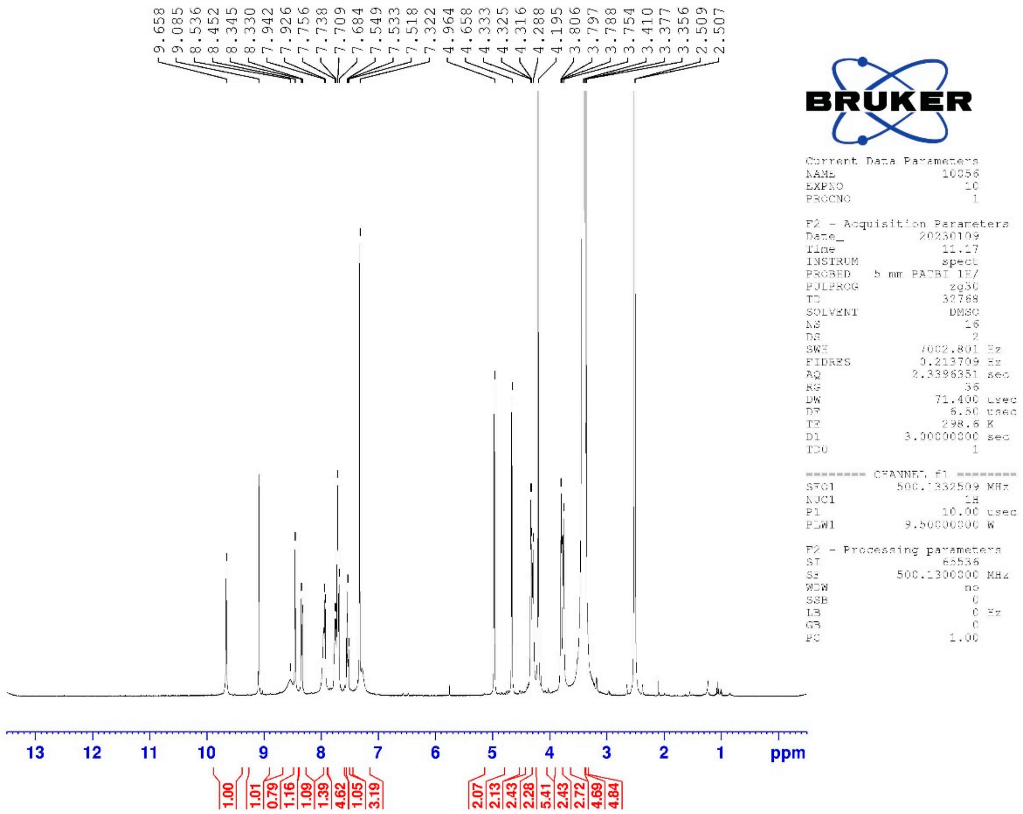

^{13}C-NMR (125 MHz, DMSO-d_6)

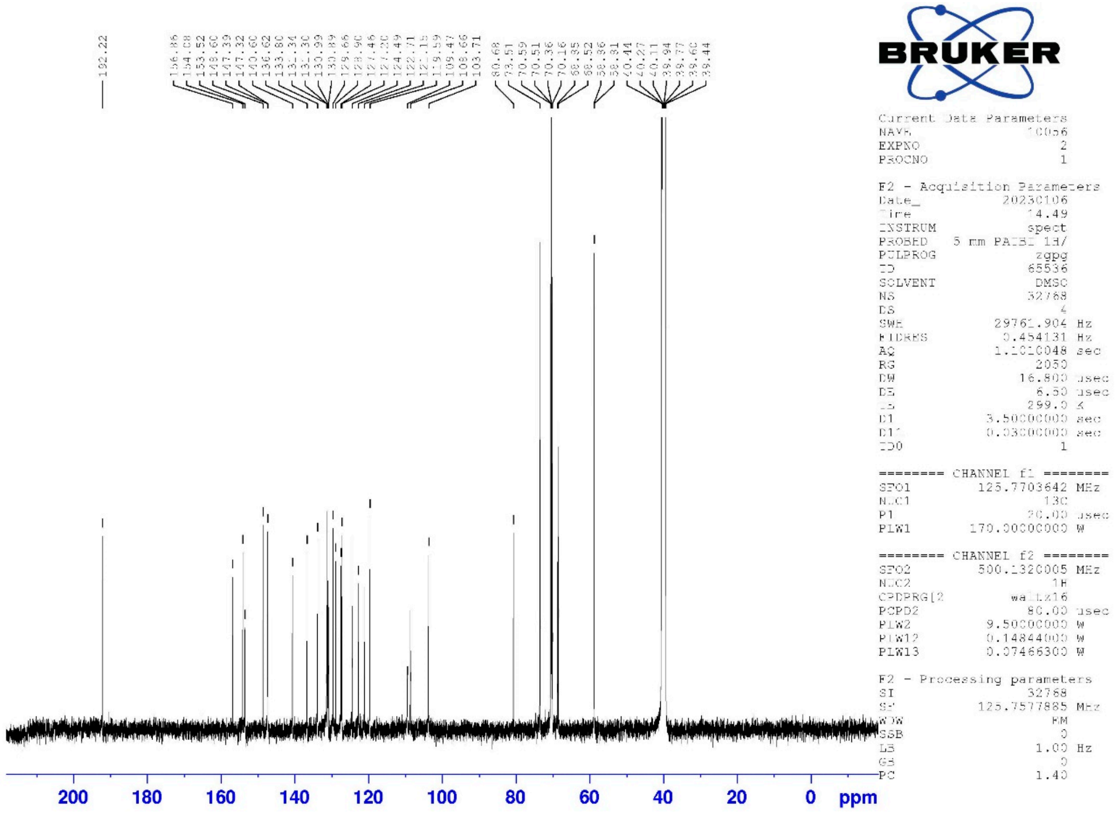

(E)-1-(4-aminophenyl)-3-(2-(4-(3-((6,7-bis(2-methoxyethoxy)quinazolin-4-yl)amino)phenyl)-1H-1,2,3-triazol-1-yl)phenyl)prop-2-en-1-one (**6d**)

^{1}H-NMR (500 MHz, DMSO-*d*6)

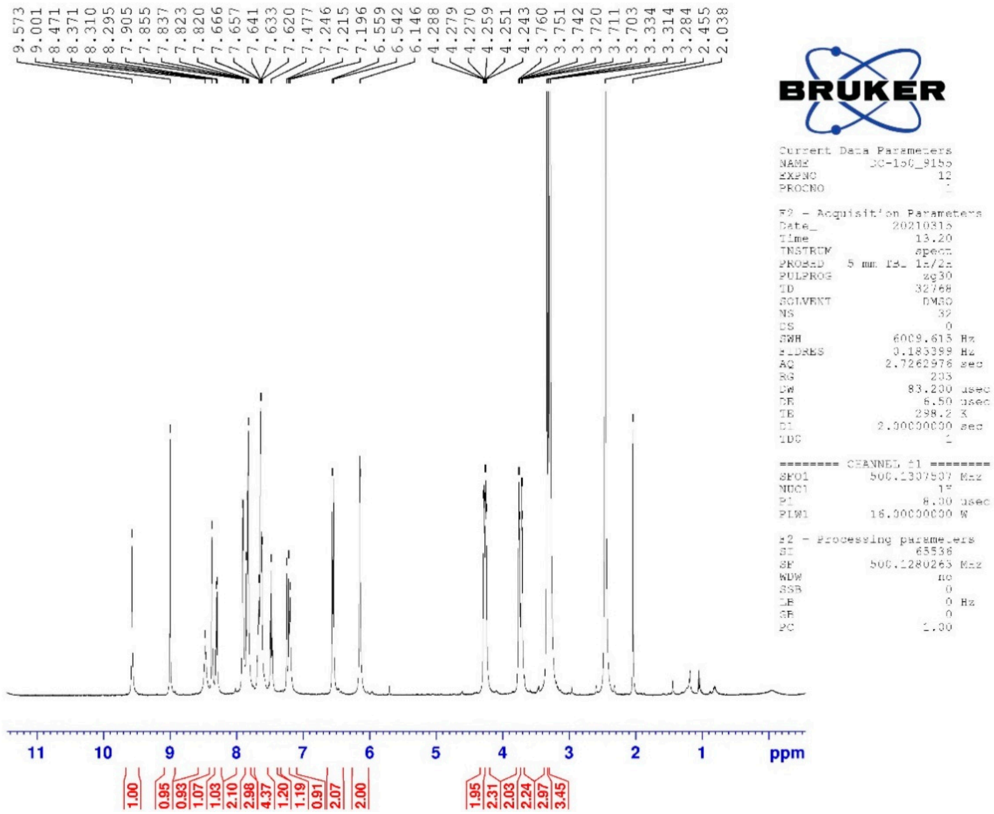

^{13}C-NMR (125 MHz, DMSO-*d*6)

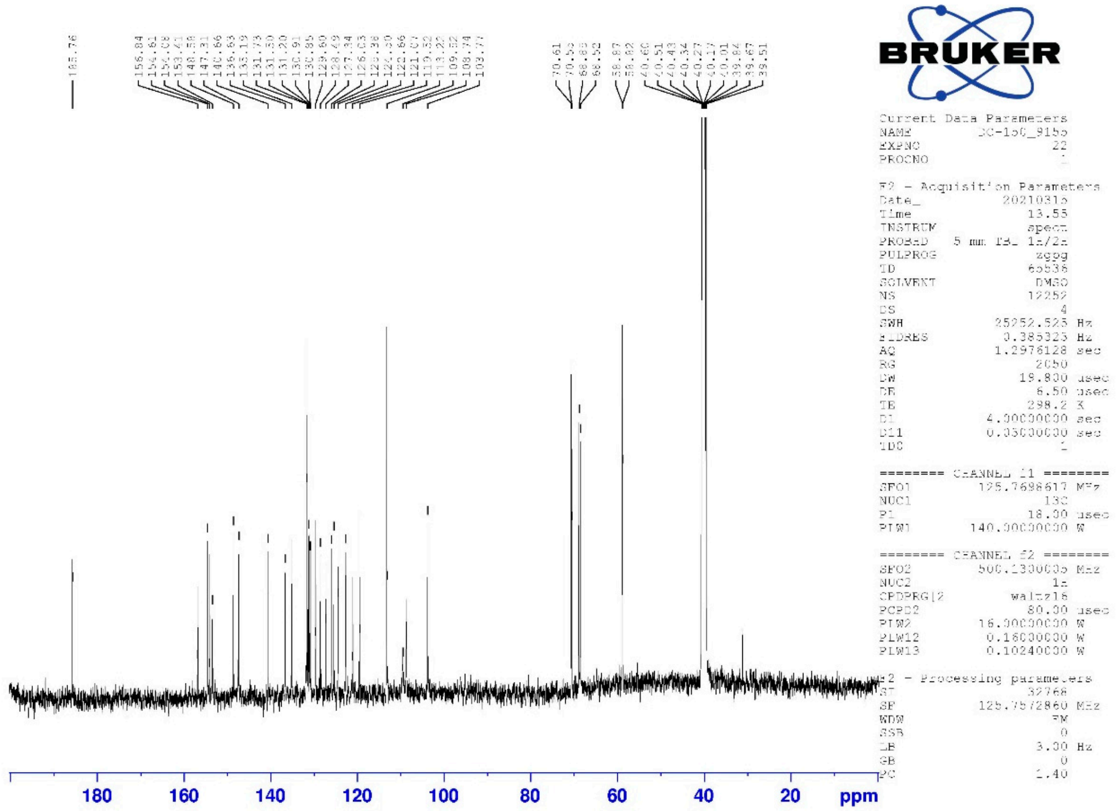

(E)-3-(4-(4-(3-((6,7-bis(2-methoxyethoxy)quinazolin-4-yl)amino)phenyl)-1H-1,2,3-triazol-1-yl)phenyl)-1-(ferrocenyl)prop-2-en-1-one (**7a**)

^{1}H-NMR (500 MHz, DMSO-*d*$_6$)

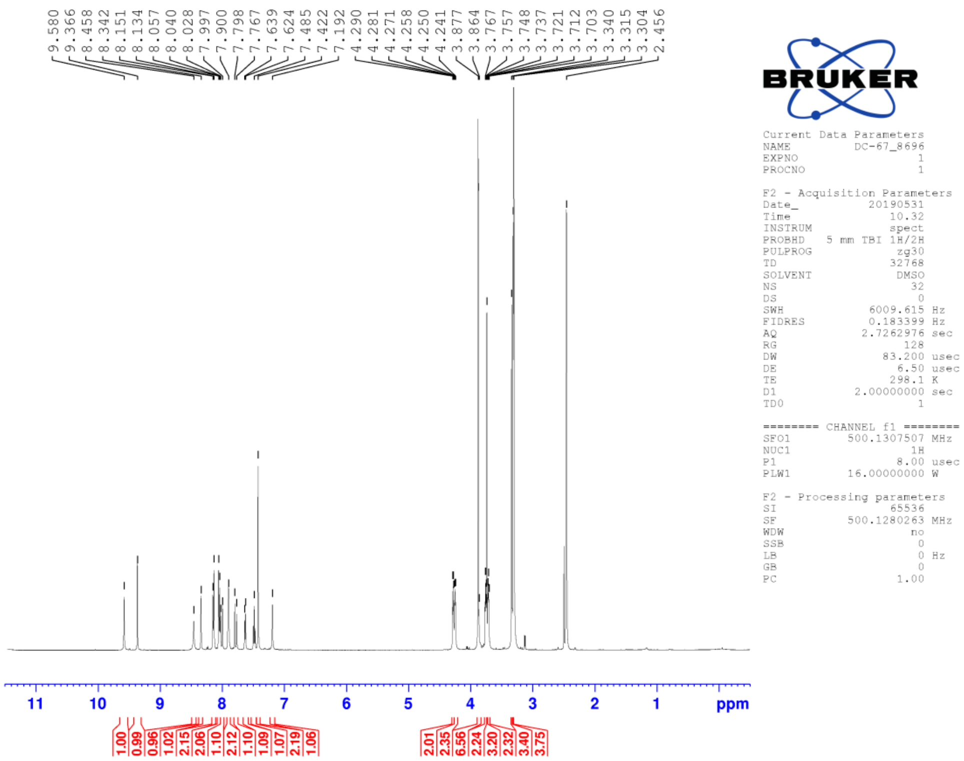

^{13}C-NMR (125 MHz, DMSO-*d*$_6$)

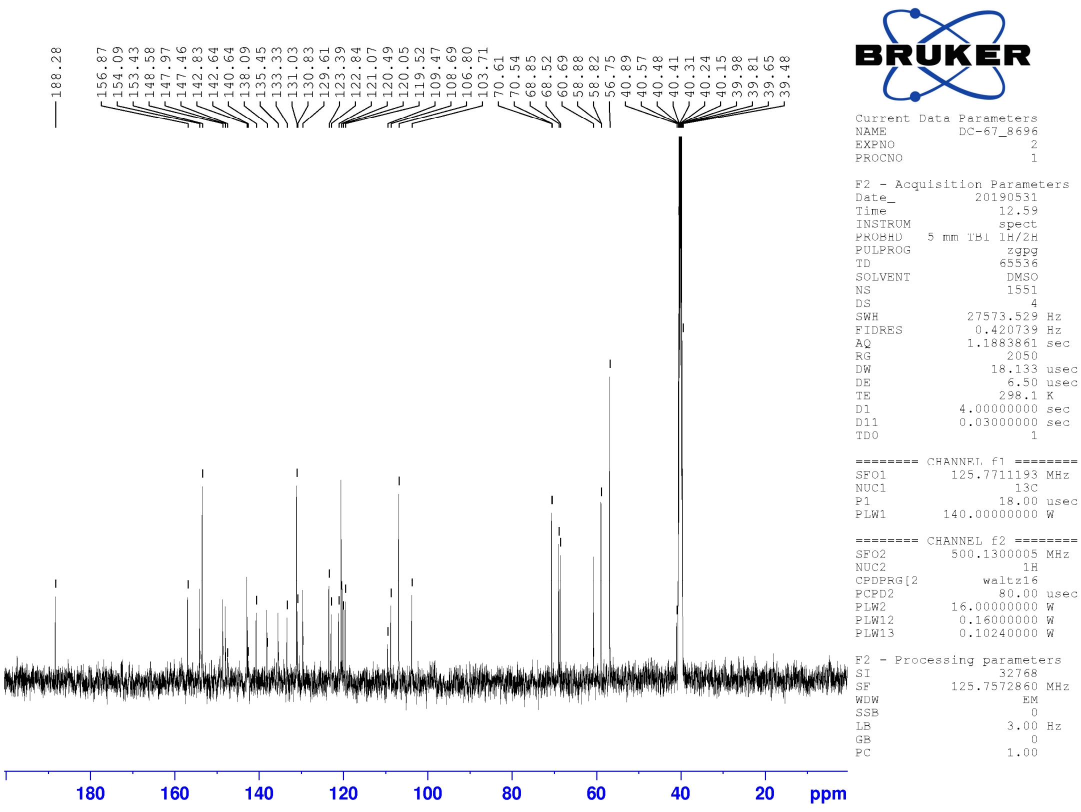

(E)-1-(4-(4-(3-((6,7-bis(2-methoxyethoxy)quinazolin-4-yl)amino)phenyl)-1H-1,2,3-triazol-1-yl)phenyl)-3-(ferrocenyl)prop-2-en-1-one (**7b**)

^{1}H-NMR (500 MHz, DMSO-d_6)

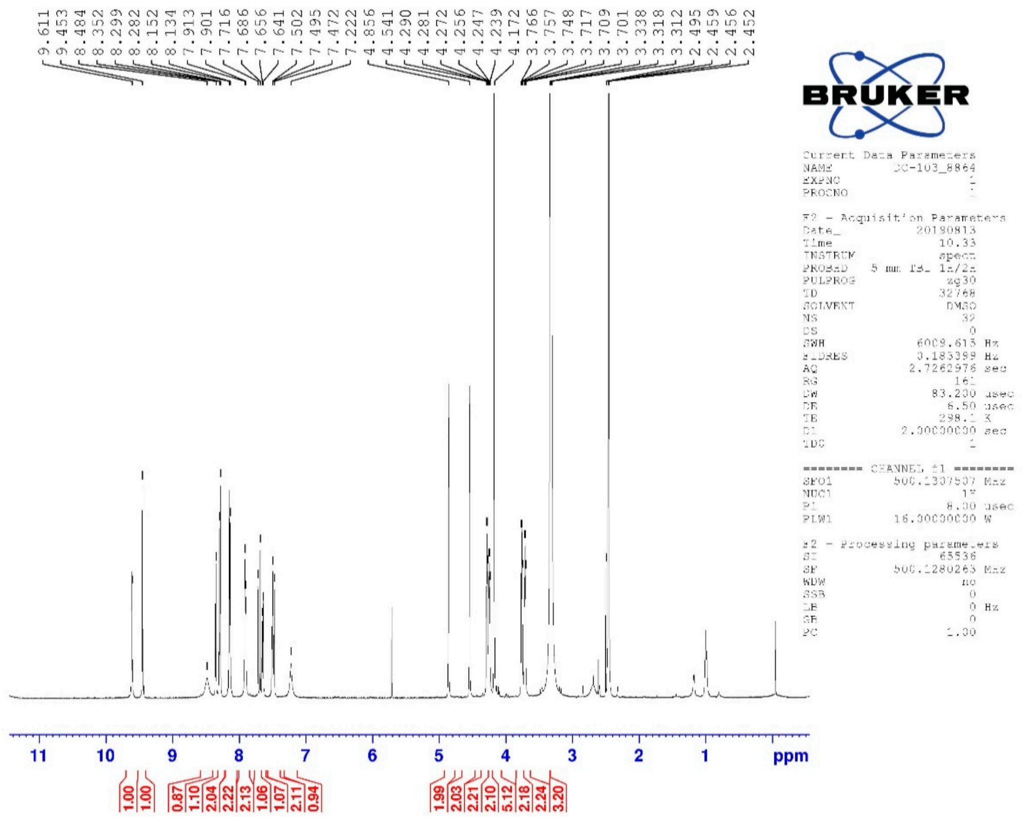

^{13}C-NMR (125 MHz, DMSO-d_6)

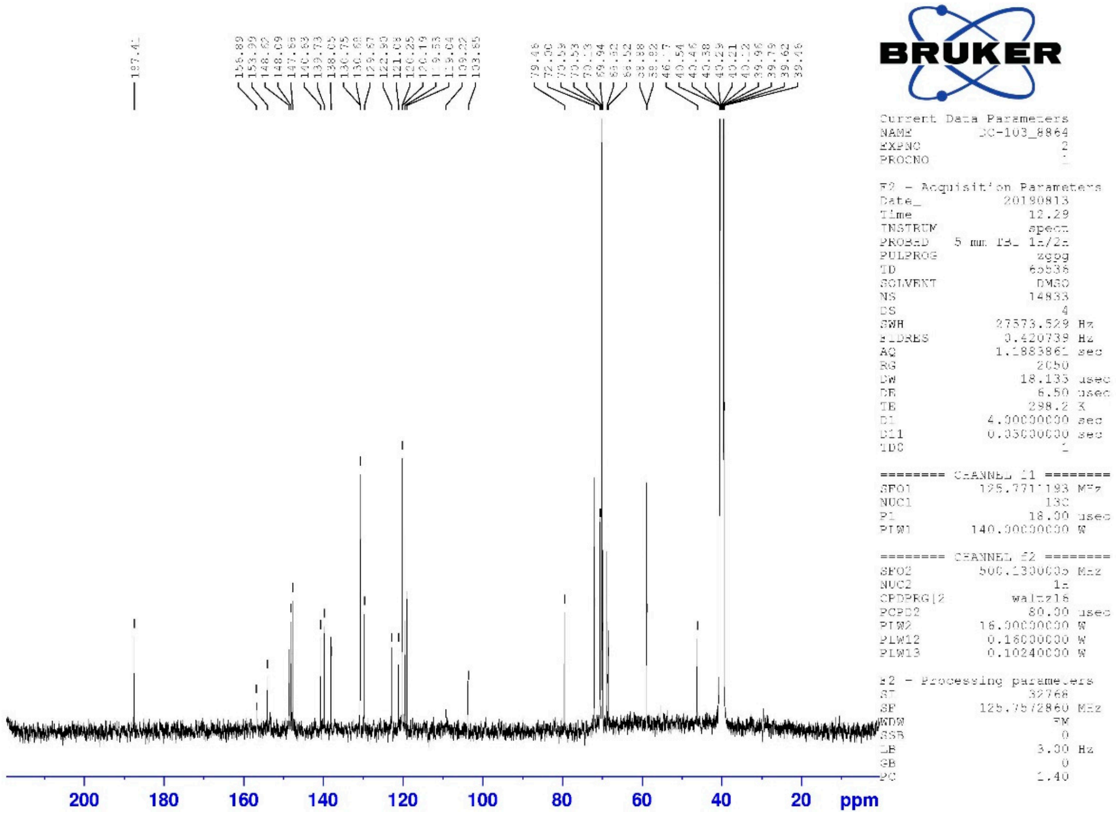

2.3 *NMR spectra of novel Erlotinib hybrids with 1,4-disubstituted alkyne linkers*

4-((3-((6,7-bis(2-methoxyethoxy)quinazolin-4-yl)amino)phenyl)ethynyl)benzaldehyde (**10**)

^{1}H-NMR (500 MHz, DMSO-d_6)

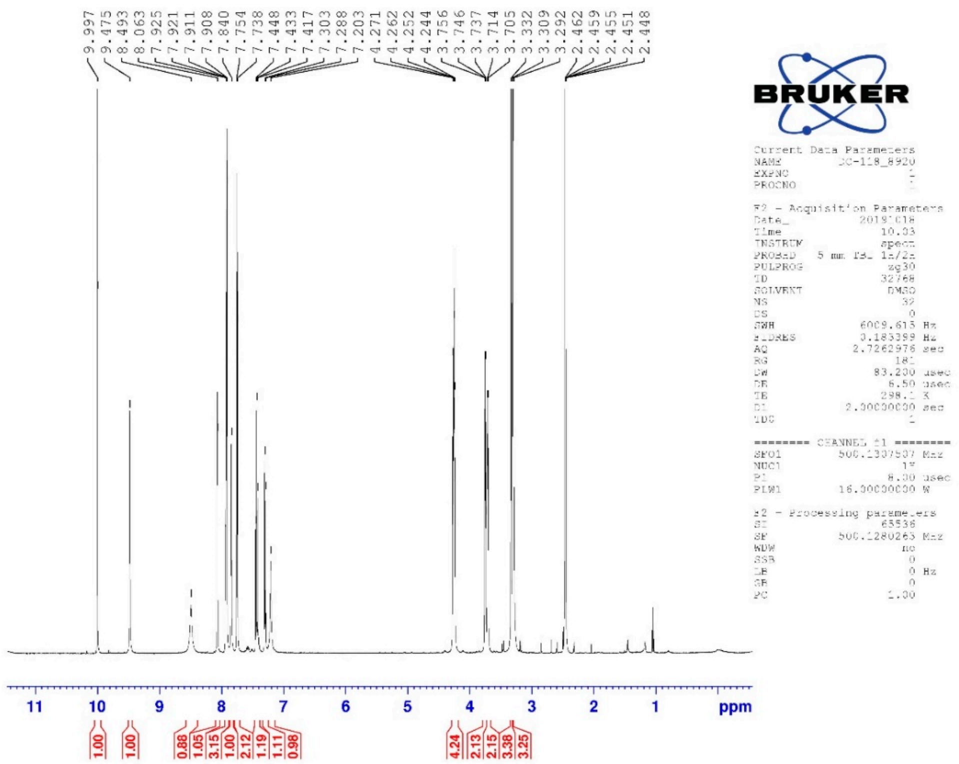

^{13}C-NMR (125 MHz, DMSO-d_6)

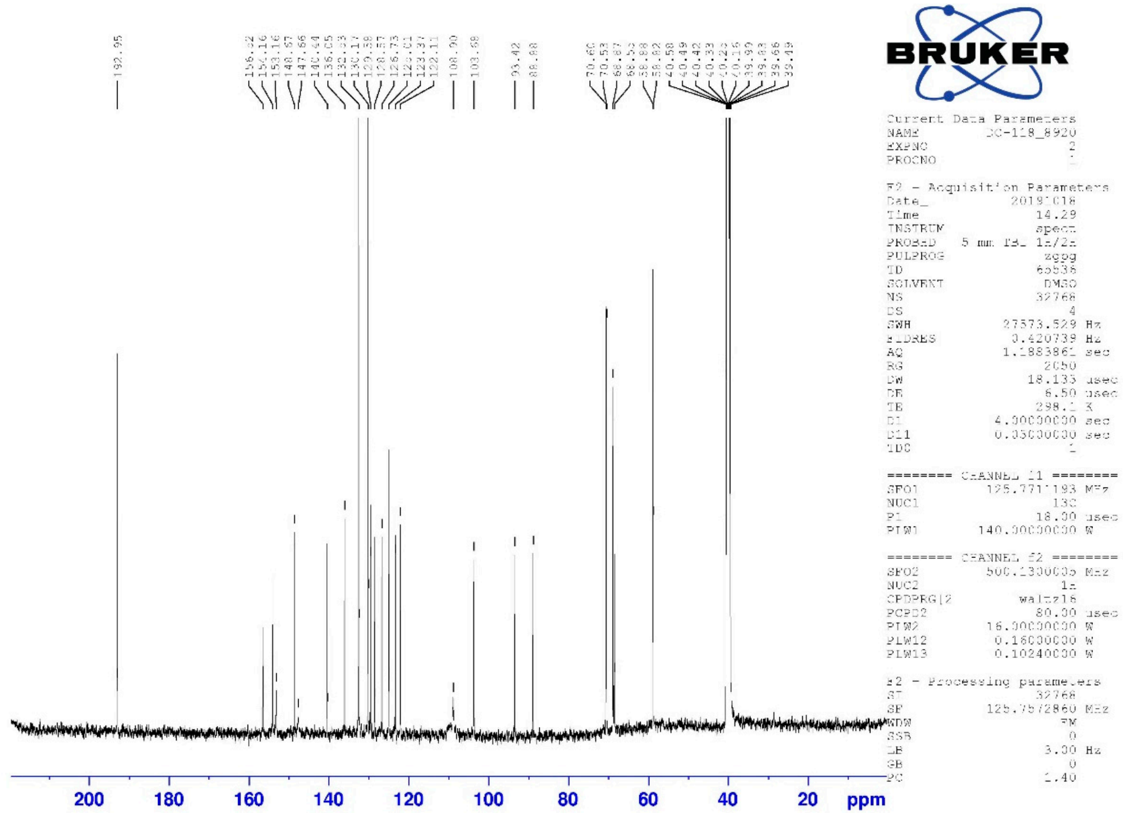

2-((3-((6,7-bis(2-methoxyethoxy)quinazolin-4-yl)amino)phenyl)ethynyl)benzaldehyde (**11**)

^{1}H-NMR (500 MHz, DMSO-*d*$_6$)

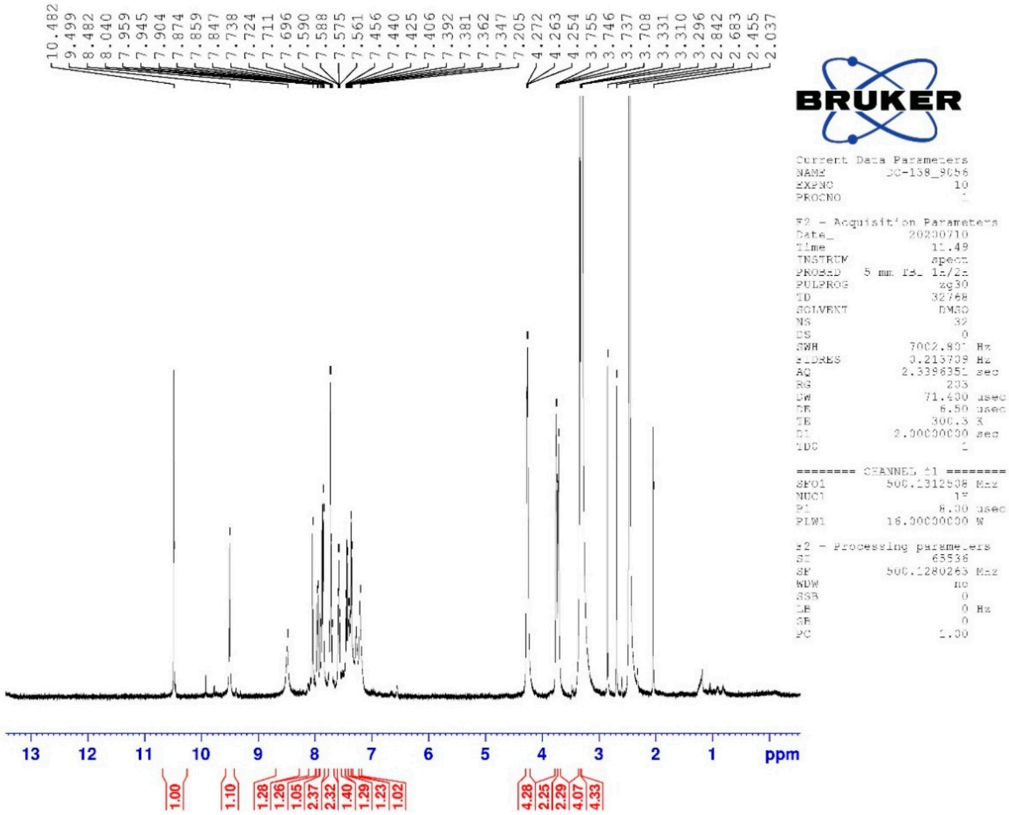

^{13}C-NMR (125 MHz, DMSO-*d*$_6$)

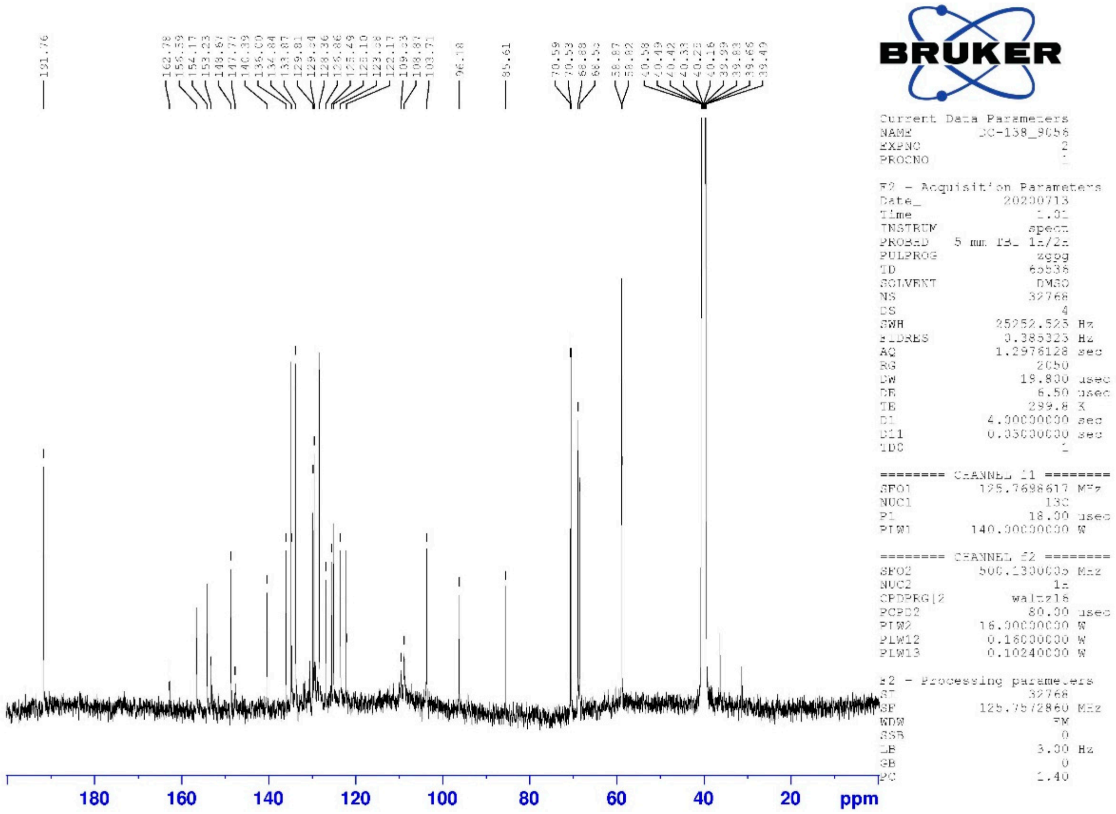

(E)-3-(4-((3-((6,7-bis(2-methoxyethoxy)quinazolin-4-yl)amino)phenyl)ethynyl)phenyl)-1-(3,4,5-trimethoxyphenyl)prop-2-en-1-one (**13**)

^{1}H-NMR (500 MHz, DMSO-d_6)

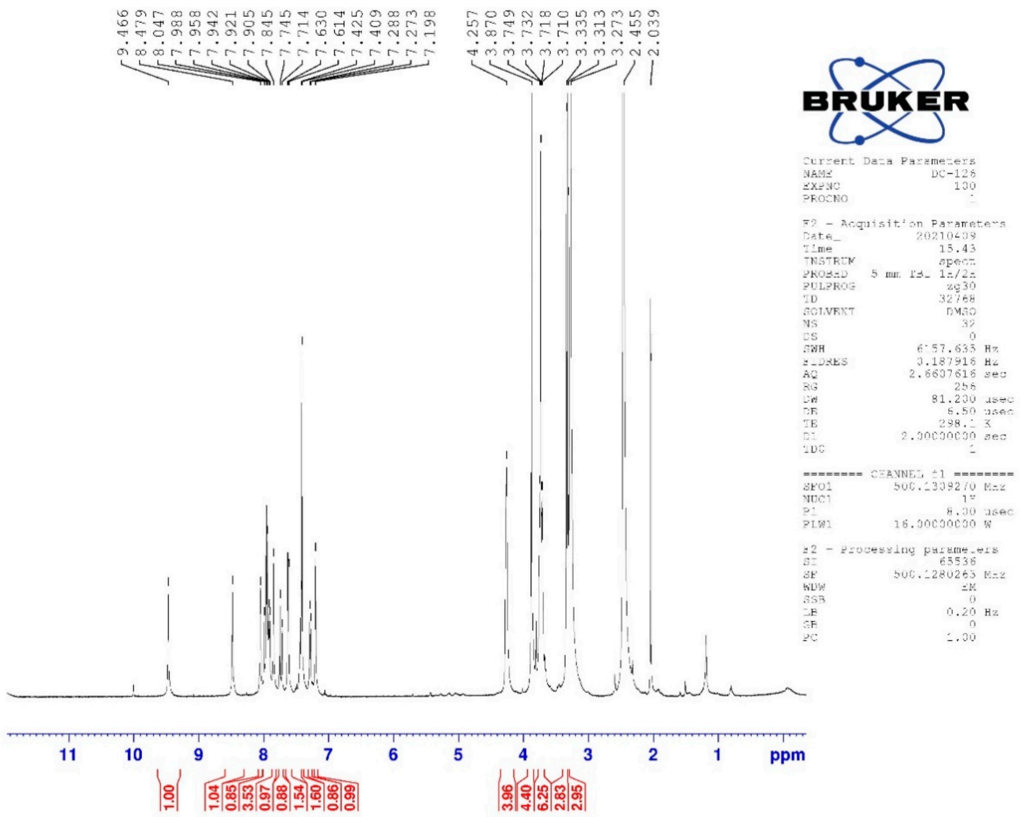

^{13}C-NMR (125 MHz, DMSO-d_6)

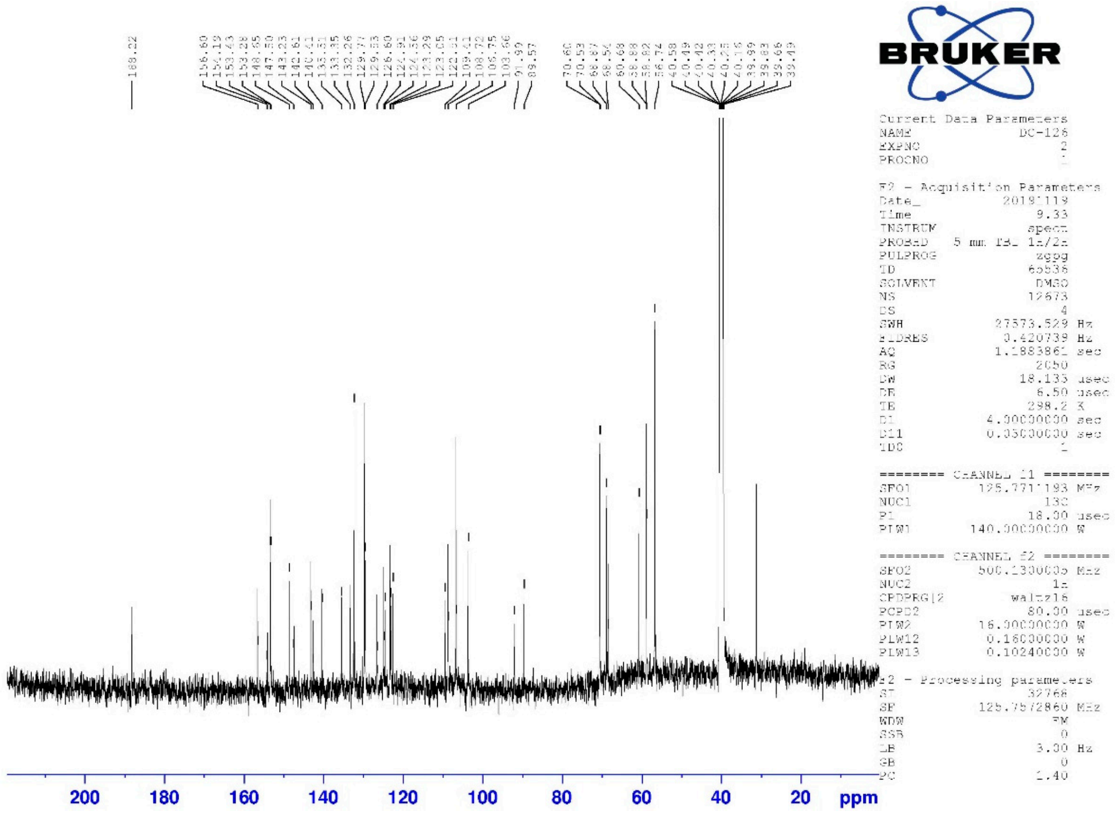

(E)-3-(2-((3-((6,7-bis(2-methoxyethoxy)quinazolin-4-yl)amino)phenyl)ethynyl)phenyl)-1-(3,4,5-trimethoxyphenyl)prop-2-en-1-one (**14**)

^{1}H-NMR (500 MHz, DMSO-d_6)

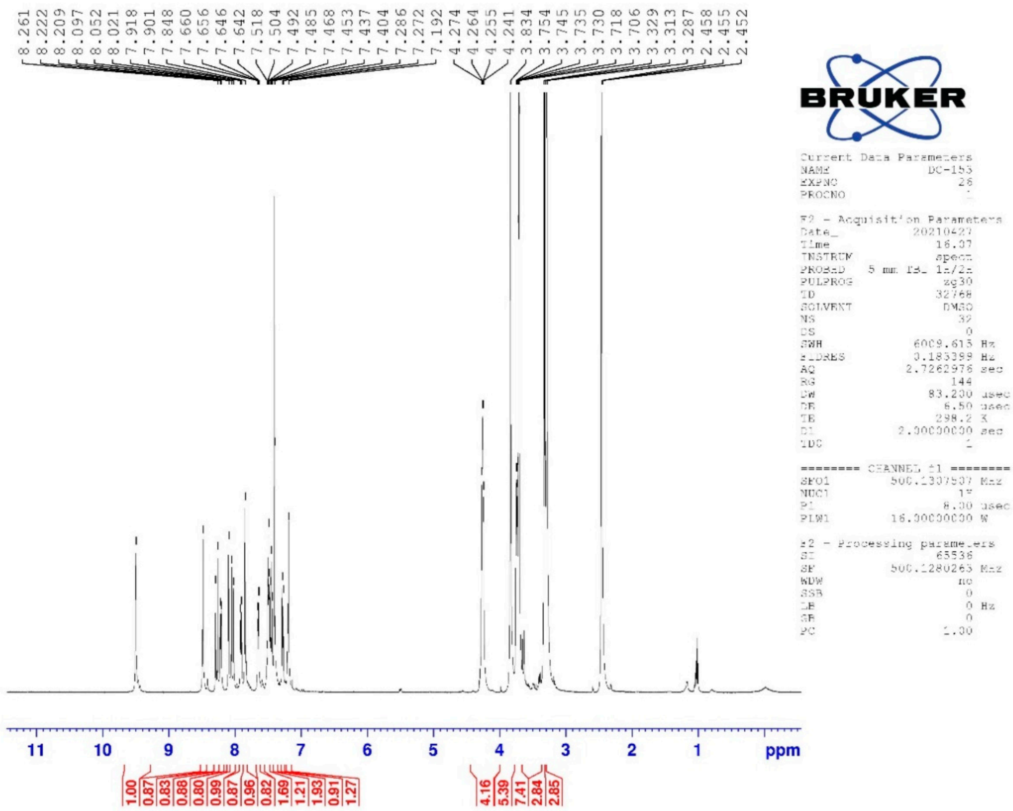

^{13}C-NMR (125 MHz, DMSO-d_6)

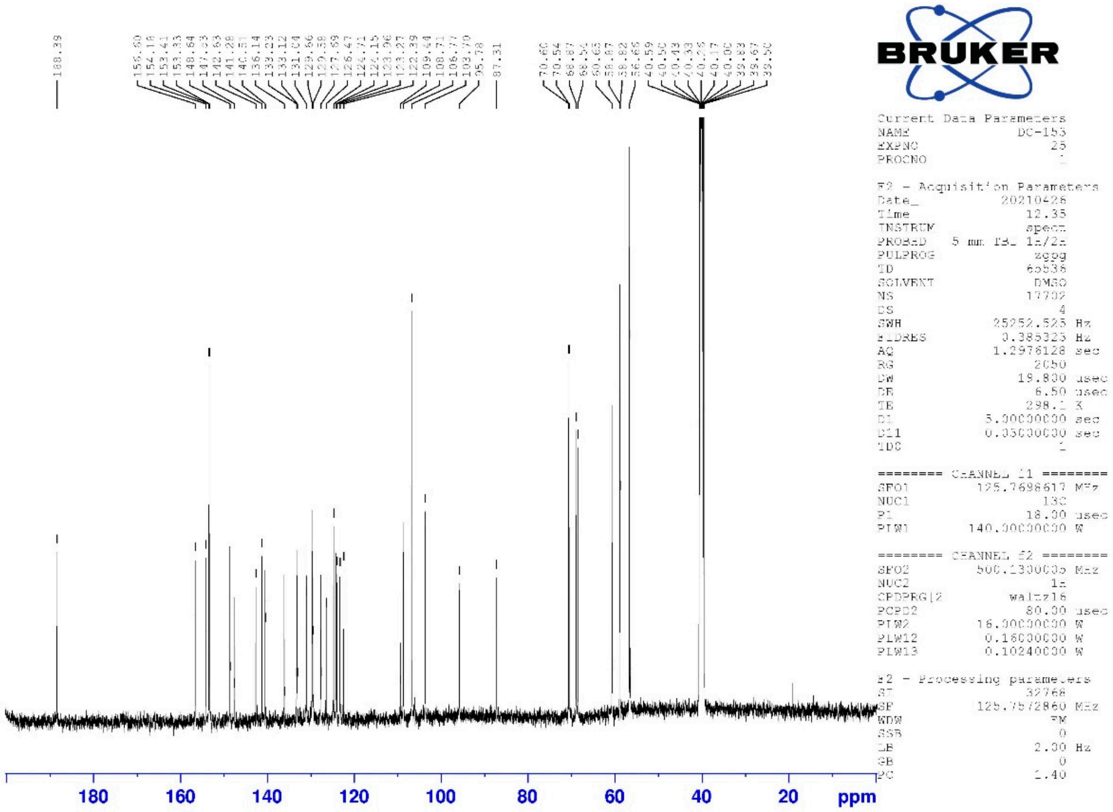

3 Copies of the HRMS spectra

3.1 *HRMS spectra of novel chalcone analogues*

Tert-butyl (E)-(4-(3-(4-azidophenyl)acryloyl)phenyl)carbamate (**2c**) (Impurity: *Tert-butyl (E)-(4-(3-(4-nitrophenyl)acryloyl)phenyl)carbamate*)	m/z calcd. for $[C_{20}H_{21}N_4O_3]^+$: 365.1614 $[M+H]^+$; found: 365.1612. mass error: 0.45 ppm (Impurity: m/z calcd. for $[C_{20}H_{21}N_2O_5]^+$: 369.1450 $[M+H]^+$; found: 369.1451. mass error: 0.14 ppm)

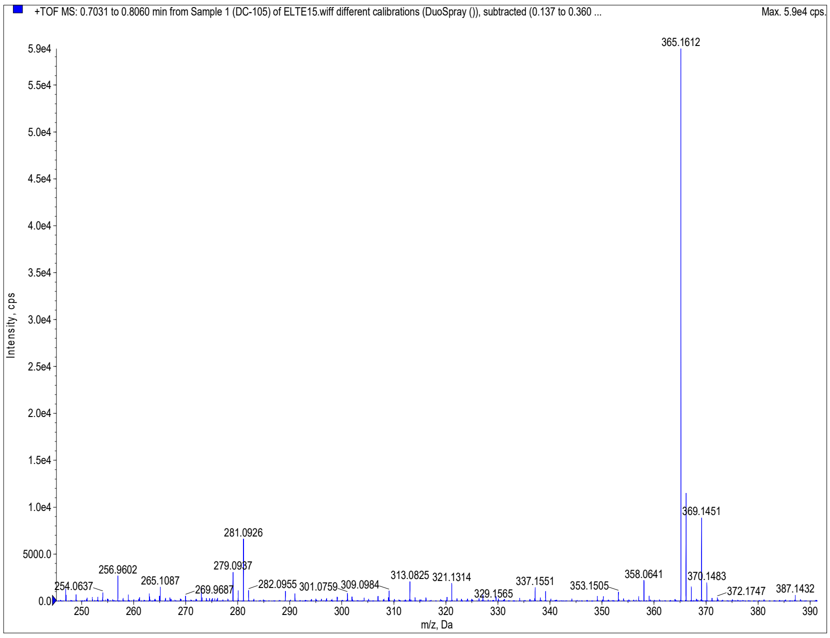

(E)-1-(4-aminophenyl)-3-(4-azidophenyl)prop-2-en-1-one (**2d**) (Impurity: *Tert-butyl (E)-(4-(3-(4-nitrophenyl)acryloyl)phenyl)carbamate*)	m/z calcd. for $[C_{15}H_{13}N_4O]^+$: 265.1089 $[M+H]^+$; found: 265.1082. mass error: 2.78 ppm (Impurity: m/z calcd. for $[C_{15}H_{13}N_2O_3]^+$: 269.0926 $[M+H]^+$; found: 269.0921. mass error: 1.92 ppm)

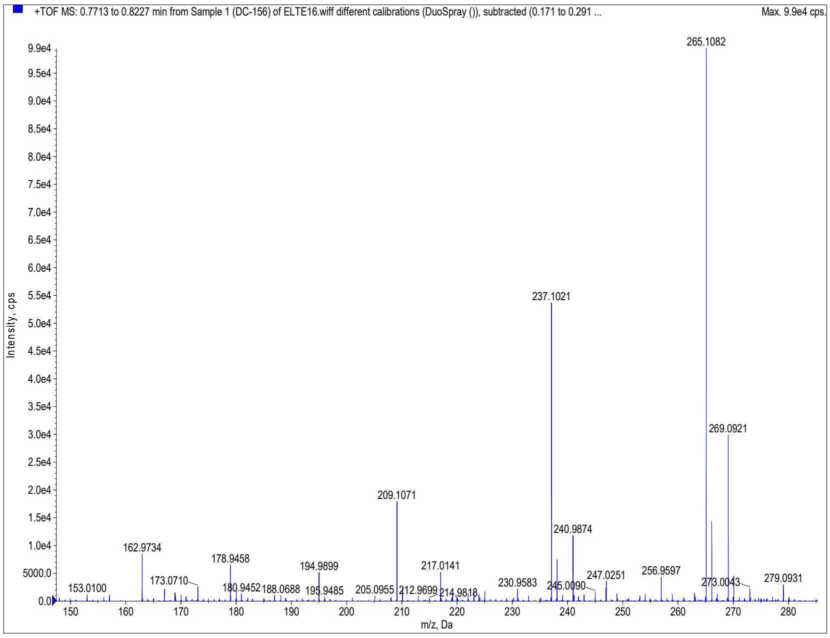

Tert-butyl (E)-(4-(3-(2-azidophenyl)acryloyl)phenyl)carbamate (**3c**)	m/z calcd. for $[C_{20}H_{21}N_4O_3]^+$: 365.1614<br>$[M+H]^+$; found: 365.1611. mass error: 0.73 ppm

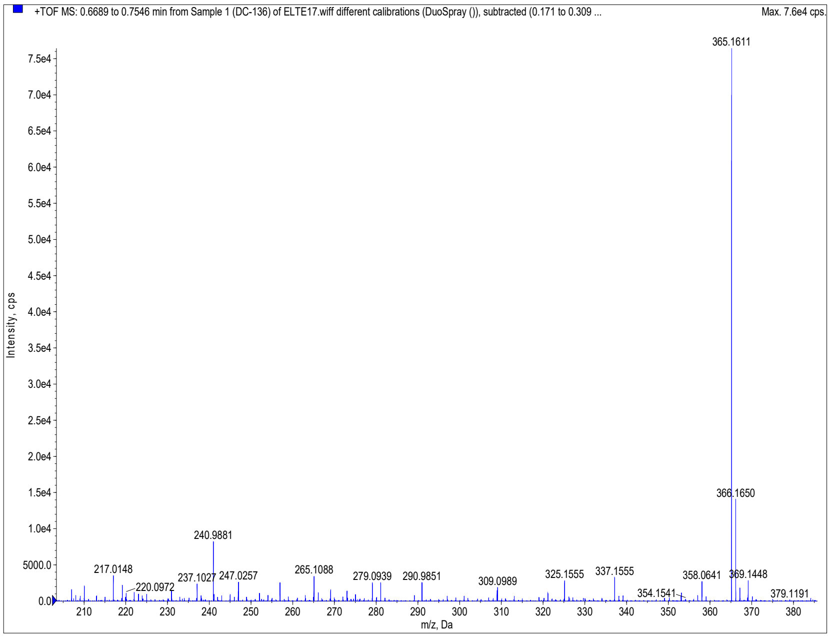

(E)-1-(4-aminophenyl)-3-(2-azidophenyl)prop-2-en-1-one (**3d**)	m/z calcd. for $[C_{15}H_{13}N_4O]^+$: 265.1089<br>$[M+H]^+$; found: 265.1082. mass error: 3.91 ppm

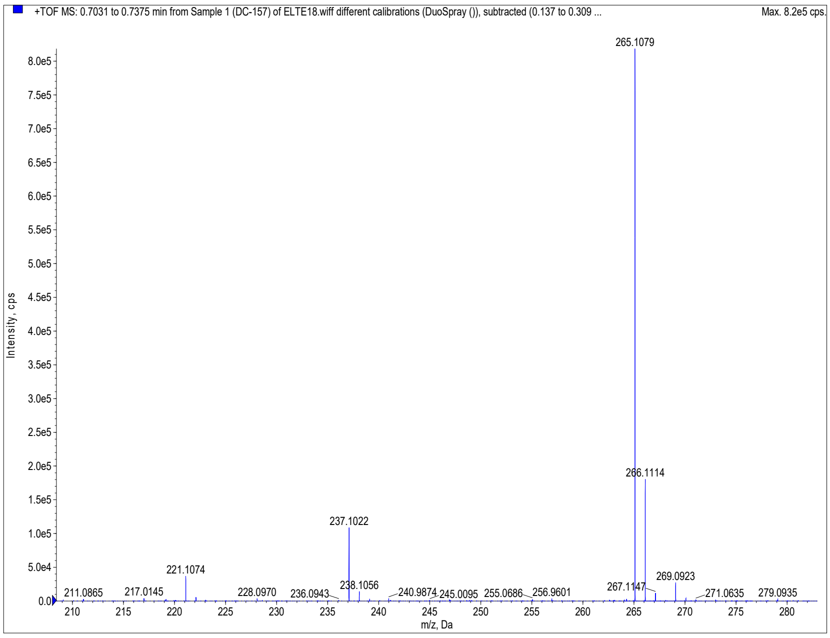

(E)-1-(4-azidophenyl)-3-(ferrocenyl)prop-2-en-1-one (**4b**)	m/z calcd. for $[C_{19}H_{16}N_3OFe]^+$: 358.0643 $[M+H]^+$; found: 358.0635. mass error: 2.17 ppm

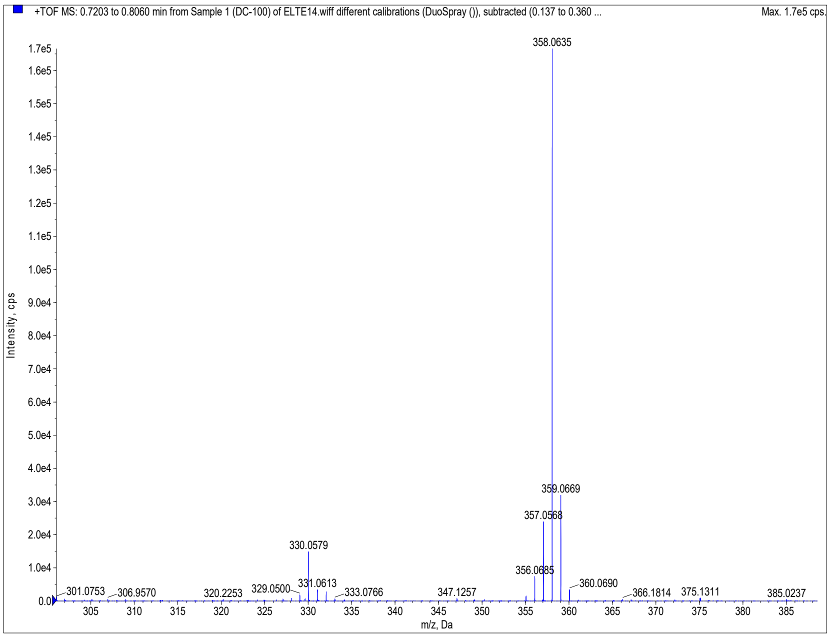

(E)-3-phenyl-1-(3,4,5-trimethoxyphenyl)prop-2-en-1-one (**15**)	m/z calcd. for $[C_{18}H_{19}O_4]^+$: 299.1283 $[M+H]^+$; found: 299.1275. mass error: 2.79 ppm

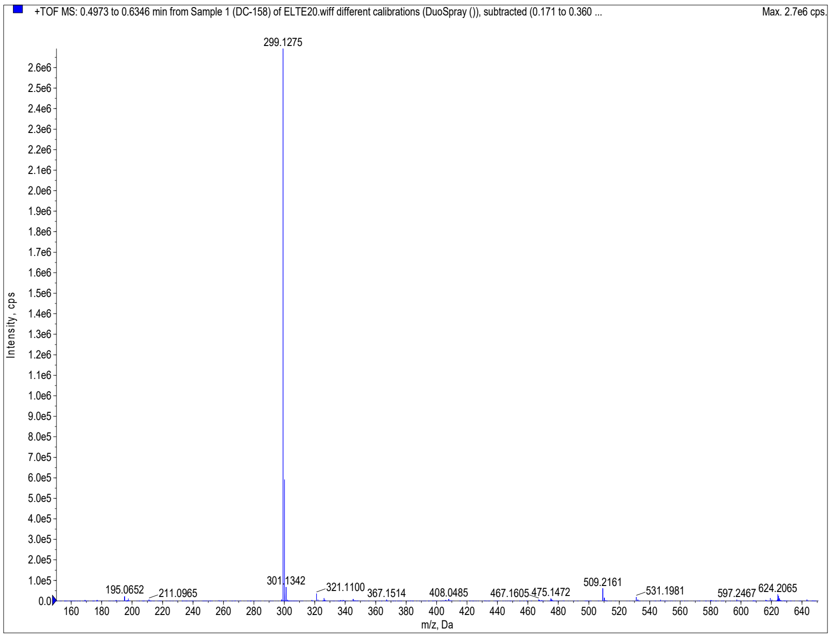

3.2 *HRMS spectra of novel Erlotinib hybrids with 1,4-disubstituted triazole linkers*

(E)-1-(4-(4-(3-((6,7-bis(2-methoxyethoxy)quinazolin-4-yl)amino)phenyl)-1H-1,2,3-triazol-1-yl)phenyl)-3-(3,4,5-trimethoxyphenyl)prop-2-en-1-one (**5a**)	m/z calcd. for [$C_{40}H_{41}N_6O_8$]: 733.2986 [M+H]$^+$; found: 733.2969. mass error: 2.30 ppm

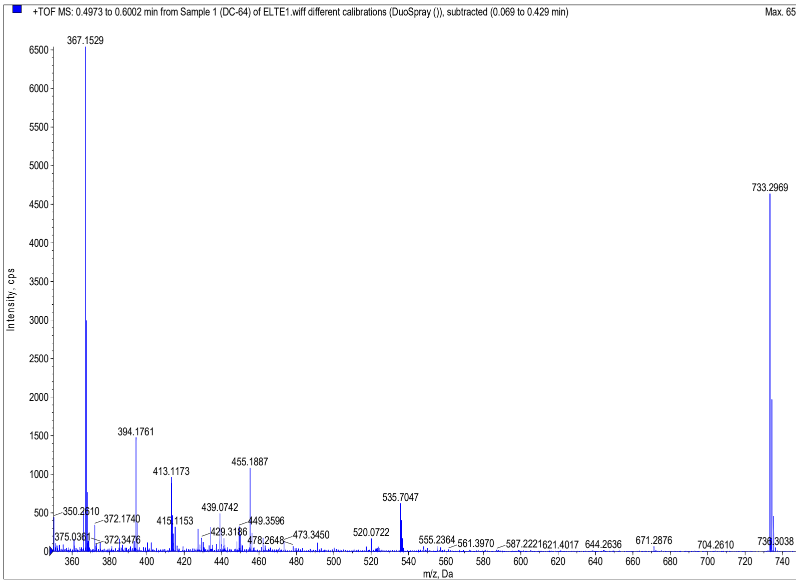

(E)-3-(4-(4-(3-((6,7-bis(2-methoxyethoxy)quinazolin-4-yl)amino)phenyl)-1H-1,2,3-triazol-1-yl)phenyl)-1-(ferrocenyl)prop-2-en-1-one (**5b**)	m/z calcd. for [$C_{41}H_{39}N_6O_5Fe$]: 751.2331 [M+H]$^+$; found: 751.2318. mass error: 1.77 ppm

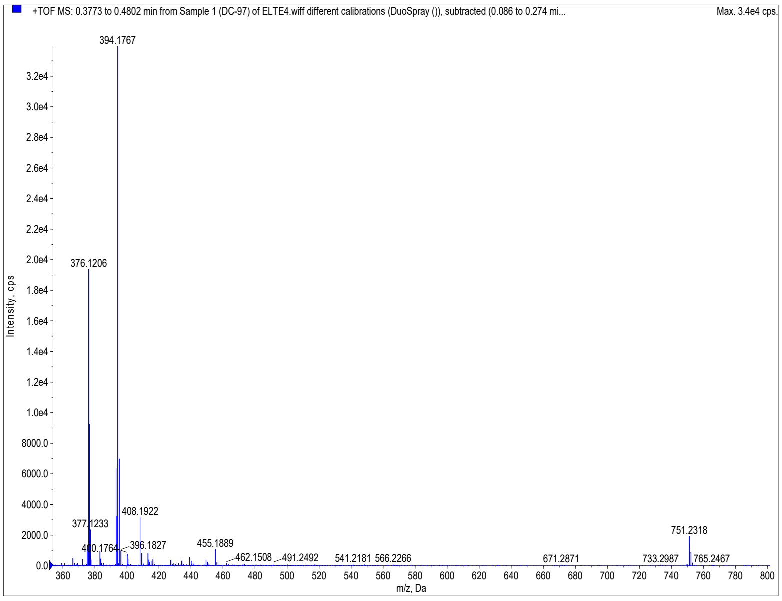

(E)-3-(4-(4-(3-((6,7-bis(2-methoxyethoxy)quinazolin-4-yl)amino)phenyl)-1H-1,2,3-triazol-1-yl)phenyl)-1-phenylprop-2-en-1-one (**5d**)	m/z calcd. for $[C_{37}H_{36}N_7O_5]$: 658.2778 $[M+H]^+$; found: 658.2754. mass error: 3.63 ppm

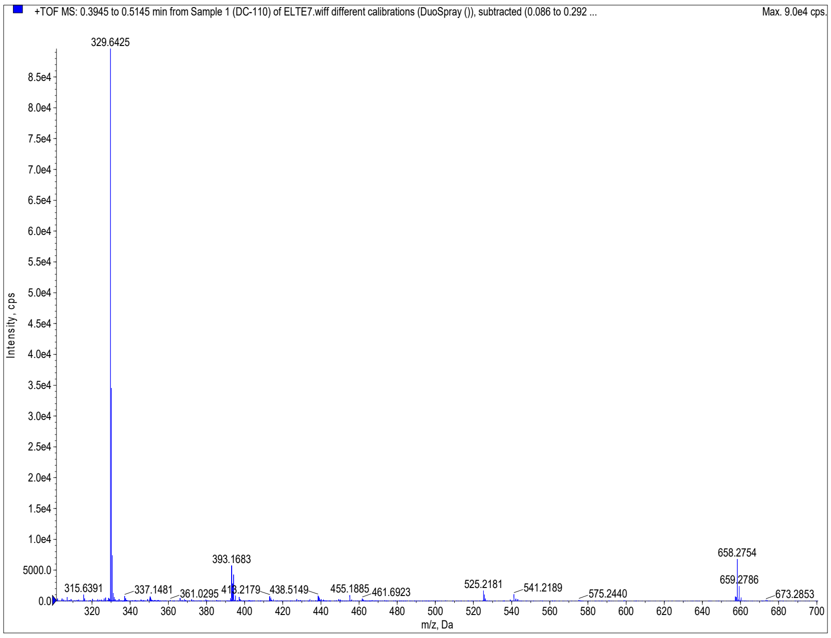

(E)-3-(2-(4-(3-((6,7-bis(2-methoxyethoxy)quinazolin-4-yl)amino)phenyl)-1H-1,2,3-triazol-1-yl)phenyl)-1-(3,4,5-trimethoxyphenyl)prop-2-en-1-one (**6a**)	m/z calcd. for $[C_{40}H_{41}N_6O_8]$: 733.2986 $[M+H]^+$; found: 733.2958. mass error: 3.80 ppm

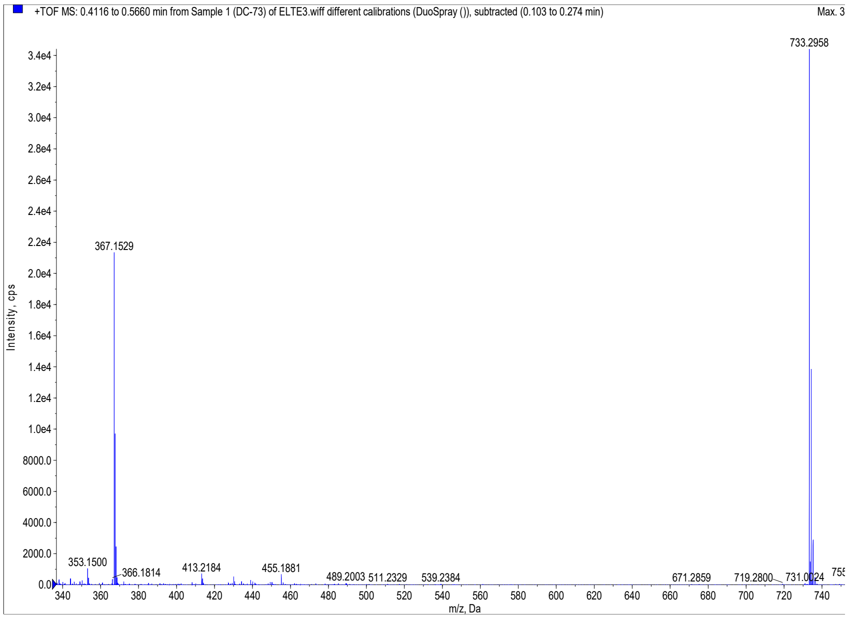

(E)-3-(2-(4-(3-((6,7-bis(2-methoxyethoxy)quinazolin-4-yl)amino)phenyl)-1H-1,2,3-triazol-1-yl)phenyl)-1-(ferrocenyl)prop-2-en-1-one (**6b**)	m/z calcd. for [$C_{41}H_{39}N_6O_5Fe$]: 751.2331 [M+H]$^+$; found: 751.2308. mass error: 3.11 ppm

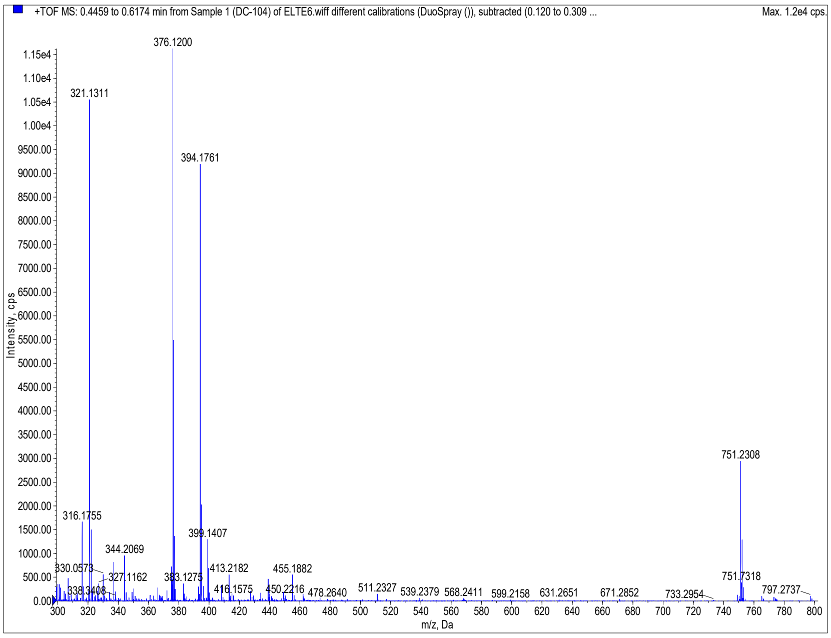

(E)-1-(4-aminophenyl)-3-(2-(4-(3-((6,7-bis(2-methoxyethoxy)quinazolin-4-yl)amino)phenyl)-1H-1,2,3-triazol-1-yl)phenyl)prop-2-en-1-one (**6d**)	m/z calcd. for [$C_{37}H_{36}N_7O_5$]: 658.2778 [M+H]$^+$; found: 658.2750. mass error: 4.24 ppm

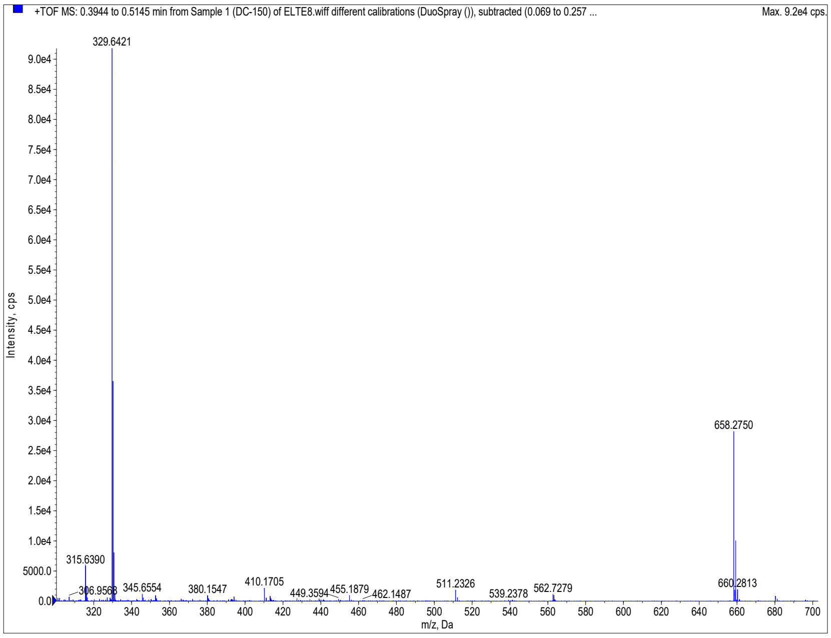

(E)-3-(4-(4-(3-((6,7-bis(2-methoxyethoxy)quinazolin-4-yl)amino)phenyl)-1H-1,2,3-triazol-1-yl)phenyl)-1-(3,4,5-trimethoxyphenyl)prop-2-en-1-one (**7a**)	m/z calcd. for [$C_{40}H_{41}N_6O_8$]: 733.2986 [M+H]$^+$; found: 733.2963. mass error: 3.12 ppm

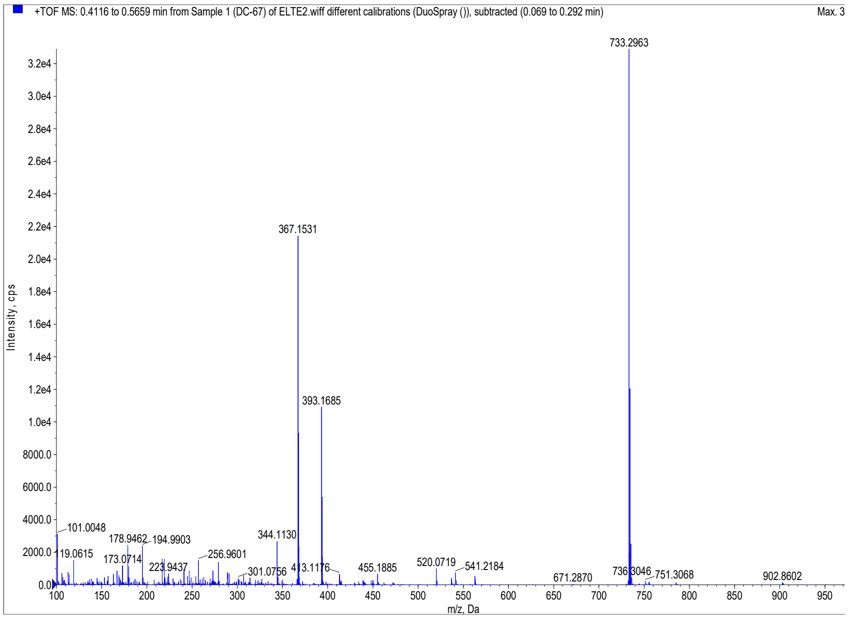

(E)-1-(4-(4-(3-((6,7-bis(2-methoxyethoxy)quinazolin-4-yl)amino)phenyl)-1H-1,2,3-triazol-1-yl)phenyl)-3-(ferrocenyl)prop-2-en-1-one (**7b**)	m/z calcd. for [$C_{41}H_{39}N_6O_5Fe$]: 751.2331 [M+H]$^+$; found: 751.2303. mass error: 3.77 ppm

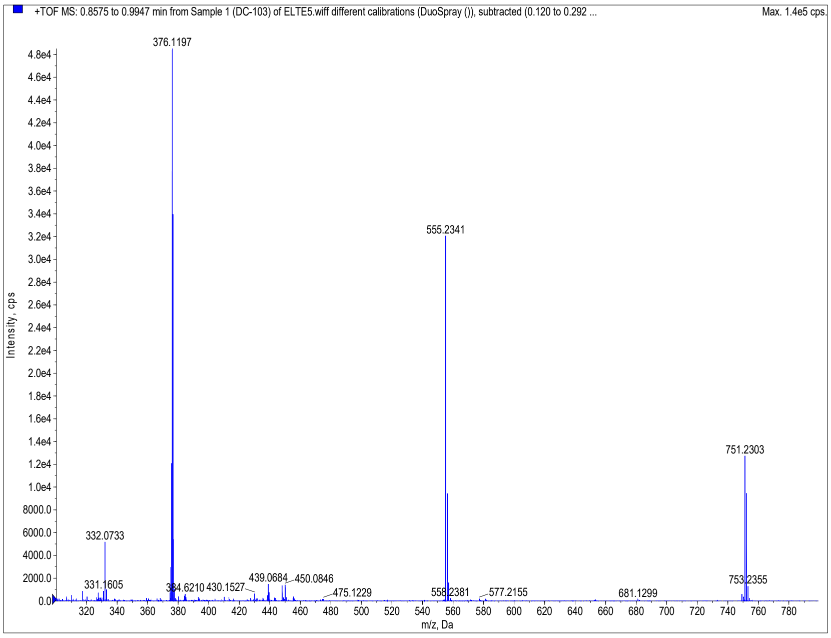

3.3 *HRMS spectra of novel Erlotinib hybrids with alkyne linkers*

4-((3-((6,7-bis(2-methoxyethoxy)quinazolin-4-yl)amino)phenyl)ethynyl)benzaldehyde (**10**)	m/z calcd. for $[C_{29}H_{28}N_3O_5]$: 498.2029 $[M+H]^+$; found: 498.2014. mass error: 3.00 ppm

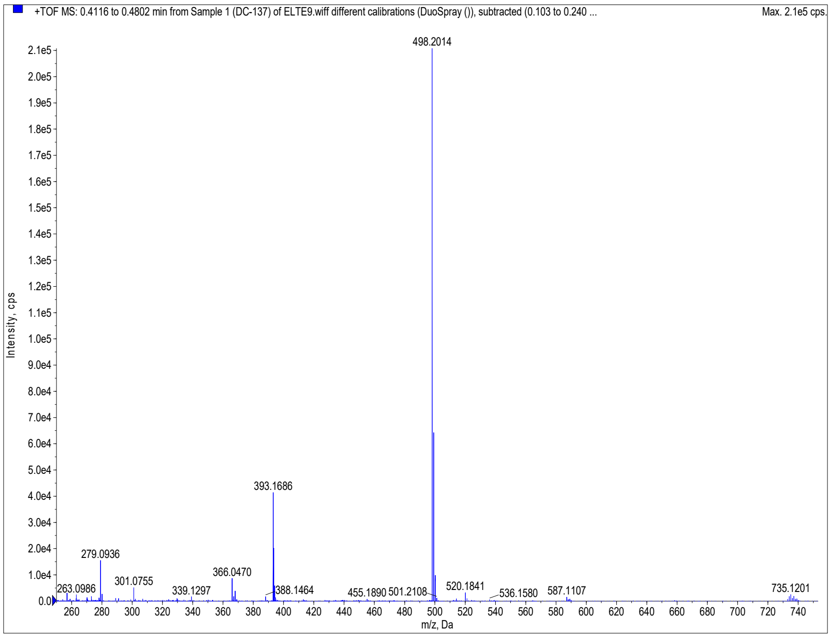

2-((3-((6,7-bis(2-methoxyethoxy)quinazolin-4-yl)amino)phenyl)ethynyl)benzaldehyde (**11**)	m/z calcd. for $[C_{29}H_{28}N_3O_5]$: 498.2029 $[M+H]^+$; found: 498.2008. mass error: 4.21 ppm

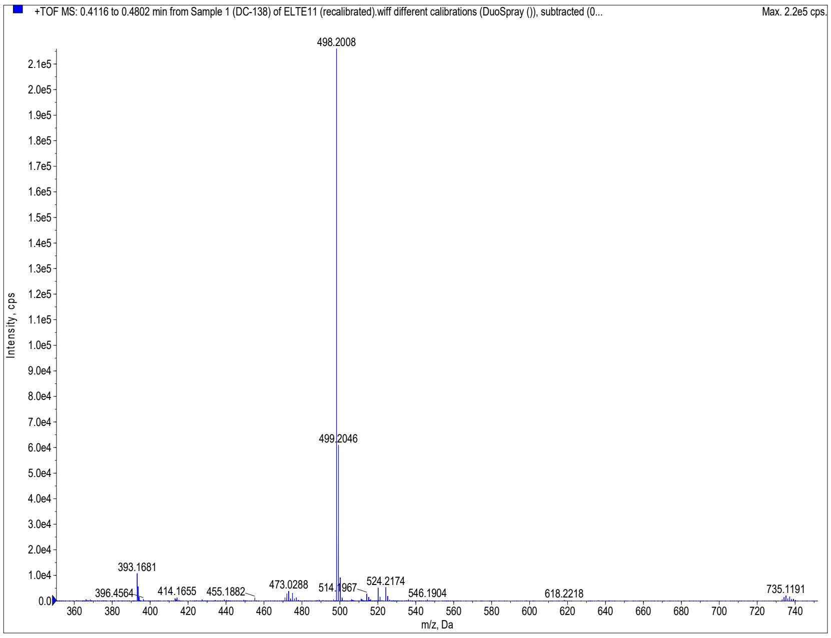

(E)-3-(4-((3-((6,7-bis(2-methoxyethoxy)quinazolin-4-yl)amino)phenyl)ethynyl)phenyl)-1-(3,4,5-trimethoxyphenyl)prop-2-en-1-one (**13**)	m/z calcd. for [$C_{40}H_{40}N_3O_8$]: 690.2815 [M+H]$^+$; found: 690.2797. mass error: 2.67 ppm

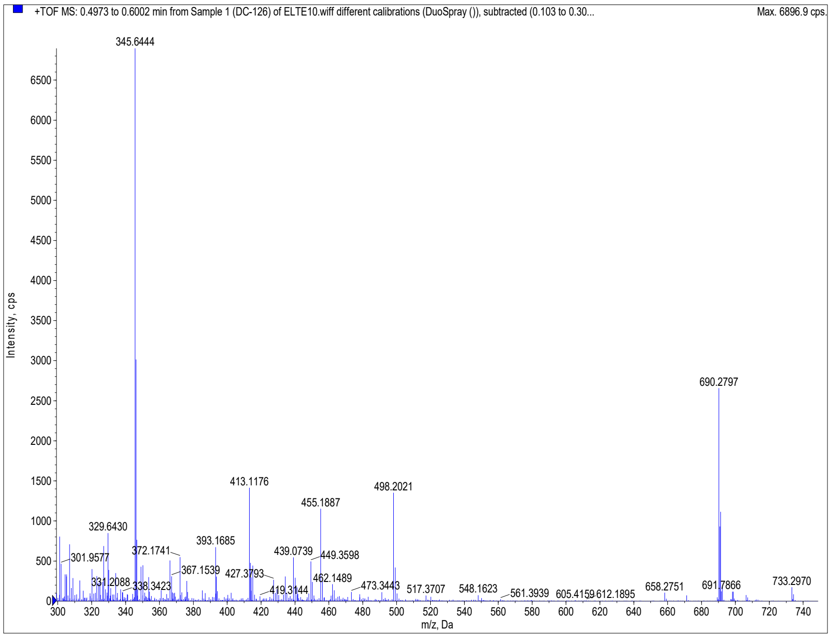

(E)-3-(2-((3-((6,7-bis(2-methoxyethoxy)quinazolin-4-yl)amino)phenyl)ethynyl)phenyl)-1-(3,4,5-trimethoxyphenyl)prop-2-en-1-one (**14**)	m/z calcd. for [$C_{40}H_{40}N_3O_8$]: 690.2815 [M+H]$^+$; found: 690.2798. mass error: 2.52 ppm

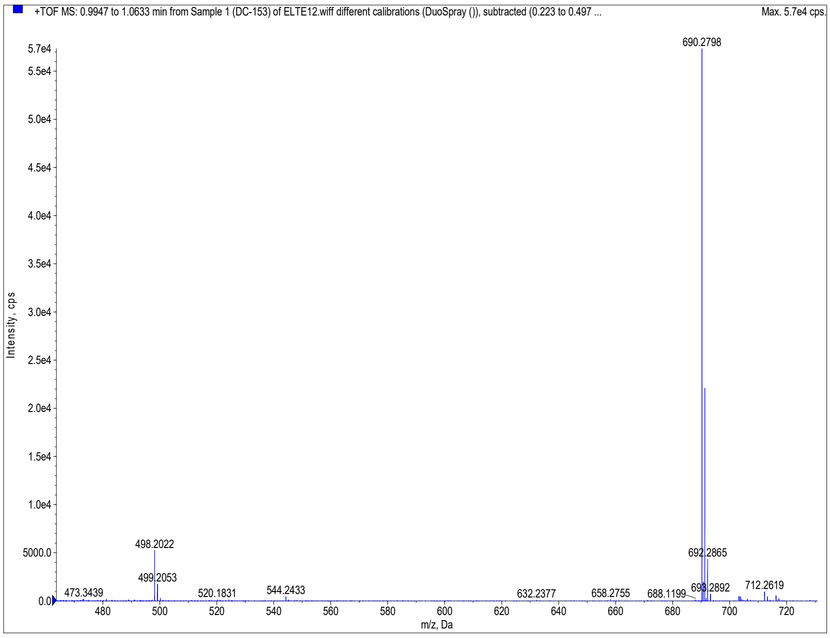

4 Biological evaluation

4.1 Celltiter-Glo Cell Viability Assay Data

Fadu after 24 hours of treatment (n=4)

Concentration (nM)	1		6a		13		14		15		1 + 15	
	**mean**	sd	**mean**	sd	**mean**	sd	**mean**	sd	**mean**	sd	**mean**	sd
10000	**70**	7	**25**	6	**86**	9	**78**	10	**77**	14	**61**	12
5000	**77**	10	**26**	6	**90**	10	**77**	13	**75**	8	**56**	5
2500	**89**	8	**44**	14	**92**	5	**76**	10	**93**	9	**68**	7
1250	**92**	7	**67**	11	**97**	8	**87**	6	**100**	9	**88**	4
625	**99**	4	**88**	11	**103**	7	**92**	3	**99**	9	**94**	9
312,5	**100**	3	**98**	4	**98**	9	**96**	6	**101**	3	**98**	8
156,25	**95**	4	**102**	7	**102**	5	**96**	5	**101**	3	**96**	4

Fadu after 72 hours of treatment and 72 hours of post-incubation (n=4)

Concentration (nM)	1		6a		13		14		15		1 + 15	
	**mean**	sd	**mean**	sd	**mean**	sd	**mean**	sd	**mean**	sd	**mean**	sd
10000	**18**	2	**0**	0	**0**	0	**0**	0	**2**	0	**2**	0
5000	**29**	3	**0**	0	**0**	0	**0**	0	**1**	0	**4**	1
2500	**42**	2	**0**	0	**0**	0	**0**	0	**36**	16	**8**	4
1250	**52**	1	**0**	0	**3**	2	**2**	2	**76**	13	**39**	2
625	**61**	4	**7**	4	**81**	4	**65**	5	**88**	11	**48**	6
312,5	**68**	7	**76**	7	**93**	3	**79**	12	**84**	14	**50**	8
156,25	**74**	5	**87**	3	**92**	4	**80**	10	**90**	11	**60**	13

Detroit 562 after 24 hours of treatment (n=4)

Concentration (nM)	1		6a		13		14		15		1 + 15	
	**mean**	sd	**mean**	sd	**mean**	sd	**mean**	sd	**mean**	sd	**mean**	sd
10000	**67**	4	**65**	12	**74**	8	**79**	10	**58**	1	**47**	3
5000	**71**	6	**68**	11	**80**	7	**80**	11	**75**	9	**63**	4
2500	**85**	6	**77**	7	**81**	9	**80**	9	**81**	4	**71**	5
1250	**91**	3	**78**	6	**87**	9	**83**	6	**95**	3	**80**	4
625	**91**	6	**92**	3	**99**	8	**91**	7	**102**	3	**86**	9
312,5	**96**	5	**104**	2	**101**	3	**98**	12	**104**	8	**89**	3
156,25	**96**	6	**103**	5	**102**	8	**106**	6	**103**	8	**86**	8

Detroit 562 after 72 hours of treatment and 72 hours of post-incubation (n=4)

Concentration (nM)	1		6a		13		14		15		1 + 15	
	**mean**	sd	**mean**	sd	**mean**	sd	**mean**	sd	**mean**	sd	**mean**	sd
10000	**28**	2	**0**	0	**0**	0	**0**	0	**1**	0	**0**	0
5000	**47**	3	**0**	0	**0**	0	**0**	0	**11**	9	**7**	2
2500	**64**	6	**0**	0	**0**	0	**0**	0	**71**	7	**43**	8
1250	**74**	3	**0**	0	**4**	4	**52**	7	**93**	6	**66**	9
625	**78**	8	**62**	6	**87**	10	**95**	5	**99**	7	**73**	5
312,5	**81**	6	**95**	8	**98**	6	**99**	7	**102**	6	**82**	20
156,25	**78**	6	**90**	4	**89**	9	**96**	13	**91**	11	**75**	19

SCC-25 after 24 hours of treatment (n=4)

Concentration (nM)	1		6a		13		14		15		1 + 15	
	**mean**	sd	**mean**	sd	**mean**	sd	**mean**	sd	**mean**	sd	**mean**	sd
10000	**57**	1	**24**	4	**87**	6	**76**	11	**44**	7	**19**	6
5000	**69**	3	**27**	3	**87**	7	**81**	7	**53**	4	**35**	2
2500	**73**	4	**35**	16	**93**	7	**80**	9	**85**	1	**62**	5
1250	**77**	2	**70**	7	**110**	8	**87**	14	**106**	4	**75**	4
625	**84**	5	**92**	12	**109**	3	**104**	7	**105**	4	**78**	1
312,5	**83**	6	**102**	8	**108**	4	**106**	5	**104**	3	**82**	4
156,25	**84**	5	**106**	12	**99**	7	**106**	5	**101**	3	**83**	8

SCC-25 after 72 hours of treatment and 72 hours of post-incubation (n=4)

Concentration (nM)	1		6a		13		14		15		1 + 15	
	**mean**	sd	**mean**	sd	**mean**	sd	**mean**	sd	**mean**	sd	**mean**	sd
10000	**11**	6	**0**	0	**0**	0	**0**	0	**0**	0	**0**	0
5000	**20**	9	**0**	0	**0**	0	**0**	0	**5**	1	**6**	1
2500	**29**	6	**0**	0	**18**	20	**1**	1	**63**	30	**18**	6
1250	**32**	6	**0**	0	**93**	24	**77**	22	**99**	5	**33**	9
625	**37**	7	**89**	12	**99**	12	**100**	15	**102**	7	**37**	9
312,5	**43**	12	**97**	7	**94**	3	**101**	14	**98**	9	**41**	13
156,25	**51**	18	**91**	6	**85**	8	**86**	7	**86**	12	**46**	20

4.2 *EGFR inhibition efficacy of compounds on Fadu, Detroit-562 and SCC-25 cells*

Inhibition of EGFR phosphorylation (pY1068) was investigated by western blot analysis (Figure S1). HNSCC cells were treated with the compounds at 5 μM for 4 h. 1 (erlotinib) resulted significant EGFR inhibition in all of the three HNSCC cell lines tested. This inhibitory effect was even further enhanced in combination with 15 (chalcone), although 15 did not show significant EGFR inhibition in alone. Despite the relatively high concentration (5 μM), none of the hybrids caused significant EGFR inhibition.

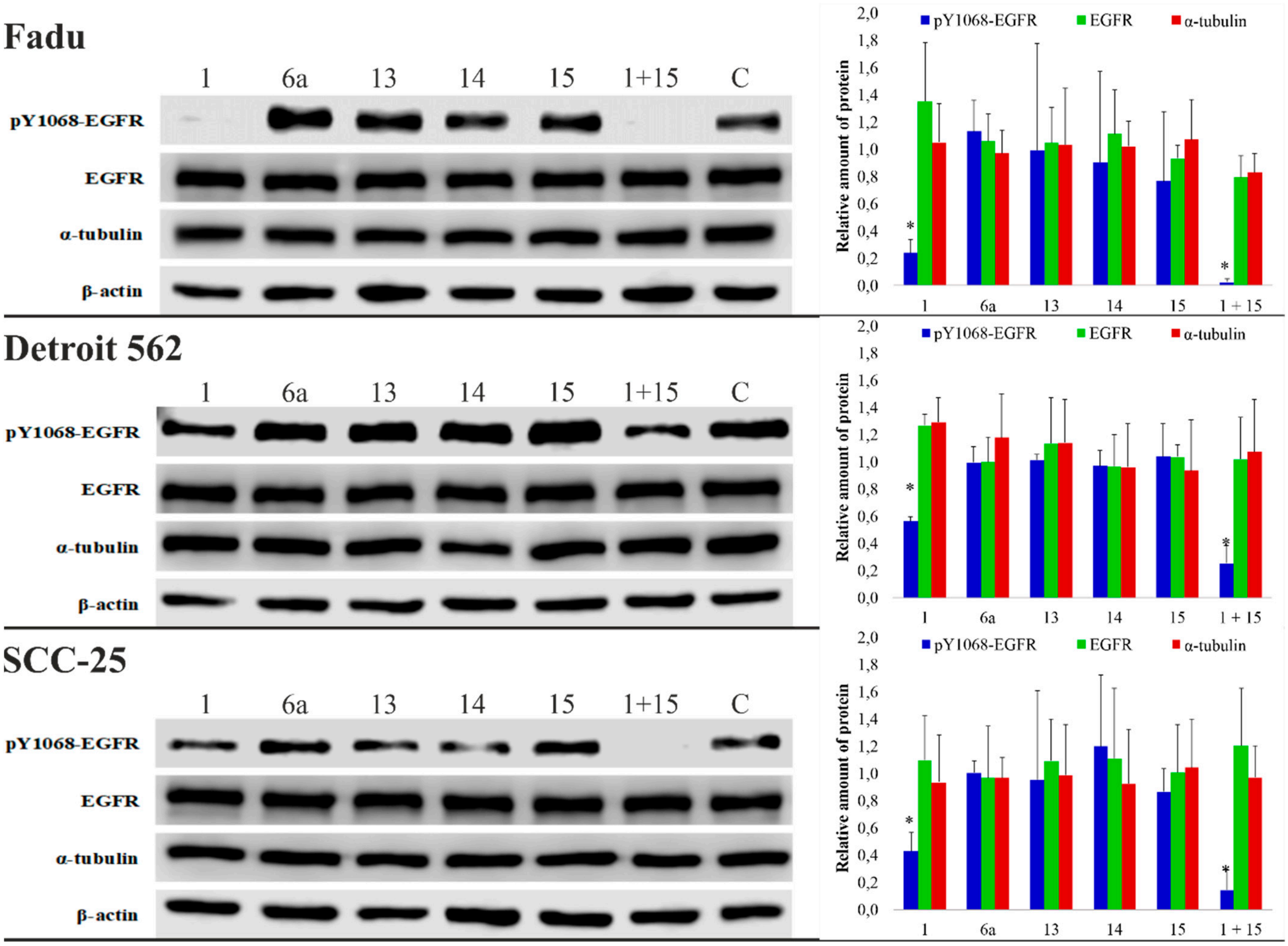

Figure S1. Changes of protein expression and phosphorylation after 1 (erlotinib), 6a, 13, 14, 15 (chalcone) and 1 + 15 treatments (4 h, 5 μM) in HNSCC cell lines. (A) Treated cells were subjected to western blot analysis with antibodies against pY1068-EGFR, EGFR, α-tubulin and the loading control, β-actin. (B) Densitometry analysis of pY1068-EGFR, EGFR and α-tubulin expression after treatments in FaDu, Detroit 562 and SCC25 cells. Densitometry analysis show the results of three independent experiments. The expressions of all proteins were compared to in the DMSO (dimethyl sulfoxide) treated negative controls (C), after normalization to β-actin. Data are presented as mean ± SD. Statistical analysis was performed by Student's t-test, in each cell line the expression of all proteins in all treated samples were compared to protein expression in DMSO treated samples $^*p < 0.05$.

Method: Cells were grown until 90% confluence in 6 well plates and were treated with 2.5 μM 1 (erlotinib), 6a, 13, 14, 15, 1 + 15 (1:1) and 0.1 % DMSO as vehicle in complete medium. After 4 h treatment, cells were washed with ice-cold PBS and lysed in RIPA buffer (50 mM Tris (pH 7.4), 150mM NaCl, 1% (V/V) NP-40, 0.5% (m/V) sodium-deoxycholate, 0.1% (m/V) sodium dodecyl sulphate, 2 mM EDTA, 2 mM EGTA, 1 mM dithiothreitol, phosphatase inhibitor cocktail (Merck, Kenilworth, NJ, USA) and protease inhibitor cocktail (Calbiochem) for 30 minutes on ice. Lysates were centrifuged with 13 000x g at 4°C for 15 minutes. 20 μg protein samples were

subjected to SDS-PAGE and electrotransferred to polyvinylidene-difluoride (PVDF) membranes. Membranes were incubated with the diluted primary antibodies at 4°C overnight, and with horse radish peroxidase (HRP) conjugated secondary antibodies for 1 h at room temperature. EGFR (clone D38B1, Cat. No. 4267, dilution 1:2000), pY1068-EGFR (clone D7A5, Cat. No. 3777, dilution 1:1000) and β-actin (clone D6A8, Cat. No. 8457, dilution 1:2000) monoclonal antibodies were purchased from Cell Signaling Technology (Danvers, MA, USA). Anti-mouse IgG (Cat. No. 7076, dilution 1:8000) and Anti-rabbit IgG (Cat. No. 7054, dilution 1:2000) secondary antibodies were purchased from Cell Signaling Technology. Bands were visualized by Enhanced Chemiluminescence (ECL) detection system (Perkin Elmer, Waltham, MA, USA) with LI-COR C-DiGit Chemiluminescence Western Blot Scanner (LI-COR Biosciences, Lincoln, NE, USA) and quantified by Image Studio™ Software (LI-COR Biosciences, Lincoln, NE, USA). Every experiment was carried out at least three times. Data were evaluated by MS Excel.

4.3 Inhibition of tubulin polymerization

Effect of the selected compounds on tubulin polymerization was measured by In Vitro Tubulin Polymerization Assay. Among the tested compounds, only **15** (chalcone) resulted in significant inhibition of tubulin polymerization (Figure S2). Hybrid **6a** had a much weaker effect, while **13** and **14** proved to be similar to the reference (0.1% DMSO containing reaction buffer).

Method: An absorbance-based In Vitro Tubulin Polymerization Assay Kit (Merck, cat. no.: 17-10194) was applied according to the instructions provided by the manufacturer, thereby nocodazole was used as positive control while paclitaxel as negative control at 10 μM. The tested compounds (**6a**, 13, **14** and **15**) were applied at 5 μM. Polymerization of tubulin in treatment-free buffer (containing 0.1% DMSO as vehicle) was served as reference. Absorbance was measured at 350 nm, data was collected in every 30 seconds during a 40 mins period using a BioTek Synergy 2 Multi-Mode Reader. Data from one representative experiment of two independent experiments are shown in Figure S2.

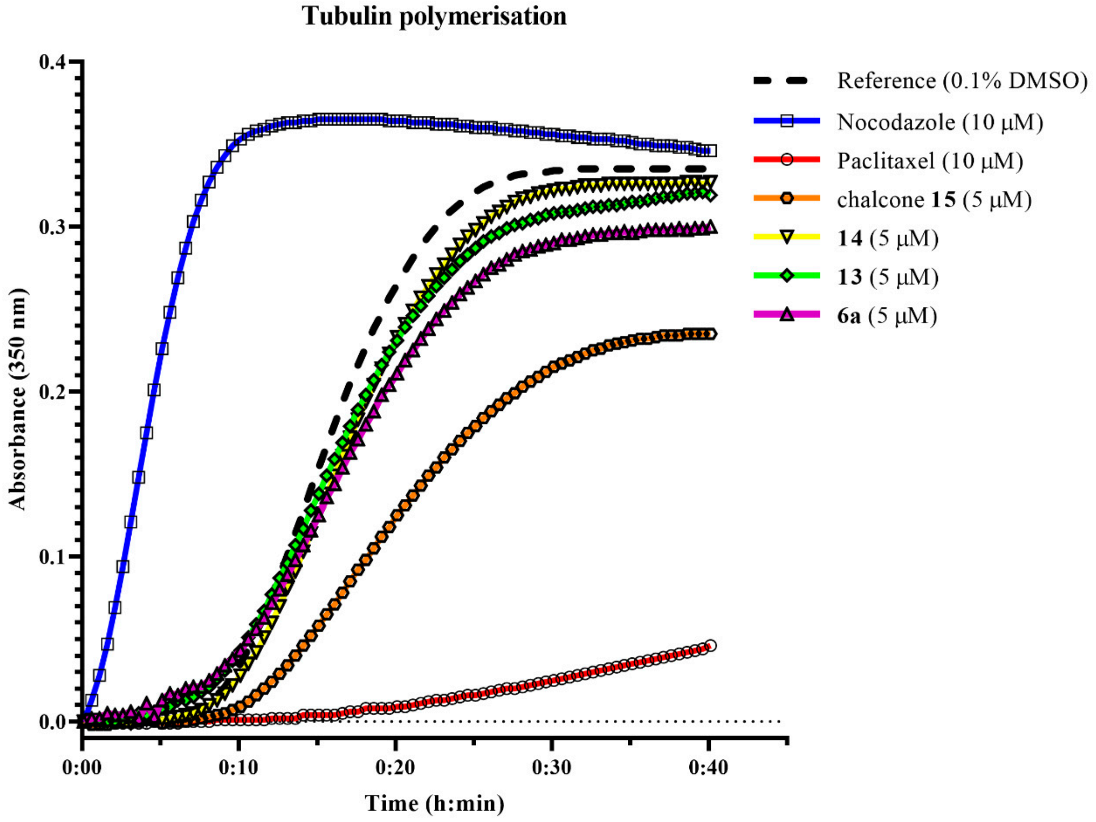

Figure S2. Effect of the hybrid molecules **6a**, **13** and **14** and chalcone **15** on tubulin polymerization. Nocodazole was applied as positive control and paclitaxel as negative control. Among the tested compounds, only **15** resulted in significant inhibition of tubulin polymerization.

4.4 *Glutathione assay*

Several anti-cancer compounds can induce oxidative stress, which may lead to programmed cell death, e.g.: apoptosis, necroptosis or ferroptosis. Reduction of GSH/GSSG ratio is a known marker for oxidative stress in cells. Among the tested compounds, **6a** caused the highest decrease in GSH/GSSG ratio, most significantly in Fadu and SCC-25 cells. A moderate decrease was observed in case of **13** and **14** similar to **15**. Interestingly, the GSH/GSSG ratio was less affected in Detroit 562 cells.

Method: To investigate the possible cell death mechanism caused by hybrid **6a**, **13** and **14** reduced/oxidized glutathione (GSH/GSSG) assay was performed on Fadu, Detroit 562 and SCC-25 cells using GSH/GSSG-Glo™ Assay (Promega). Cells were seeded into white 96-well plate at 5000 cells/well density and were incubated for 48 hours. Then, the cells were treated in duplicate with the compounds at 2.5 μM for 24 hours at 37 °C in CO_2 incubator. After treatment the assay was performed according to the instructions of the manufacturer, luminescence was measured by BioTek Synergy multimode reader and GSH/GSSG ratios were calculated.

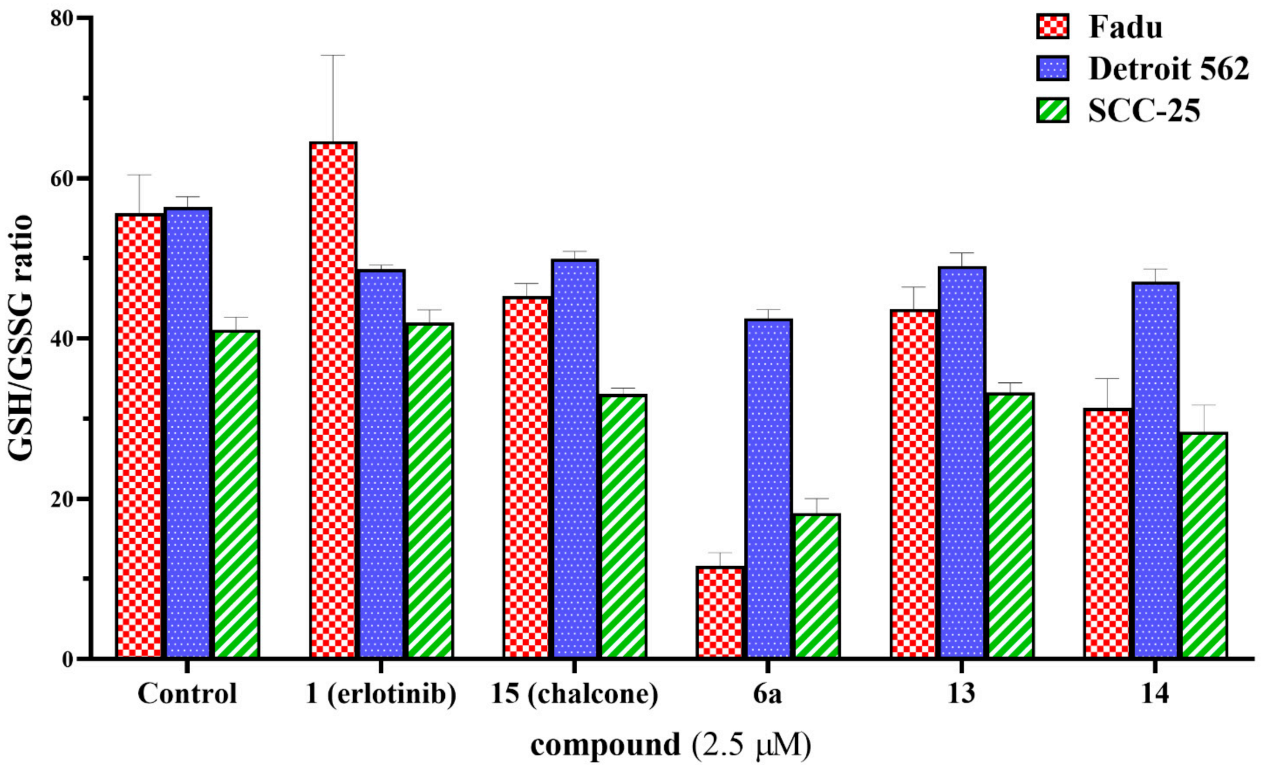

Figure S3. The reduced/oxidized glutathione (GSH/GSSG) ratio change of the cells after the treatment with the compounds. Cells were treated with the indicated compounds at 2.5 μM for 24 hours. Data represent the mean ± SD; n=2.

4.5 *Cell cycle analysis*

Cell cycle analysis revealed that **1** (Erlotinib) increases G0/G1 phase, and decreases S phase in Fadu, Detroit 562 and SCC-25 HNSCC cells after 18 hours of treatment. Effect of the most prominent hybrids (**6a**, **13** and **14**) on the cell cycle was not significant as in the case of **15** (chalcone).

Method: Cells were seeded at 30000 cell/well onto a flat bottom 24-well plate. After 48 h, cells were treated for 18 hours at 2.5 µM. Treating medium was removed, cells were washed with PBS and 200 µl trypsin was added per well. After 15 mins incubation 200 µl PBS was added, and detached cells were collected into 2 ml tubes. Tubes were centrifuged (350g, 4 mins) supernatant removed and 400 µl PBS was added, and tubes centrifuged again. Supernatant was discarded, and cell were fixed with 200 µl ice-cold 70% ethanol and tubes were gently vortexed twice. Samples were incubated at 4°C for 30 mins, followed by centrifugation and removing supernatant. 400 µl PBS was added, tubes centrifuged, and supernatant removed. 50 µl RNAs solution (50 µg/ml in PBS) was added per tubes, and incubated at 37°C for 10 mins. 100 µl PI solution (10 µg/ml in PBS) was added per tubes and incubated for 30 mins at ambient temperature. Cells were analysed with CytoFLEX Flow Cytometer by Beckman Coulter.

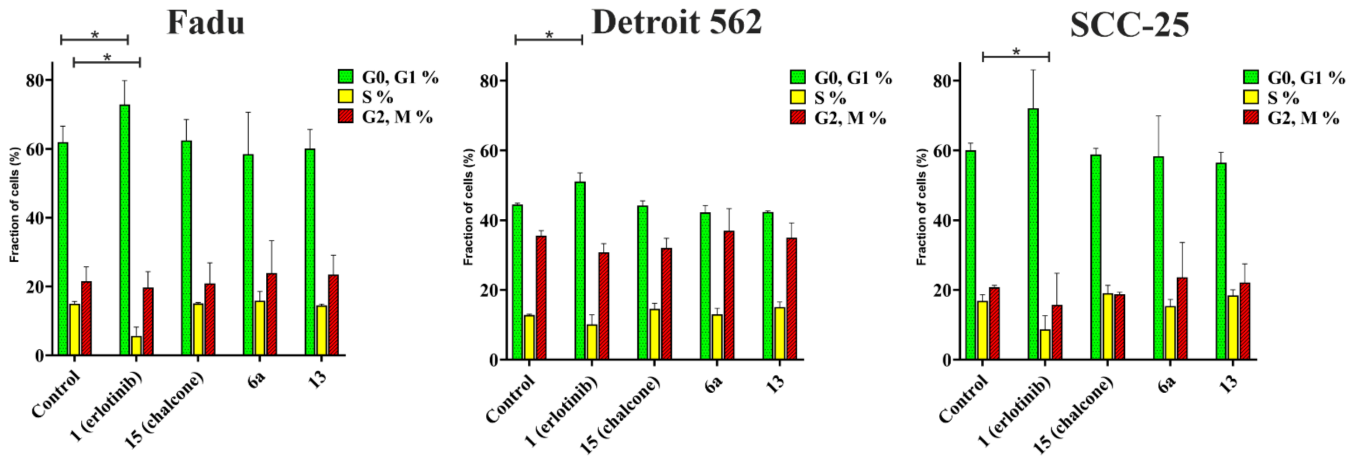

Figure S4. Cell cycle analysis. Hybrids (**6a**, **13**) and chalcone (**15**) have no significant effect on cell cycle after 18 hours of treatment at 2.5 µM concentration, however erlotinib (**1**) increased G0/G1 phase, and decrease S phase in all three HNSCC cells tested (Data represent the mean ± SD; n=2).

4.6 Galectin-3 puncta assay

Lysosomal membrane permeabilization (LMP) was investigated by galectin-3 puncta assay (Figure S5). Lysosome inhibitor DQ661 was used as positive control of LMP. Presence of galectin-3 puncta refers to lysosomal membrane damage. Expect DQ661, none of the tested compounds (2.5 μM, 24 h) induce galectin-3 puncta formation based on immunocytochemistry analysis of galectin-3.

Method: Experiment was carried out as previously described: DOI: 10.3390/ijms20225590

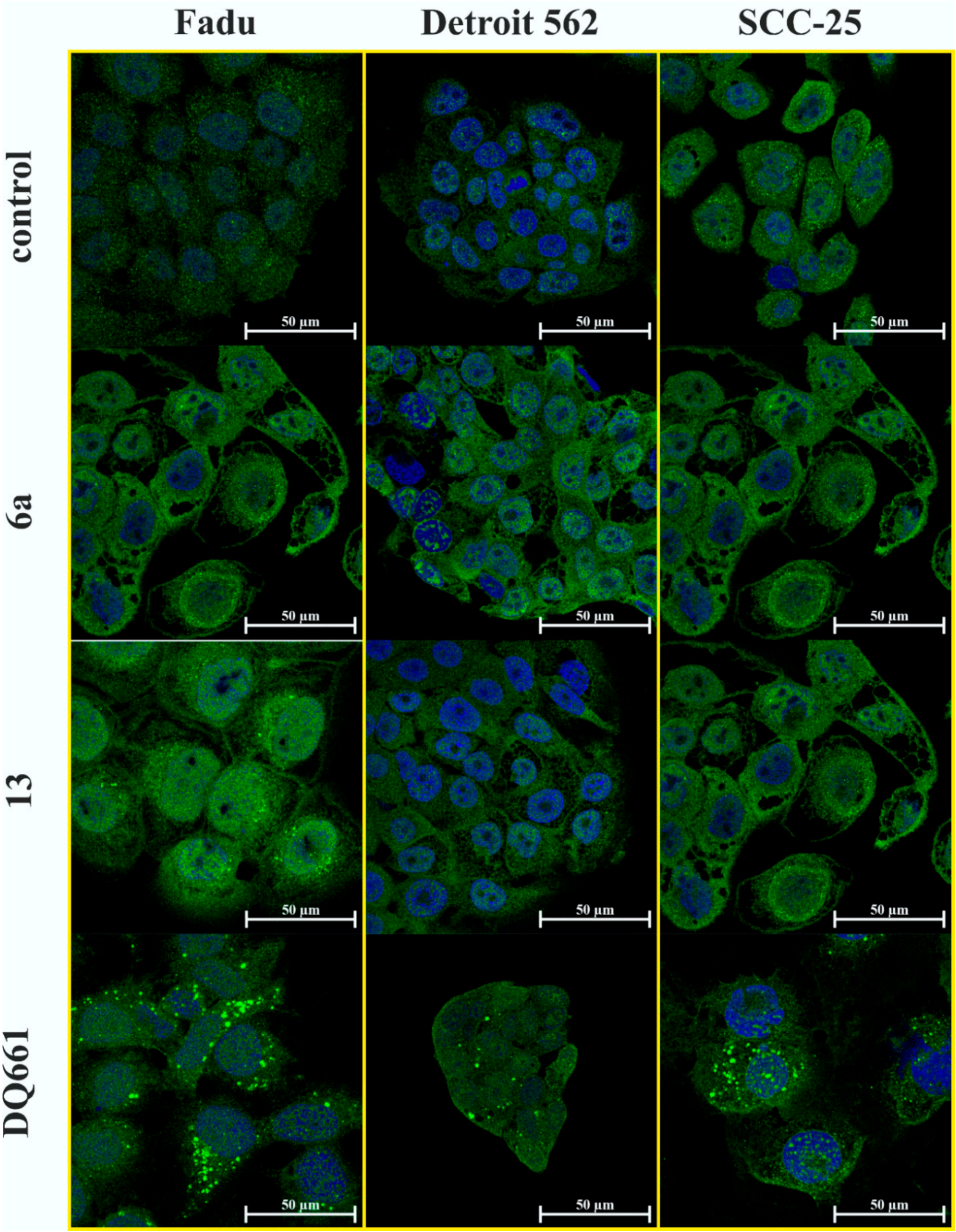

Figure S5. Galectin puncta assay for hybrids **6a** and **13**. Galectin-3 puncta formation indicates lysosomal membrane damage. DQ661 was used as positive control. Galectin-3: green (Alexa Fluor 488); nuclei: blue (DRAQ5).
